# Research on a multi-strategy enhanced parrot optimization algorithm STPO for Complex optimization problems and its applications

**DOI:** 10.1038/s41598-026-53263-3

**Published:** 2026-06-09

**Authors:** MingXuan Jian, GuoZhen Wu, BangLing Xiao

**Affiliations:** 1 School of Tianyou, East China Jiaotong University, Nanchang, 330000 China; 2 School of Electrical Engineering, East China Jiaotong University, Nanchang, 330013 China; 3 Tongji Medical University of HUST, Huazhong University of Science and Technology, WuHan, 430030 China

**Keywords:** Heuristic algorithm, STPO algorithm, Global optimization, Engineering design, Path planning, Engineering, Mathematics and computing

## Abstract

Metaheuristic optimization algorithms are widely used to tackle complex, high-dimensional, and nonlinear problems by mimicking natural or social behaviors, showing great potential for future development. Among them, the Parrot Optimization (PO) algorithm, inspired by the green-cheeked conure, exhibits strong adaptability. However, in high-dimensional scenarios, it often converges slowly and is prone to getting trapped in local optima. To address these limitations, this study proposes a multi-strategy enhanced parrot optimizer, termed STPO. STPO integrates an alert protection mechanism inspired by the Sparrow Search Algorithm, an experience exchange strategy, and a worst-guided differential scale perturbation operator to improve population guidance, strengthen perturbation-based search, facilitate the transition from exploration to exploitation, and enhance convergence stability. Comprehensive experiments on the CEC2017 and CEC2022 benchmark suites demonstrate that STPO achieves highly competitive average rankings. Specifically, on CEC2017, STPO obtains average ranks of 1.76, 1.21, 1.21, and 1.46 under 10-, 30-, 50-, and 100-dimensional settings, respectively. On CEC2022, STPO achieves average ranks of 2.15 and 1.74 under 10- and 20-dimensional settings, respectively. These quantitative results indicate that STPO provides stable and accurate optimization performance compared with the twelve competing algorithms. Furthermore, statistical tests, ablation experiments, population diversity analysis, and exploration–exploitation analysis are conducted to further examine the effectiveness and dynamic search behavior of STPO. When applied to five classical engineering design problems and mobile robot path planning tasks, STPO also achieves competitive solution accuracy and convergence behavior, further confirming its applicability to constrained engineering optimization and practical path planning scenarios. The Source code for this work is openly available at https://github.com/MingXuanJian/STPO.git.

## Introduction

As society continues to advance, the challenges we seek to address in real life grow increasingly complex to meet the rising demands for a better life. When confronted with high-dimensional and nonlinear optimization problems, traditional linear or nonlinear programming methods often become inefficient or difficult to apply^1^. Consequently, researchers have increasingly turned to computational paradigms that can provide near-optimal solutions at acceptable computational costs.

The term “metaheuristic” was established in the 1980 s and 1990 s, referring to a class of advanced algorithms that do not rely on specific problem models^2^. The first generation of metaheuristic algorithms included genetic algorithms (GA)^3^, simulated annealing (SA)^4^, ant colony optimization (ACO)^5^, and particle swarm optimization (PSO)^6^. These algorithms laid the foundation for the subsequent rapid development of metaheuristic optimization.

Against the backdrop of continuous improvement in computational power and increasing interdisciplinary integration, metaheuristic algorithms have been widely developed and applied^7^. Early studies proposed many algorithms inspired by biological and natural mechanisms, such as the Lion Optimization Algorithm (LOA)^8^ proposed in 2016 and the Mayfly Optimization Algorithm (MOA)^9^ introduced in 2020. Meanwhile, guided by the “No Free Lunch” (NFL) theorem proposed by Wolpert and Macready^10^, researchers have increasingly focused not only on designing new optimizers but also on improving existing algorithms for specific problem domains. Strategies such as chaotic mapping, opposition-based learning, differential mutation, and adaptive parameter control^11–14^ have been widely utilized to improve the search behavior of metaheuristic algorithms. These strategies are usually designed to improve population-level search behavior, regulate the exploration–exploitation tradeoff, and enhance convergence performance under specific optimization conditions. For example, based on transformation functions, Emary et al. developed the Binary Gray Wolf Optimization (BGWO) algorithm^15^, which demonstrated competitive performance in feature selection tasks. Zeng et al. proposed Dynamic Neighborhood Switching Particle Swarm Optimization (DNSPSO)^16^, which alleviates premature convergence through adaptive neighborhood switching. Qi et al. introduced the Enhanced Ant Colony Optimization algorithm (XMACO)^17^, where directed crossover and mutation operators were employed to strengthen the search capability in high-dimensional threshold spaces, and improved segmentation performance was reported in COVID-19 medical image segmentation tasks.

Recent studies in engineering-oriented metaheuristic optimization highlight the growing importance of hybridization and mechanism-level enhancement for improving performance in constrained real-world problems. For instance, the Red-Billed Blue Magpie Optimization (RBMOAO)^18^ algorithm combines the Red-Billed Blue Magpie Optimizer with the Aquila Optimizer to enhance numerical optimization and engineering problem-solving. Similarly, the Dynamic Gold Rush Optimizer^19^ introduces worker adaptation and salp navigation mechanisms to improve search capabilities. These advancements underscore the increasing focus on cooperative mechanisms, adaptive search behaviors, and robustness in constrained optimization settings. Furthermore, recent work in mechanism-level enhancement has gained traction as a crucial research direction for developing more robust and adaptive optimizers. Notable examples include the trend-aware mechanism (TAM)^20^, which leverages historical position information to guide metaheuristic algorithms, and Competitive Framework Differential Evolution (CFDE)^21^, which integrates competitive learning to enhance the search capacity of SHADE-based algorithms. Additionally, PTMCO-FS^22^ employs a multi-task collaborative optimization framework to address high-dimensional feature selection challenges, demonstrating the power of collaborative search and knowledge-guided optimization in complex environments.

As a recently proposed metaheuristic optimizer, the Parrot Optimization (PO) algorithm^23^ simulates four representative behaviors of the green-cheeked conure, namely foraging, staying, communicating, and fear of strangers. Different from many classical metaheuristic algorithms whose exploration and exploitation patterns are largely predefined, PO employs a behavior-switching search framework to dynamically guide population evolution. This flexible behavioral structure provides PO with promising adaptability and makes it a suitable baseline for mechanism-level enhancement without completely reconstructing the original algorithmic framework.

Among the numerous metaheuristic algorithms developed over the past few decades, PO is selected as the research basis in this study mainly for three reasons. First, its multi-behavior search mechanism enables an adaptive transition from exploration to exploitation, which is beneficial for solving complex optimization problems. Second, as a relatively new optimizer, PO still suffers from several limitations in high-dimensional, multimodal, and strongly constrained environments, such as slow convergence, insufficient effective search guidance, premature convergence, and a tendency to become trapped in local optima. These weaknesses make PO a meaningful candidate for further improvement. Third, the modular behavioral structure of PO provides a suitable interface for incorporating complementary mechanisms related to escape guidance, population information exchange, and perturbation-based search. Therefore, improving PO is not only practically necessary but also structurally suitable for constructing a cooperative multi-strategy optimizer.

To address these limitations, several improved PO algorithms have been developed to enhance search performance and solution efficiency. For instance, Li et al. proposed the Improved Parrot Optimizer (IPO)^24^, which incorporates the aerial exploration strategy from the Arctic Puffin Optimization algorithm to enhance global search capability. Huang et al. proposed the Multi-Strategy Parrot Optimization Algorithm (MIPO)^25^, which improves algorithmic stability and robustness in complex search spaces by integrating multiple search mechanisms. Yang et al. proposed the Chaotic-Gaussian-Barycenter Parrot Optimization (CGBPO)^26^, which combines logistic chaotic initialization^27^, Gaussian mutation^28^, and centroid opposition-based learning^29^ to improve population diversity and alleviate premature convergence. Adegboye et al. proposed SNCPO^30^, which integrates SSA chain navigation^31^ with CSO competitive learning^32^ to balance exploration and exploitation.

**Fig. 1:**
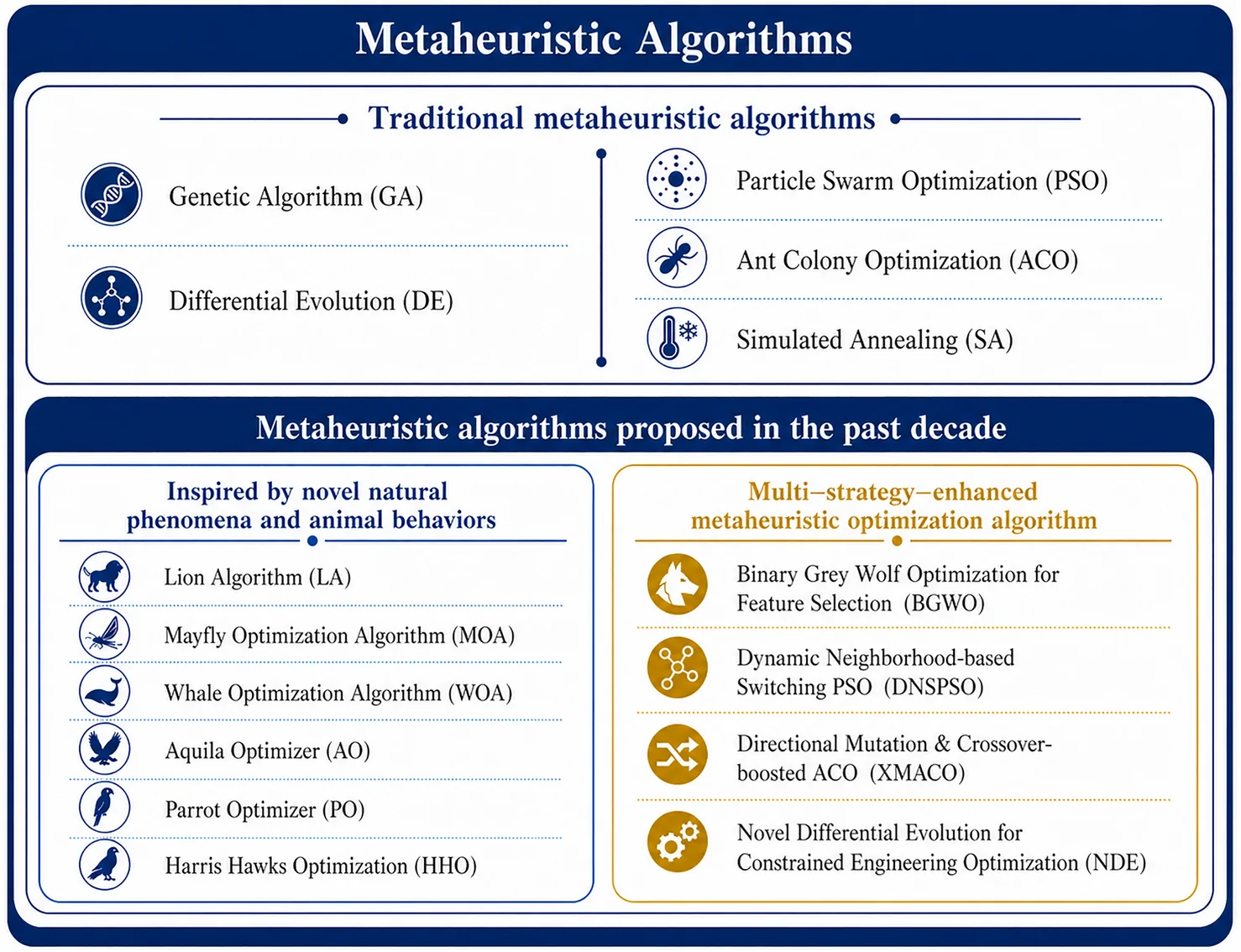
Classification of metaheuristic algorithms.

Although these PO variants have improved the original algorithm from different perspectives, most of them emphasize specific enhancement routes, such as initialization improvement, mutation enhancement, opposition-based learning, or competitive learning. A unified PO-based framework that simultaneously considers alert-driven escape, peer-to-peer experience sharing, and worst-guided differential perturbation remains insufficiently investigated. This gap motivates the proposed STPO algorithm, in which complementary mechanisms are organized within the PO behavioral framework to improve population guidance, perturbation-based search, and convergence stability while supporting an effective transition from exploration to exploitation.Different from a straightforward hybridization that merely combines several existing operators, the originality of STPO lies in its behavior-aware cooperative enhancement of the PO framework. Specifically, the proposed method assigns different search roles to different PO behavioral branches: APM is used as a state-triggered escape mechanism for disadvantaged individuals, EES provides peer-to-peer information diffusion during communication, and DSP introduces worst-guided perturbation to strengthen escape from local optimum regions. Therefore, STPO is designed as a coordinated search framework rather than a simple accumulation of independent improvement tricks. This design clarifies the functional division and interaction of the three strategies within the original PO behavioral structure (Fig. 1).

Therefore, the main contributions of this study are summarized as follows:

- A multi-strategy enhanced PO framework, termed STPO, is proposed. Instead of simply stacking several operators, STPO embeds complementary mechanisms into the behavioral structure of PO to improve the coordination between exploration and exploitation.
- An alert protection mechanism inspired by the Sparrow Search Algorithm is introduced to guide disadvantaged individuals away from low-quality regions, thereby reducing search stagnation and premature convergence.
- An experience exchange strategy is designed to promote peer-to-peer information sharing among individuals. This mechanism improves population-level information diffusion and alleviates the risk of premature search aggregation caused by over-reliance on a single elite solution.
- A worst-guided differential scale perturbation strategy is introduced to provide additional perturbation-based exploration and improve the ability of individuals to escape local optimum regions. This mechanism helps STPO explore promising regions in the early search stage and then supports faster convergence through greedy selection.
- Extensive experiments are conducted on CEC2017, CEC2022, five constrained engineering design problems, and mobile robot path planning tasks. In addition to statistical tests and ablation studies, population diversity and exploration–exploitation analyses are further provided to reveal the dynamic search behavior of STPO and verify the effectiveness of the proposed mechanisms.

The remainder of this paper is organized as follows:

Section 1 introduces the mathematical model of PO; Sect. 2 describes the proposed improved PO algorithm; Sect. 3 focuses on CEC numerical experiments and statistical analysis; Sect. 4 presents experiments and analyses for typical engineering design problems; Sect. 5 demonstrates the application of STPO to the practical problem of route planning for mobile robots. Finally, the paper concludes with a summary and suggests several topics for further research.

## The basic principles of PO algorithm

The inspiration was derived from the four key characteristics of the green-cheeked conure in its foraging, standing, interaction, and visitor alerting behaviors, where Lian et al. introduced a new meta-heuristic optimization algorithm in 2024, namely, the Parrot Optimizer (PO).

The PO algorithm achieves dynamic switching by randomly activating different behavioral models, which differs significantly from typical metaheuristic algorithms that use predefined modes of exploration and optimization. This random evolutionary mechanism not only maintains high population diversity, but also increases the algorithm’s ability to escape local optima.

### Population initialization

The population size is set to *N*, and the search space is bounded by *lb* (lower bound) and *ub* (upper bound). The initialization scheme of the proposed $\textrm{PO}$ algorithm is given as follows:

1 $$\begin{aligned} X_i^{0}=lb+\operatorname {rand}(0,1)\cdot (ub-lb),\quad i=1,2,3,\ldots ,N \end{aligned}$$

In Eq. (1), $\textrm{rand}(0,1)$ denotes a uniformly distributed random number sampled from the interval *[0,1]*, which is used to generate random perturbations within the feasible region. Using Eq. (1), the algorithm can randomly generate the initial positions of *N* population individuals within the specified lower and upper bounds, thereby ensuring good coverage and diversity of the initial solutions in the search space.

### Foraging behavior

Parrots’ foraging behavior constitutes a crucial aspect of their activities. During the search for food, they primarily estimate the approximate location of food by observing its position or considering their owner’s location, then fly toward their respective positions. In the PO algorithm, this behavior is translated into an individual’s strategy for searching for the optimal solution, with its positional movement following the formula below:

2 $$\begin{aligned} X_{i}^{t+1} =\left( X_{i}^{t}-X_{\textrm{best}}\right) \cdot \textrm{Levy}(D) +\textrm{rand}(0,1)\cdot \left( 1-\frac{t}{T}\right) ^{\frac{2t}{T}}\cdot X_{\textrm{mean}}^{t} \end{aligned}$$

In Eq. (2), $X_i^{t}$ denotes the position of the *i*-th individual at iteration *t*, and $X_i^{t+1}$ denotes the updated position after the predation behavior. $X_{\textrm{best}}$ is the current global best position found by the population, and $X_{\textrm{mean}}^{t}$ is the mean position of all individuals at iteration *t*, which serves as an information reference when the population searches for food. The term $\textrm{Levy}(D)$ represents a random step length following the Lévy distribution, constructed based on the Mantegna algorithm to model long-distance jumps during foraging; *T* denotes the maximum number of iterations. The term $\left( 1-\frac{t}{T}\right) ^{\frac{2t}{T}}$ is an iteration-dependent attenuation factor, encouraging global exploration in the early stage and gradually shifting toward local exploitation in the later stage. Using Eq. (2), the algorithm simulates how parrots adjust their positions while searching for food, enabling effective exploration of regions that may contain the optimal solution.

The average position of the current population can be expressed by the following formula:

3 $$\begin{aligned} X_{\textrm{mean}}^{t}=\frac{1}{N}\sum _{k=1}^{N} X_{k}^{t} \end{aligned}$$

In Eq. (3), $\sum _{k=1}^{N} X_k^{t}$ denotes the summation of the position vectors of all individuals at iteration *t*; dividing by *N* yields the mean position.

We can derive the $\textrm{Levy}$ distribution by applying the rules in the equation, where *γ* is set to *1.5*.

4 $$\begin{aligned} \left\{ \begin{array}{l} \text {Levy}(D) = \frac{\mu \cdot \sigma }{|\nu |^{1/\gamma }} \\ \mu \sim N(0, D) \\ \nu \sim N(0, D) \\ \sigma = \left( \frac{\Gamma (1 + r) \cdot \sin \left( \frac{\pi \gamma }{2}\right) }{\Gamma \left( \frac{1 + r}{2}\right) \cdot \gamma \cdot 2^{\frac{1 + r}{2}}} \right) ^{\gamma + 1} \end{array} \right. \end{aligned}$$

In Eq. (4), *μ* and *ν* are random variables sampled from a Gaussian distribution with mean *0* and variance *D*, which are used to generate the random perturbations required by the $\textrm{Levy}$ distribution. *γ* is the stability index of the $\textrm{Levy}$ distribution and is set to *1.5* to control the degree of heavy-tailed behavior. The parameter *σ* is the scale parameter of the $\textrm{Levy}$ distribution; it is jointly determined by the Gamma function $\Gamma (\cdot )$ and the trigonometric term, and it is used to normalize the step size so that the generated $\textrm{Levy}$ steps have a reasonable jump magnitude.

### Staying behavior

Parrots are highly social creatures, and their perching behavior primarily involves suddenly flying to any part of their owner’s body and remaining motionless there for a period of time. This process can be described as:

5 $$\begin{aligned} X_i^{t+1}=X_i^{t}+X_{\textrm{best}}\cdot \textrm{Levy}(D)+\textrm{rand}(0,1)\cdot \textrm{ones}(1,D) \end{aligned}$$

In Eq. (5), $\textrm{ones}(1,D)$ denotes an all-ones vector of dimension *D*. The term $X_{\textrm{best}}\cdot \textrm{Levy}(D)$ represents the process in which the parrot flies toward the host, while $\textrm{rand}(0,1)\cdot \textrm{ones}(1,D)$ represents the process in which the parrot randomly stays at some position on the host.

### Communicating behavior

Parrots are inherently social creatures, and one of their defining characteristics is the close communication within their flocks. Through information exchange within the group, each individual can learn the behavioral patterns of others. This communication includes signals that indicate whether to fly toward the flock or away from it. In PO, assuming both behaviors occur with equal probability, the current population’s average position represents the flock’s center. This process can be expressed as:

6 $$\begin{aligned} X_i^{t+1}= {\left\{ \begin{array}{ll} 0.2\,\textrm{rand}(0,1)\left( 1-\dfrac{t}{T}\right) \left( X_i^{t}-X_{\textrm{mean}}^{t}\right) , & P \le 0.5,\\ 0.2\,\textrm{rand}(0,1)\exp \!\left( -\dfrac{t}{\textrm{rand}(0,1)\,T}\right) , & P> 0.5. \end{array}\right. } \end{aligned}$$

According to Eq. (6), $0.2\cdot \textrm{rand}(0,1)\cdot \left( 1-\frac{t}{T}\right) \cdot \left( X_i^{t}-X_{\textrm{mean}}^{t}\right)$ represents the process in which an individual leaves immediately after communication, which occurs when *P łe 0.5*. In contrast, $0.2\cdot \textrm{rand}(0,1)\cdot \exp \!\left( -\frac{t}{\textrm{rand}(0,1)\cdot T}\right)$ represents the process in which an individual joins the parrot group for communication, which occurs when *P> 0.5*. Both behaviors are feasible; therefore, one can use a random number generated in *[0,1]* to implement this choice.

### Fear of strangers

Generally speaking, birds exhibit a natural fear of strangers, and parrots are no exception. They act in a way that makes them feel protected–staying away from strangers and seeking cover by staying close to their owners. This process can be expressed as:

7 $$\begin{aligned} {\begin{matrix} X_i^{t+1} & = X_i^{t} + a\left( X_{\textrm{best}}-X_i^{t}\right) - b\left( X_i^{t}-X_{\textrm{best}}\right) ,\\ a & = \textrm{rand}(0,1)\cos \!\left( 0.5\pi \frac{t}{T}\right) ,\qquad b = \cos \!\left( \textrm{rand}(0,1)\pi \right) \left( \frac{t}{T}\right) ^{\frac{2}{T}} \end{matrix}} \end{aligned}$$

In Eq. (7), $\textrm{rand}(0,1)\cdot \cos \!\left( 0.5\pi \cdot \frac{t}{T}\right) \cdot \left( X_{\textrm{best}}-X_i^{t}\right)$ represents the process in which the parrot reorients itself and flies toward the host, whereas $\cos \!\left( \textrm{rand}(0,1)\cdot \pi \right) \cdot \left( \frac{t}{T}\right) ^{\frac{2}{T}}\cdot \left( X_i^{t}-X_{\textrm{best}}\right)$ represents the process of moving away from a stranger.

**Fig. 2:**
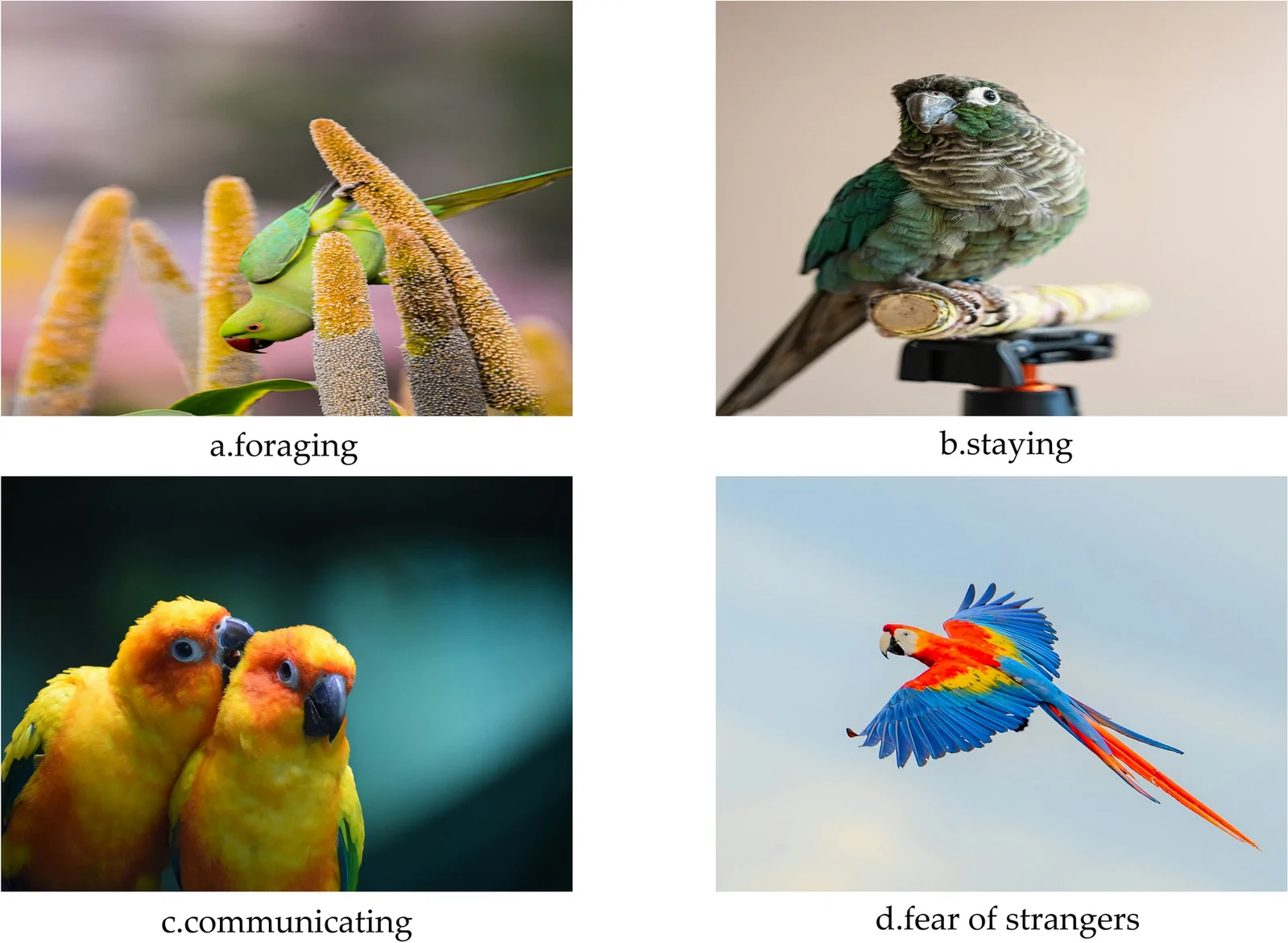
Four core behaviors of parrots.

Figure 2 illustrates the four behaviors corresponding to the parrot, which respectively correspond to the four different strategies in the original text.

## The proposed STPO algorithm

Slow convergence speed and susceptibility to local optima are significant challenges encountered by the Parrot (PO) algorithm when tackling complex optimization problems. To further enhance the PO algorithm’s performance while addressing its limitations in solving practical application and engineering design problems, an improved STPO algorithm is proposed. This improvement incorporates three key strategies: the alarm mechanism from the Sparrow algorithm, an experience exchange strategy, and worst-guided differential scale perturbation.

Each strategy is introduced with a specific motivation. First, APM is designed to guide low-quality or disadvantaged individuals away from unfavorable regions, thereby reducing stagnation and improving the probability of escaping local optima. Second, EES promotes peer-to-peer information exchange among individuals, which helps enhance population diversity and avoids excessive dependence on a single global best solution. Third, DSP uses the positional difference between individuals and the worst solution to generate adaptive perturbations, thereby strengthening global exploration and improving the ability to jump out of local optimum basins.In addition, STPO does not restrict the application of Levy flight solely to the foraging behavior of the original PO. Instead, Levy-based perturbation is flexibly combined with different update paths to facilitate a dynamic transition from broad early-stage exploration to focused later-stage exploitation, thereby improving convergence stability.More importantly, APM, EES, and DSP are not independently stacked operators, but complementary components embedded in the behavior-switching framework of PO. APM reduces stagnation of inferior individuals, EES enhances population-level information exchange, and DSP strengthens perturbation-based escape from local optima. Through their cooperation with boundary control, greedy selection, and global-best updating, STPO achieves a more balanced search process in terms of exploration, information diffusion, and convergence refinement.

### Sparrow algorithm’s alert protection mechanism (APM)

In STPO, to address the stagnation behavior and the behavior of remaining near strangers in the PO algorithm, the alertness mechanism of the Sparrow Search Algorithm is introduced^33^. Intuitively, when the fitness value of a certain parrot individual is significantly worse than the current global optimal solution, it can be regarded as being in a “danger” or “edge” state, making it more susceptible to predator attacks. In such a case, guidance from the alert individual is required to help it quickly move away from the unfavorable region and approach the vicinity of the global optimum, thereby avoiding prolonged stagnation in low-quality regions. Suppose that at iteration *t*, the position of the *i*-th individual is $X_i^{t} \in \mathbb {R}^{D}$, and its fitness is $f_i^{t}$. The global best position and fitness are denoted by $X_{t}^{s}$ and $f_{t}^{s} = \min _{i} f_i^{t}$, respectively. The alertness mechanism performs stage-wise updates according to the dominance status of individuals:

When an individual’s fitness is inferior to the global best ($f_i^{t}> f_g^{t}$), the individual performs random exploration in the vicinity of the global optimum, as shown in Eq. (8). This mechanism can significantly reduce the probability that disadvantaged individuals remain in low-quality regions, thereby improving the overall convergence speed.

8 $$\begin{aligned} X_i^{t+1}=X_g^{t}+n_i^{t}\odot \left| X_i^{t}-X_g^{t}\right| ,\quad n_i^{t}\sim \mathcal {N}\!\left( 0,\textbf{I}_{D}\right) \end{aligned}$$

According to Eq. (8), $X_i^{t+1}$ denotes the position update of the *i*-th parrot individual at iteration *t+1*. This update is based on the global best position at iteration *t*, and combines the random perturbation vector $n_i^{t}$, which follows a standard multivariate normal distribution, with the element-wise absolute difference between the current position and the global best position, $\left| X_i^{t}-X_g^{t}\right|$, through the Hadamard product (*⊙*)^34^, thereby dynamically adjusting the position. $\mathcal {N}(0,\textbf{I}_{D})$ denotes a *D*-dimensional multivariate normal distribution with zero mean vector and identity covariance matrix. In short, $f_i^{t}> f_g^{t}$ indicates that the parrot individual has inferior fitness compared to the global best and is located at the edge of the population, making it more vulnerable to predator attacks; this position update rule is designed to guide it quickly toward the global optimum region and prevent prolonged stagnation in low-quality areas.

When an individual’s fitness is not worse than the global best ($f_i^{t} \le f_g^{t}$), to avoid premature convergence while maintaining a certain degree of exploration capability, an update strategy that combines $\textrm{Levy}$ flight with iteration-decaying random perturbation is adopted, as shown in Eq. (9). This update mechanism helps expand the search range in the early stages of iteration and gradually enhances convergence in the later stages through the decay term.

9 $$\begin{aligned} X_i^{t+1}=X_i^{t}+X_g^{t}\odot \textrm{Levy}(D)+\xi _i^{t}\left( 1-\frac{t}{T}\right) \textrm{ones}(1,D),\quad \xi _i^{t}\sim \mathcal {N}(0,1) \end{aligned}$$

In Eq. (9), $\xi _i^{t}$ is a random perturbation term drawn from a standard normal distribution, and $\left( 1-\frac{t}{T}\right)$ is a coefficient that decays linearly as the iteration index increases. This coefficient allows the perturbation to maintain a relatively large magnitude in the early iterations to enlarge the search range, and then gradually weakens in later iterations to enhance convergence. The term $\textrm{ones}(1,D)$ ensures that the perturbation is applied to every dimension of the search space. By combining $\textrm{Levy}$ flight with a decaying perturbation, this update mechanism maintains strong global exploration in the early stage while accelerating convergence toward the global optimum in the later stage, thereby effectively avoiding premature convergence.

### Experience exchange strategy (EES)

In the STPO algorithm, the experience exchange strategy enhances the diversity of the search process through cooperation and information sharing among particles, thereby helping the algorithm avoid being trapped in local optima. For the *i*-th individual, another distinct individual *r ≠ i* is randomly selected, and their positional information is fused to update the solution^35^, as shown in Eq. (10).

10 $$\begin{aligned} X_i^{t+1}=\frac{X_i^{t}+X_r^{t}}{2},\quad r\in \{1,2,\ldots ,N\},\ r\ne i \end{aligned}$$

According to Eq. (10), $X_i^{t+1}$ denotes the updated position of the *i*-th parrot individual at iteration *t+1*. This position is obtained by taking the arithmetic mean of its current position at iteration *t*, $X_i^{t}$, and the current position of another randomly selected distinct individual, $X_r^{t}$. Here, $r \in \{1,2,\ldots ,N\}$ and *r ≠ i*, which ensures that information interaction occurs only between different individuals.

### Differential scale perturbation (DSP)

Differential evolution constructs new solutions using difference vectors to enhance search capability^36^, and its typical mutation form can be written as Eq. (11).

11 $$\begin{aligned} V_i^{t}=X_{r1}^{t}+F\cdot \left( X_{r2}^{t}-X_{r3}^{t}\right) ,\quad r1 \ne r2 \ne r3 \ne i \end{aligned}$$

Eq. (11) is the typical mutation form of differential evolution. Here, $V_i^{t}$ denotes the mutant vector of the *i*-th parrot individual at iteration *t*, and $X_{r1}^{t}$, $X_{r2}^{t}$, and $X_{r3}^{t}$ are the current positions of three randomly selected and mutually distinct parrot individuals. *F* is the scaling factor used to control the step size of the mutant vector. By computing and scaling the position differences among randomly selected individuals and then applying a weighted update, Eq. (11) provides the basic mutation idea for the worst-guided differential scale perturbation in $\textrm{STPO}$, thereby strengthening the algorithm’s global exploration capability.

In the implementation of STPO, this paper further introduces a worst-guided differential scale perturbation strategy. Let the worst fitness in the current population be $f_{\max }^{t}=\max _{i} f_i^{t}$, with the corresponding position $X_{\textrm{worst}}^{t}$. When an individual is in the non-alert branch of the stranger-avoidance behavior, it performs the update shown in Eq. (12) with probability *p=0.5*. This strategy leverages the positional information between an individual and the worst solution, and adjusts the step size based on fitness differences, thereby enhancing the ability to escape local optima with a certain probability while improving exploration.

12 $$\begin{aligned} X_i^{t+1}=X_i^{t}+\left( 2u_i^{t}-1\right) \cdot \frac{\left| X_i^{t}-X_{\textrm{worst}}^{t}\right| }{f_i^{t}-f_{\max }^{t}+\varepsilon },\quad u_i^{t}\sim U(0,1) \end{aligned}$$

Eq. (12) gives the worst-guided differential scale perturbation update introduced in STPO^37^, where $u_i^{t}$ is a random perturbation factor drawn from the uniform distribution *U(0,1)*, and $\left| X_i^{t}-X_{\textrm{worst}}^{t}\right|$ denotes the element-wise absolute positional difference between an individual and the worst individual in the population. $f_i^{t}$ is the fitness of the individual, $f_{\max }^{t}$ is the worst fitness in the population, and *\varepsilon* is a very small constant used to avoid a zero denominator. This mechanism adjusts the step size according to fitness differences and regulates the update magnitude using the random perturbation factor, guiding individuals to escape local optima through information interaction with the worst solution and enhancing the global search capability of the algorithm.

### Flexible application of the levy perturbation

Perturbation operators play an important role in regulating the transition between exploration and exploitation in swarm intelligence algorithms^38^. In the original PO algorithm, $\textrm{Levy}$ perturbation is mainly associated with the foraging behavior, where it helps individuals perform long-distance jumps and escape local optimum regions. In contrast, STPO introduces different perturbation forms into different update paths:

- In the dominant-individual updates of the predation and resting behaviors, $\textrm{Levy}$ flight is introduced to strengthen cross-region jumping capability.
- In the alertness update, a Gaussian random vector perturbation is used to re-locate disadvantaged individuals toward the elite neighborhood, thereby accelerating aggregation toward favorable regions.
- In the stranger-avoidance behavior, a trigonometric modulation term is retained to maintain a certain level of exploitation, and the worst-guided differential scale perturbation is introduced with a certain probability to enhance exploration.

Through the cooperative design of these multi-form perturbations, STPO facilitates a clear transition from early-stage exploration to later-stage exploitation, thereby improving convergence efficiency while retaining a certain degree of adaptive search capability on complex landscapes.

**Fig. 3:**
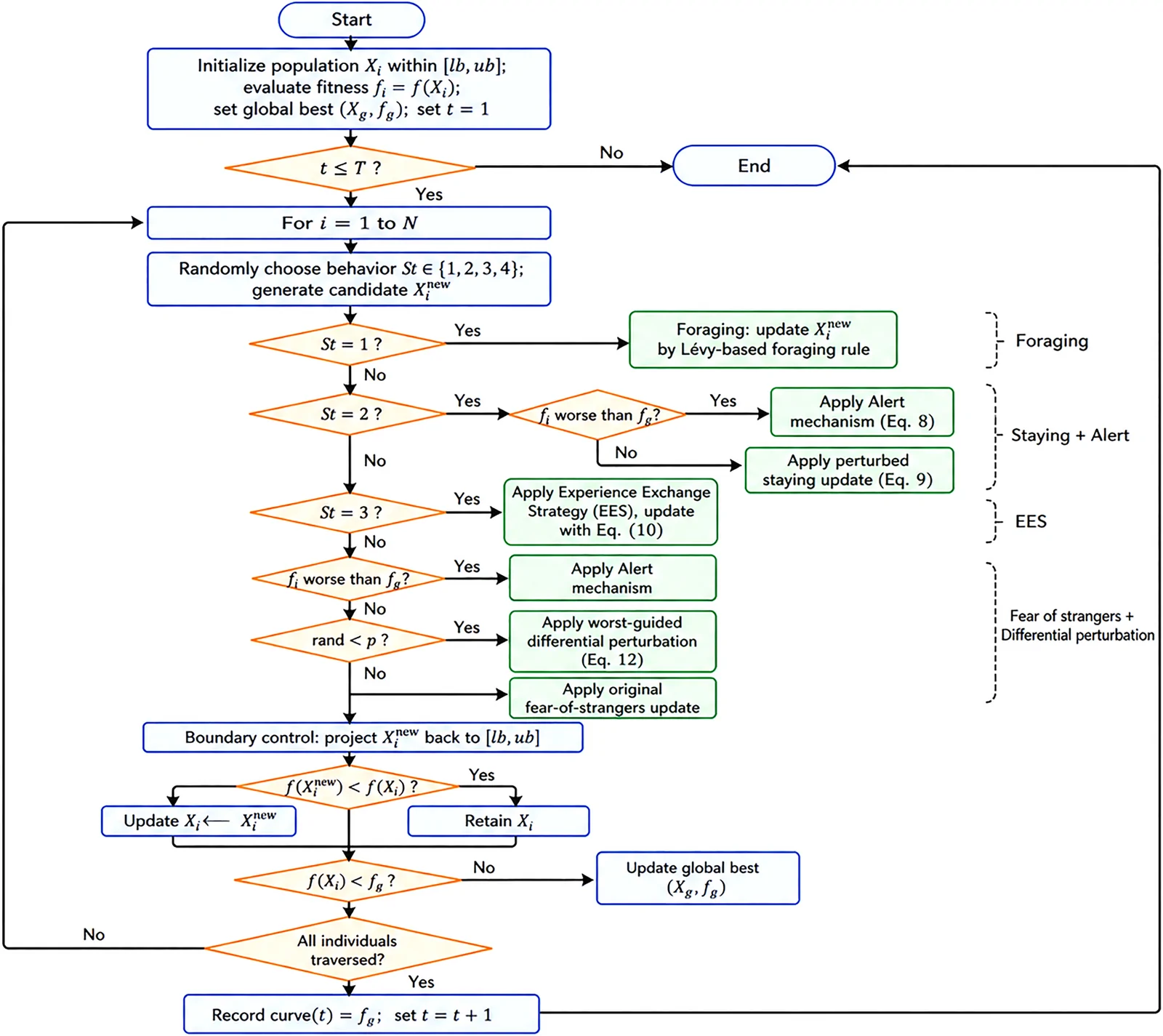
Flow chart of the STPO algorithm.

Figure 3 illustrates the STPO workflow and how the improvement strategies are applied. After population initialization and fitness evaluation, each individual randomly selects a behavior from PO’s four options. For staying (St = 2) or fear-of-strangers (St = 4) behaviors, the alert protection mechanism (APM) helps disadvantaged individuals escape low-quality regions. When the communication behavior (St = 3) is selected, the experience exchange strategy (EES) promotes information sharing among individuals. In the fear-of-strangers branch, if the solution is worse than the global best, a probability-based worst-guided differential scale perturbation (DSP) is applied to enhance global exploration. After each candidate solution is generated, boundary control is performed, followed by greedy selection and global best update.

### Computational complexity analysis

Computational complexity serves as a crucial metric for evaluating the performance of optimization algorithms^39^. Through time complexity analysis, one can assess an algorithm’s operational efficiency across problems of varying scales. Below, we analyze and compare the computational complexities of the PO algorithm and the STPO algorithm proposed in this paper.

Assume a population size of *N*, problem dimension *D*, and a maximum of *T* iterations. In the original paper, the time complexity of PO is described as follows: initialization costs *O(N)*; fitness evaluation costs $O(T\times N\times D)$ because all particles are evaluated each iteration and the evaluation depends on *D*; and the update stage (position update and best-solution search) costs $O(T\times N)+O(T\times N\times D)+O(T\times N\times \log N)$, where *O(T× N)* accounts for behavioral updates, $O(T\times N\times D)$ for fitness evaluation and position updates, and $O(T\times N\times \log N)$ for sorting fitness values. In complexity analysis, if *T* is independent of *N* or grows much more slowly than the dominant terms, then *O(T× N)* is typically treated as a constant-factor or lower-order term and absorbed into the leading terms. Therefore, the overall time complexity is $O\!\left( T\times (N\times D + N\times \log N)\right)$.

In comparison, the time complexity of STPO is lower. The initialization complexity of STPO is *O(N× D)*, and the fitness evaluation complexity is *O(N× D)*. During solution updating, the per-particle update cost is *O(D)*; since no sorting operation is required, the overall update complexity is *O(N× D)*. Therefore, the total time complexity of STPO is $O(T\times N\times D)$. In most cases, STPO has a lower time complexity than PO, especially when the number of particles is large.

Additionally, Table 1 presents the time complexity results for several common algorithms. Clearly, the time complexity of our proposed STPO algorithm is largely consistent with these algorithms, outperforming the PO algorithm and its variants.

The computational complexity analysis indicates that the proposed STPO algorithm maintains the same asymptotic complexity order as the original PO algorithm. Although STPO introduces additional strategies, including the sparrow alertness mechanism, experience exchange, and differential scale perturbation, these operators mainly introduce limited constant-level computational overhead and do not change the dominant complexity term. Specifically, the sparrow alertness mechanism helps guide disadvantaged individuals away from low-quality regions, the experience exchange strategy enhances information interaction within the population, and the differential scale perturbation improves the exploration capability of the solution space. To further examine the practical computational cost, the actual running time of STPO and the baseline algorithms was compared on a unified computing platform. According to the CEC2017 test results with 30 and 50 dimensions reported in Tables 24 and 25 of Appendix B, STPO achieves shorter execution time than PO in the tested cases. These empirical results are consistent with the theoretical complexity analysis and indicate that the proposed strategies can improve optimization performance without introducing significant additional computational burden.

**Table 1:** Computational complexity of several common algorithms.

**Algorithm**	**Complexity**
GA	$O(T\times N\times D)$
PSO	$O(T\times N\times D)$
POA	$O(T\times N\times D)$
AO	$O(T\times N\times D)$
PO	$O\!\bigl (T\times (N\times D + N\times \log N)\bigr )$
MIPO	$O(T\times N\times D)$
CGBPO	$O\!\bigl (T\times (N\times D + N\times \log N)\bigr )$
STPO	$O(T\times N\times D)$

Algorithm 1 presents the pseudocode for STPO.

**Algorithm 1:**
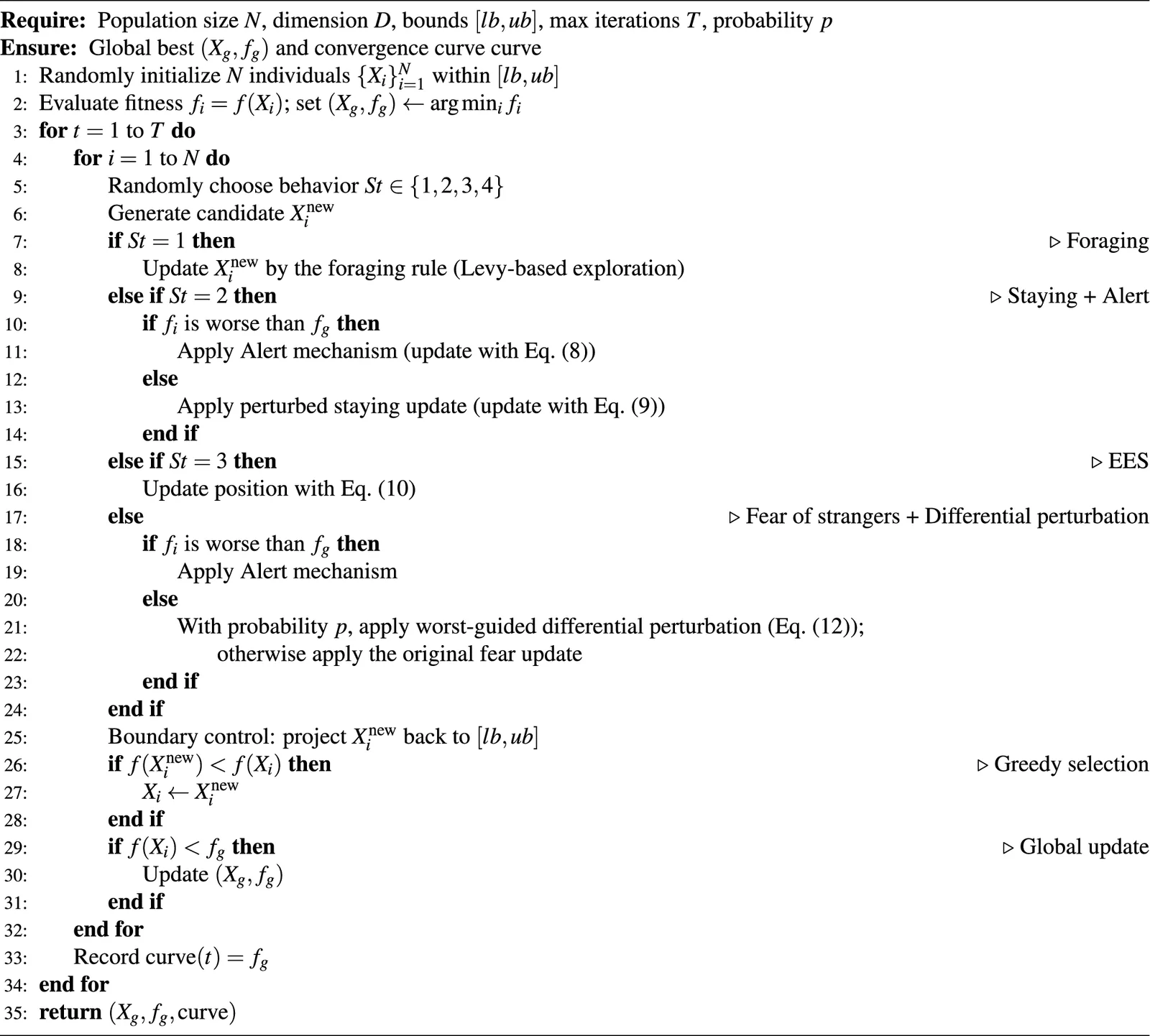
Pseudo-code of the STPO algorithm.

## Numerical experiments on CEC benchmarks

In this section, we conduct a series of numerical experiments on the CEC benchmark test suites to systematically and comprehensively evaluate the overall performance of STPO.

To begin with, Section 3.1 establishes the fundamental experimental framework, including details on the computational platform, software and hardware environments, evaluation metrics, and runtime configurations. Section 3.2 outlines the CEC benchmark tasks used and details the key configurations for each algorithm employed in the comparison. Subsequently, Section 3.3 summarizes and analyzes the experimental results for the CEC2017 and CEC2022 test functions from multiple perspectives, including convergence behavior, stability, statistical significance, and percentage improvement over PO-family optimizers. Section 3.4 presents the ablation study to validate the effectiveness of the proposed key strategies and identify the sources of STPO’s performance improvement. Section 3.5 further investigates the population diversity and exploration–exploitation behavior of STPO, providing additional insight into how the proposed optimizer dynamically transfers from global exploration to local exploitation during the search process.

### Experimental setup and environment

These experiments were conducted on a system with the following configuration:

- Operating system: Windows 11
- Device name: LAPTOP-PE1C6HJH
- Processor: 13th Gen Intel(R) Core(TM) i7-13650HX 2.60 GHz
- Memory: 16 GB RAM
- Programming environment: MATLAB R2025a

To ensure the high reliability of experimental results, we strictly maintain consistent hardware and software environments for all experiments. This approach minimizes potential biases from any hardware or software variations, thereby guaranteeing the fairness and objectivity of optimization algorithm testing.

### Comparison algorithm and parameter settings

We selected the CEC2017 and CEC2022 benchmark test suites to comprehensively evaluate the overall performance of the proposed STPO algorithm, as they cover a variety of complex, multimodal, and high-dimensional optimization problems. In addition, we selected 12 representative optimizers from different categories as competitors to assess the competitiveness of STPO. These competitors can be grouped into four categories:

- Traditional metaheuristic algorithms: Genetic Algorithm (GA) and Particle Swarm Optimization (PSO).
- Frequently cited algorithms: Harris Hawks Optimization (HHO)^40^ and Whale Optimization Algorithm (WOA)^41^.
- Recently proposed metaheuristic algorithms: Dung Beetle Optimization (DBO)^42^, Pelican Optimization Algorithm (POA)^43^, Aquila Optimizer (AO)^44^, Tetragonula Gressitti Colony Optimization Algorithm (TGCOA)^45^, and Zebra Optimization Algorithm (ZOA)^46^.
- Variants of the PO algorithm: PO, MIPO, and CGBPO.

Table 2 summarizes the parameter settings of all competing algorithms. In the experiments, the population size was set to 100, the maximum number of iterations was set to 1000, and each algorithm was run 30 times on each test function.The same boundary-handling strategy and stopping criterion were used for all algorithms. For each compared optimizer, the main control parameters were set according to the corresponding original papers or commonly used recommended settings. For algorithms without explicit user-defined parameters, their default update rules were retained.No additional problem-dependent parameter tuning was performed for STPO or any competing optimizer, so that all algorithms were evaluated under a unified and reproducible experimental configuration.

**Table 2:** Main control parameters for all comparison algorithms.

**Mas**	**Parameters**	**Value**
GA	Probability of crossover $P_c$	0.6
	Probability of mutation $P_m$	0.05
	Inertia factor *ω*	1
PSO	Acceleration coefficients $c_1$ and $c_2$	2.05
	$Speed_{\max }$ and $Speed_{\min }$	*2, -2*
HHO	No Parameters	
WOA	No Parameters	
DBO	Proportion of producers $P_{\textit{percent}}$	0.2
POA	Constant during update $X_{\textit{new}}$	0.2
AO	Constant for position update $\alpha ,\ \delta$	*0.1,\ 0.1*
	Constant for the calculation of angle *ϕ* *ω*	0.005
TGCOA	Helical motion parameters *a,\ b*	*0,\ 10*
	Temperature generation constant *k*	0.03
ZOA	Attack intensity constant *R*	0.1
PO	No Parameters	
MIPO	No Parameters	
CGBPO	Control parameters of chaotic logical mapping *a*	4
STPO	Perturbation probability *p*	0.5
	Levy stability index *γ*	1.5

### Comparative experiment and analysis of CEC2017 and CEC2022

To evaluate the performance differences between the STPO algorithm and other competitive algorithms, this paper employs the Mann–Whitney U test to analyze the experimental results^47^. The test results are shown using the symbols ‘+’, ‘*∼*’ and ‘−’, where ‘+’ indicates that STPO is superior to the comparison algorithm, ‘*∼*’ denotes no statistical significance, and ‘−’ signifies that STPO is statistically significantly worse than the other algorithm. The Friedman rank test was employed to comprehensively evaluate the average performance rankings of each algorithm across different test sets, with the algorithm achieving the optimal average rank highlighted in bold^48^. Tables 3 and 4 summarize the statistical significance and average rankings of algorithms in the CEC2017 and CEC2022 test sets, respectively, with detailed results presented in Appendix B.

To further quantify the performance improvement of STPO over PO-family optimizers, the percentage improvement is calculated with respect to the original PO and its representative variants. In this study, the PO-family baseline optimizers include PO, MIPO, and CGBPO. For a minimization problem, the improvement ratio of STPO over a baseline optimizer is defined as:

13 $$\begin{aligned} \textrm{Improvement}_{b}(\%)= \frac{\textrm{Mean}_{b}-\textrm{Mean}_{\textrm{STPO}}}{|\textrm{Mean}_{b}|+\epsilon } \times 100\%, \end{aligned}$$

where $b \in \{\textrm{PO}, \textrm{MIPO}, \textrm{CGBPO}\}$ denotes a PO-family baseline optimizer, $\textrm{Mean}_{b}$ and $\textrm{Mean}_{\textrm{STPO}}$ represent the average objective values obtained by the baseline optimizer and STPO, respectively, and $\epsilon =10^{-12}$ is a small constant used to avoid division by zero. A positive improvement value indicates that STPO obtains a smaller mean objective value than the corresponding baseline optimizer. Since different CEC functions have different numerical scales, the improvement ratio is first calculated function by function and then averaged over different dimensions and test functions.

**Fig. 4:**
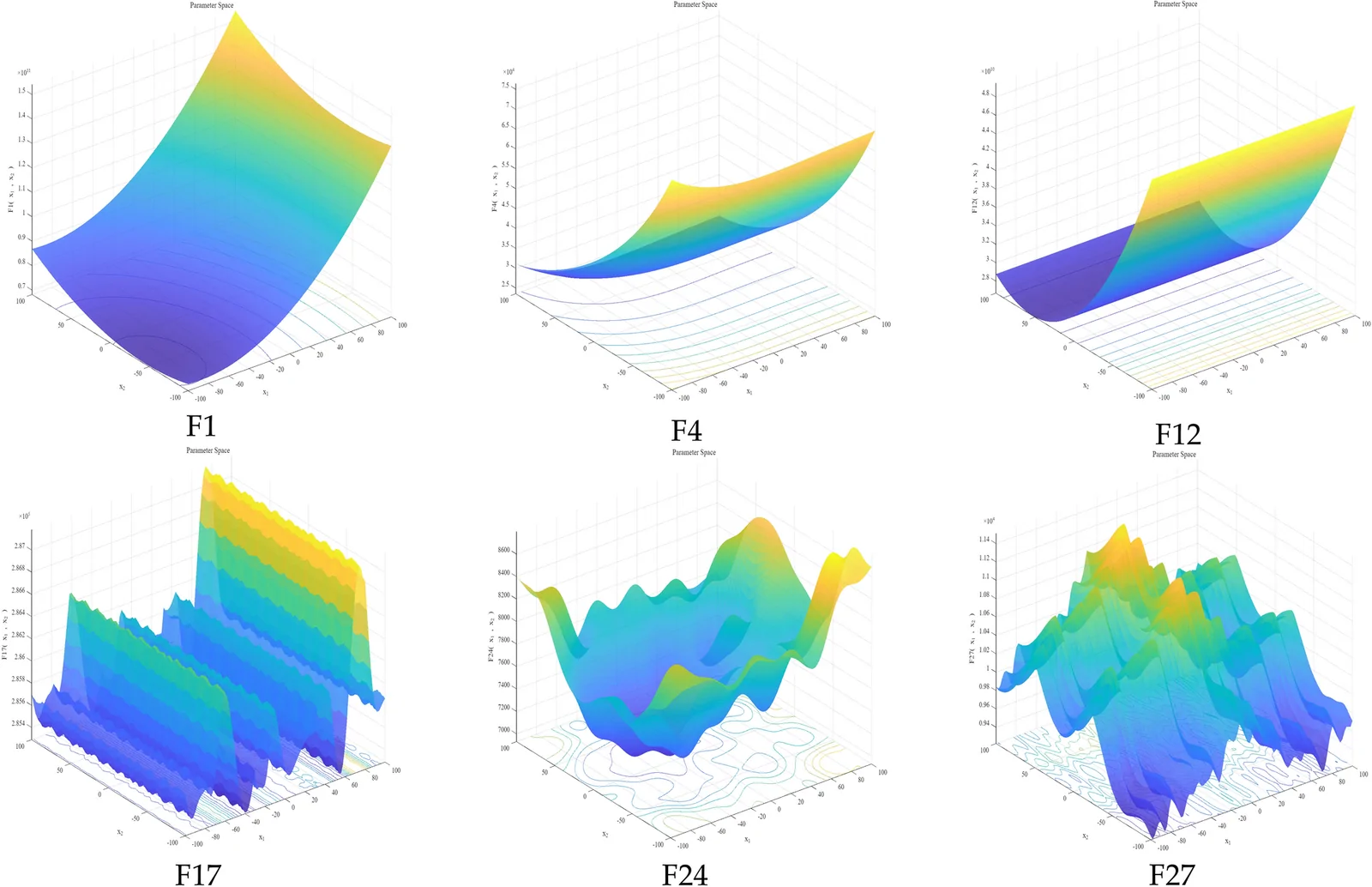
3D surface visualization of representative functions in the CEC2017 test suite.

**Fig. 5:**
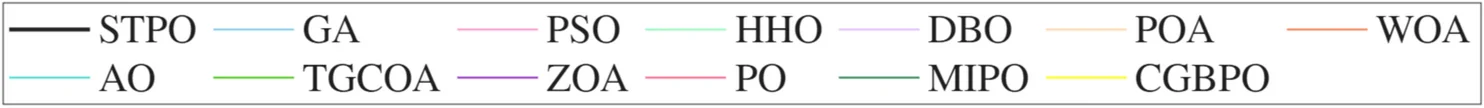
The assigned colors for each algorithm in the CEC numerical experiments.

**Fig. 6:**
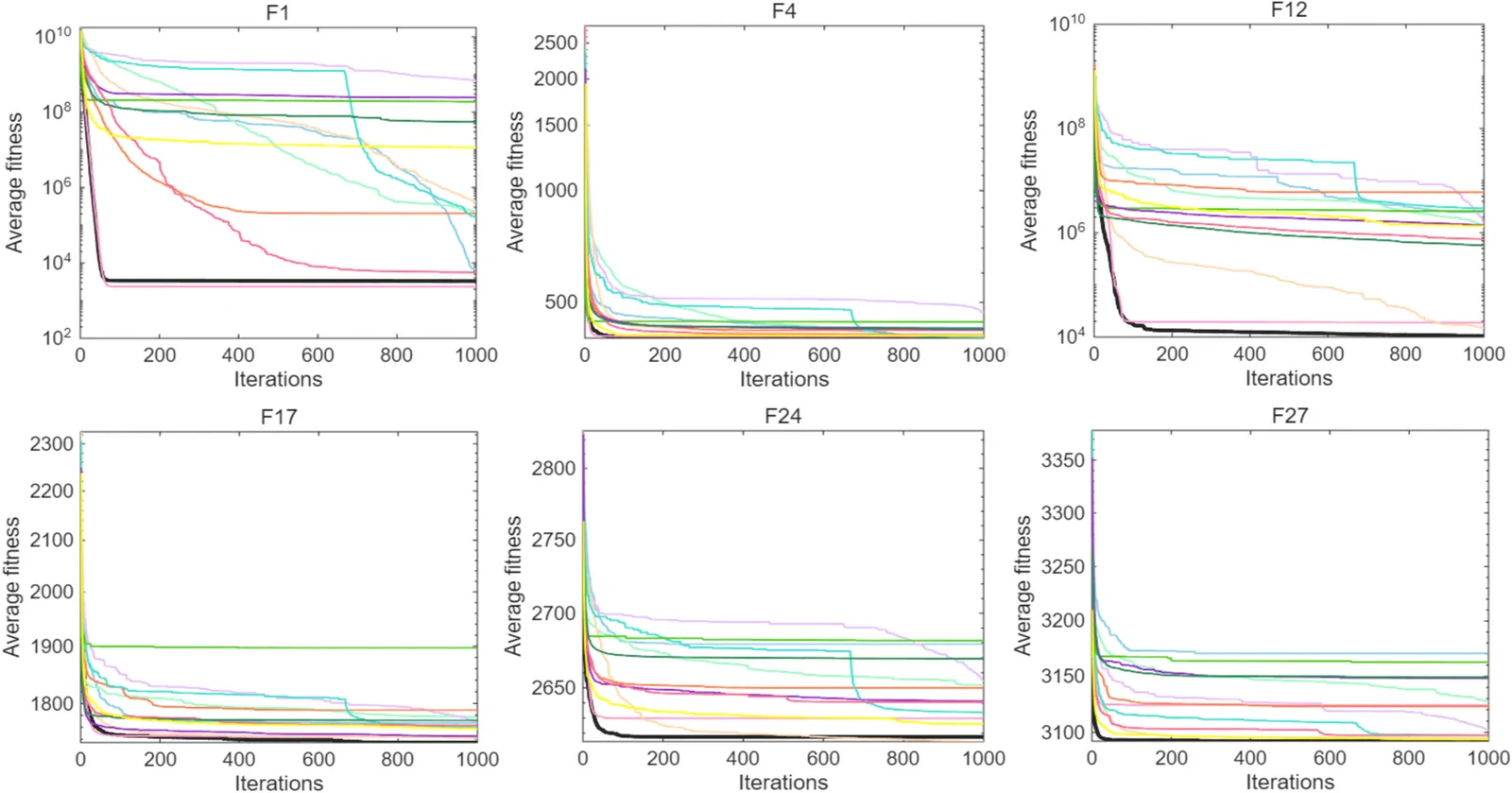
Convergence curves of CEC2017 benchmark functions in 10 dimensions.

**Fig. 7:**
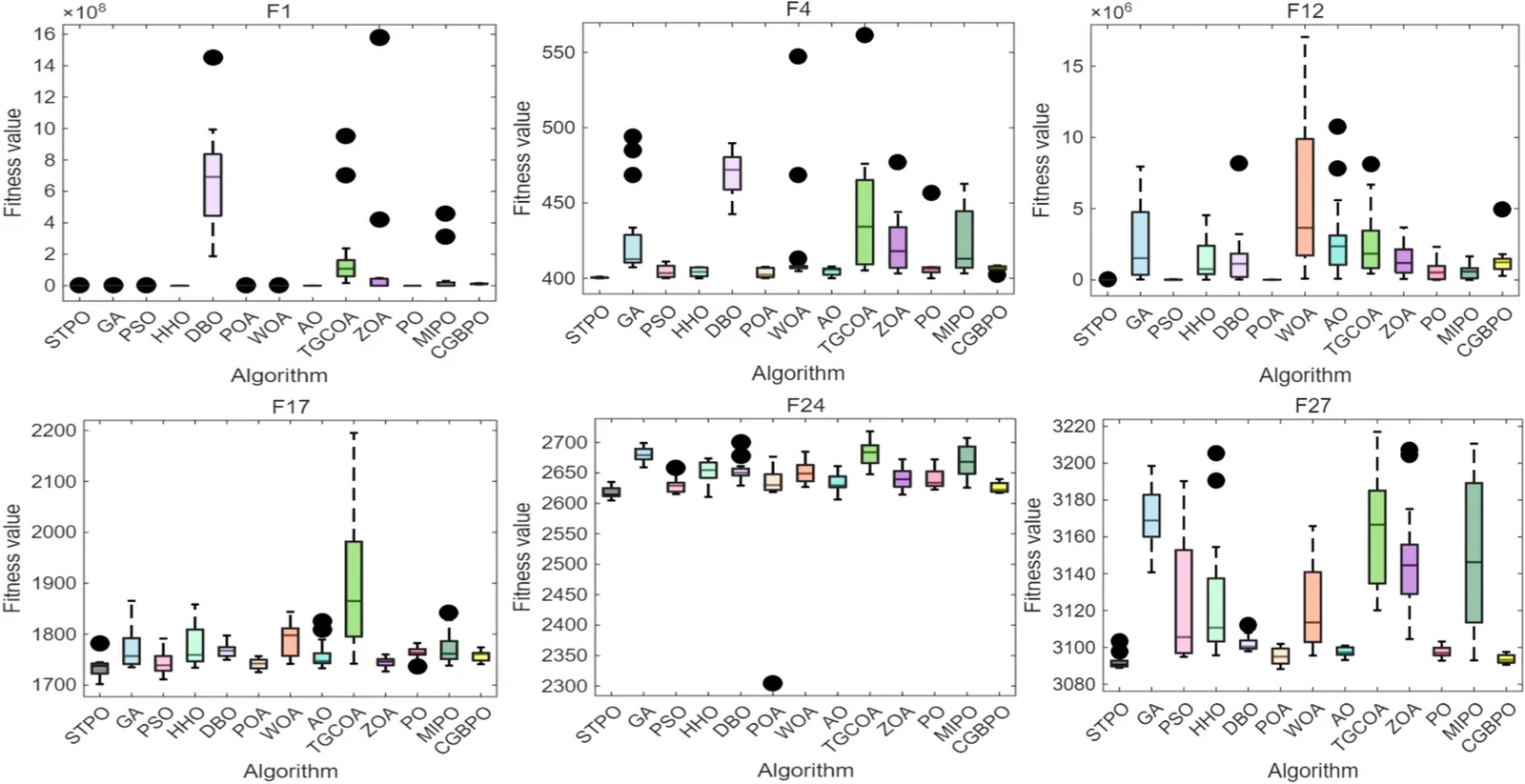
Box plots of CEC2017 benchmark functions in 10 dimensions.

**Fig. 8:**
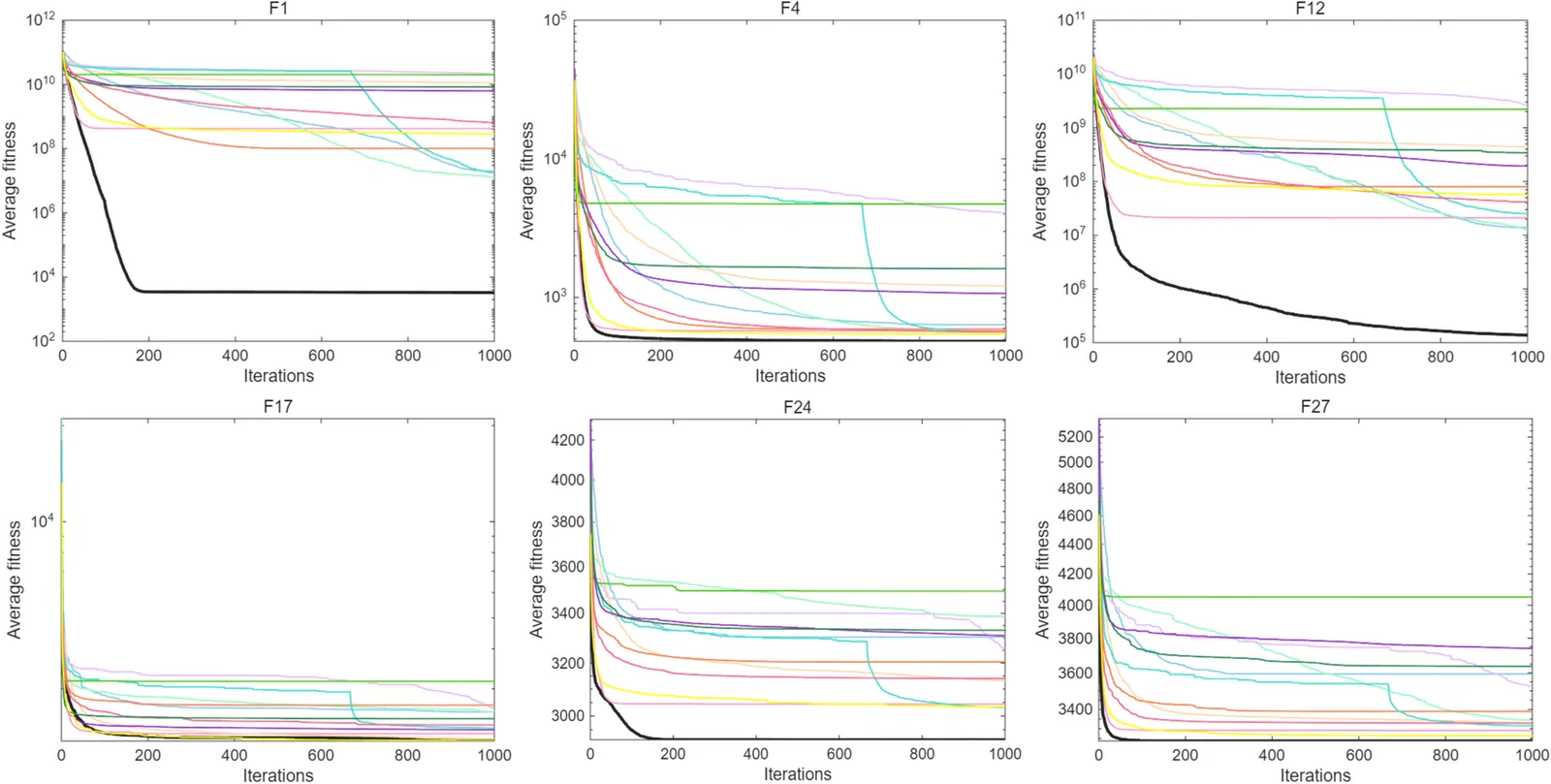
Convergence curves of CEC2017 benchmark functions in 30 dimensions.

**Fig. 9:**
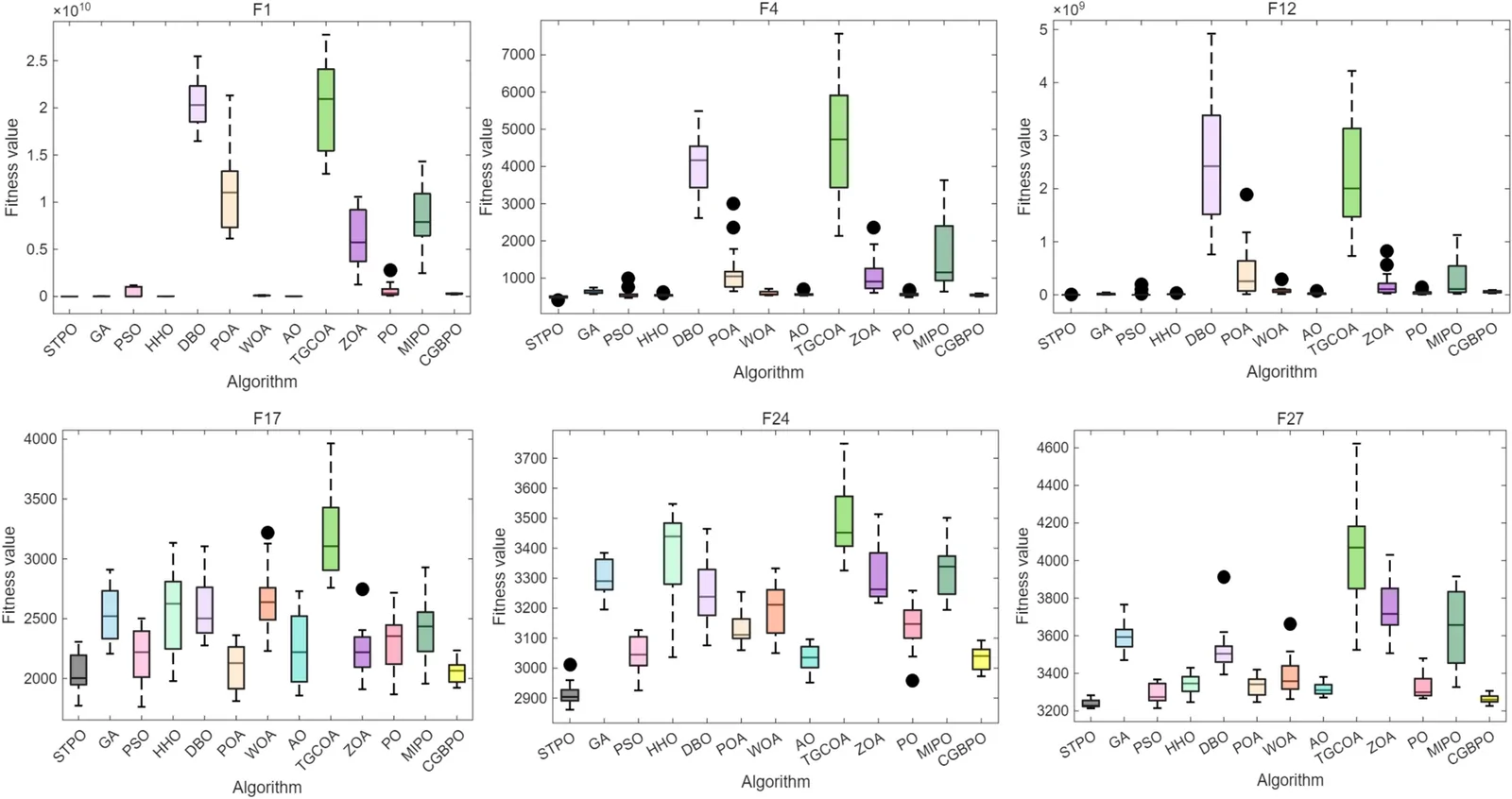
Box plots of CEC2017 benchmark functions in 30 dimensions.

**Fig. 10:**
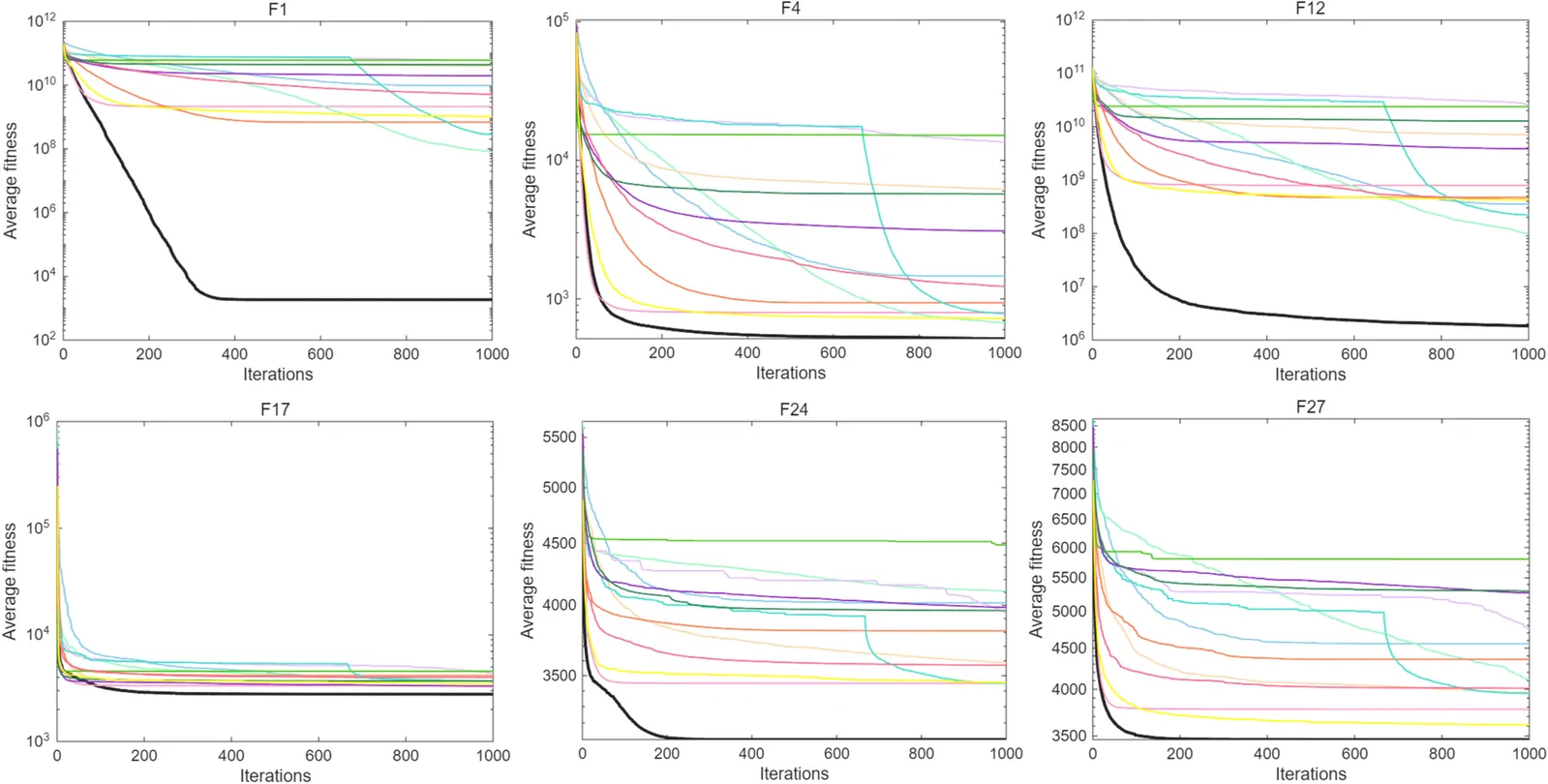
Convergence curves of CEC2017 benchmark functions in 50 dimensions.

**Fig. 11:**
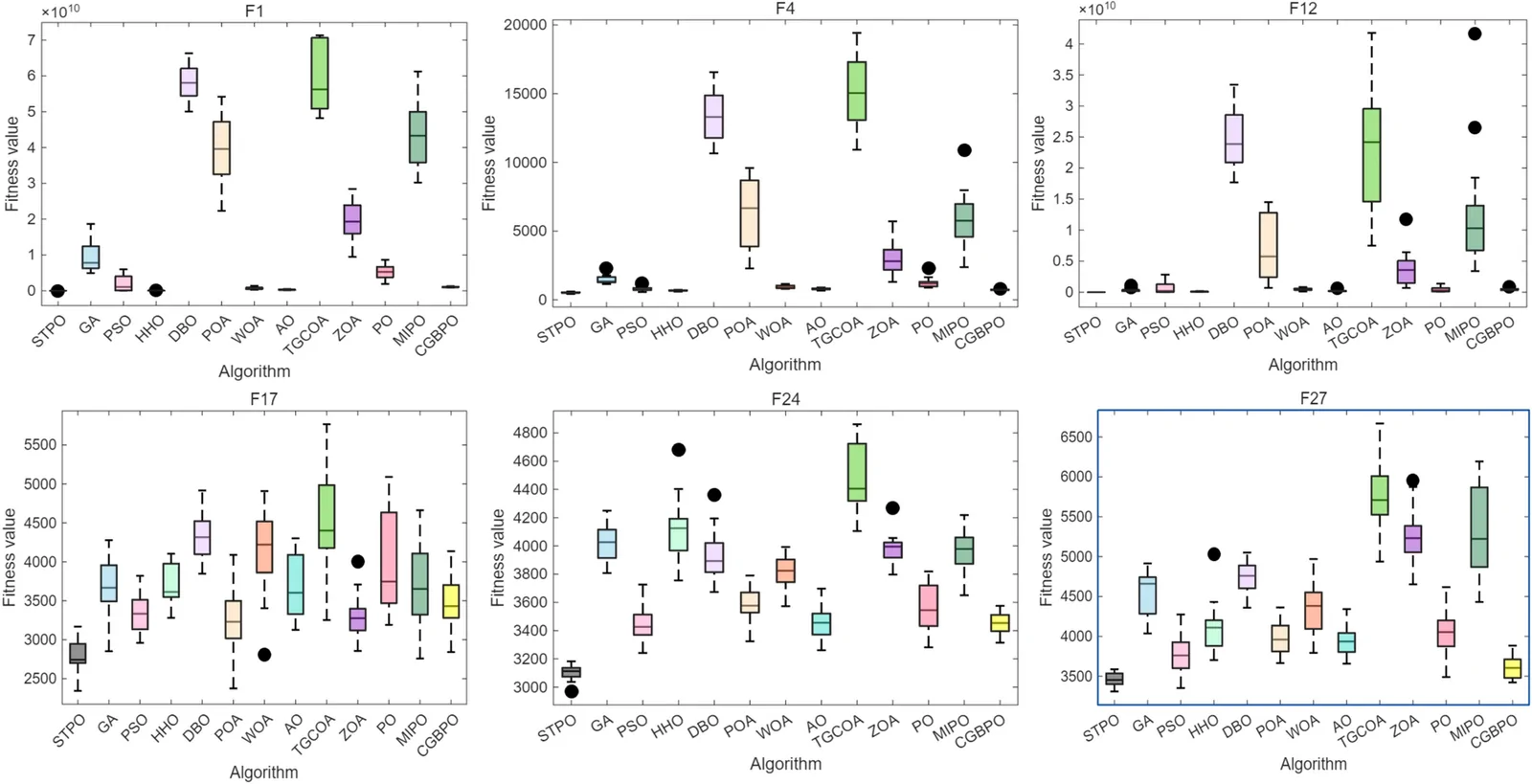
Box plots of CEC2017 benchmark functions in 50 dimensions.

**Fig. 12:**
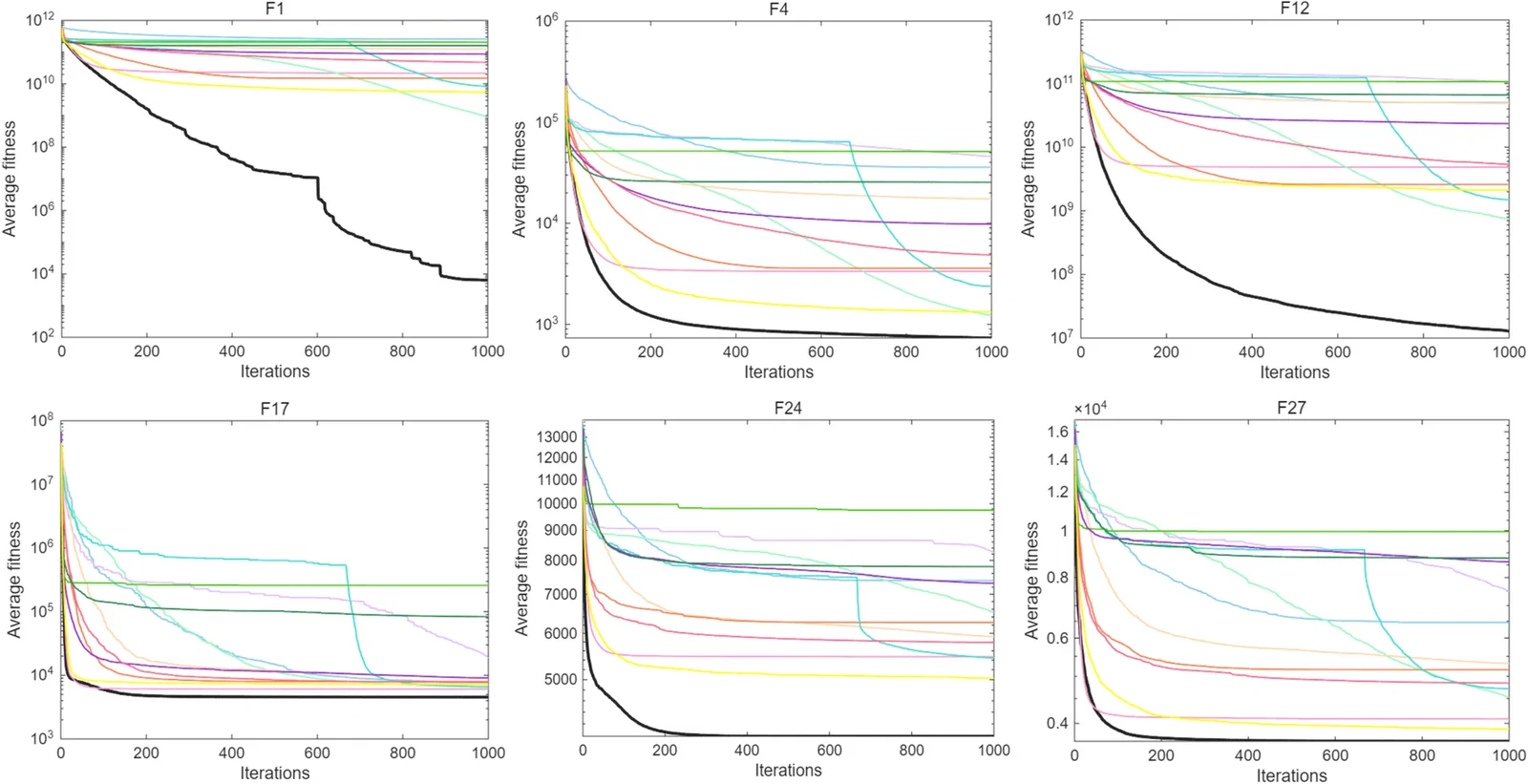
Convergence curves of CEC2017 benchmark functions in 100 dimensions.

**Fig. 13:**
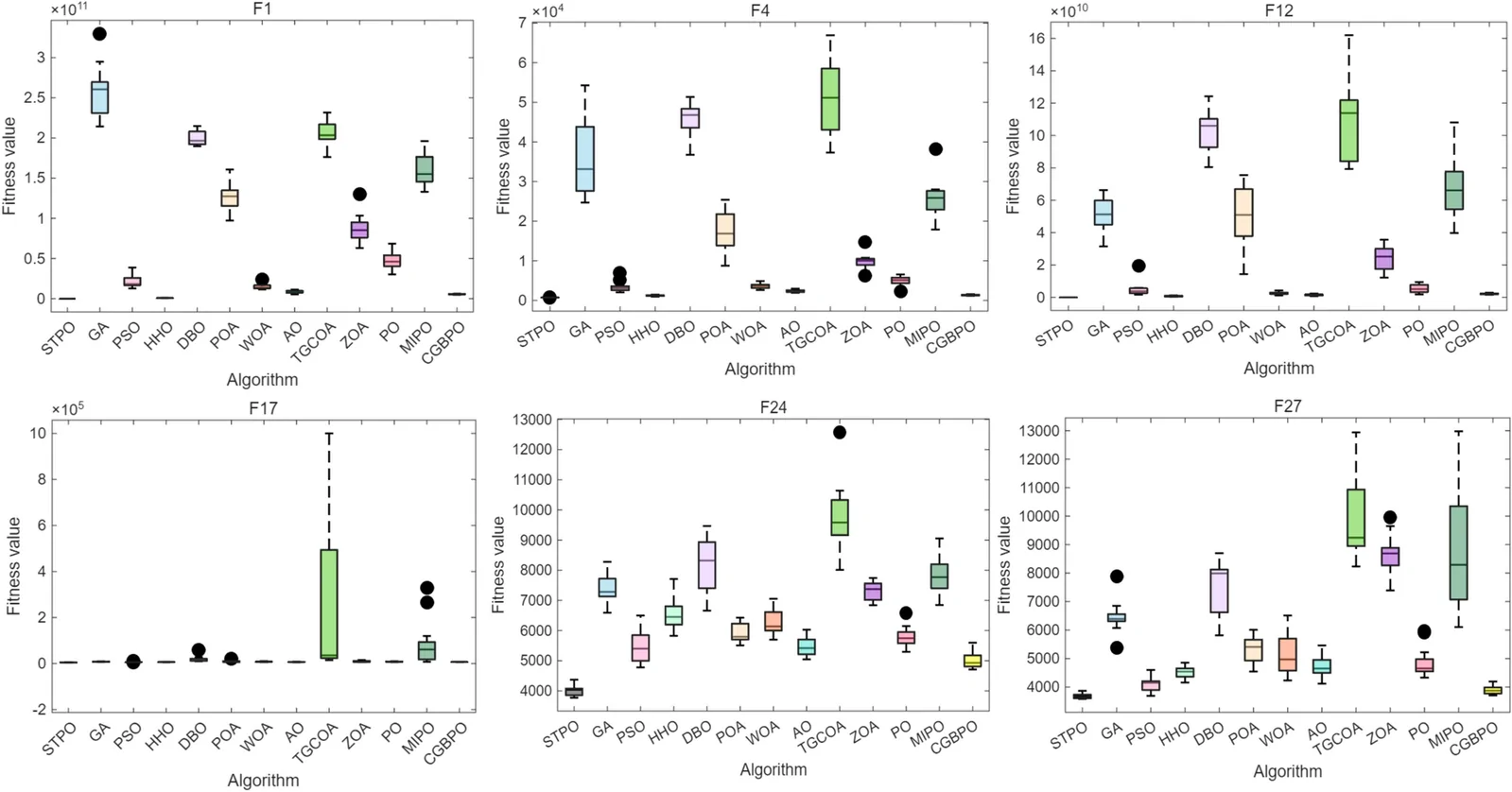
Box plots of CEC2017 benchmark functions in 100 dimensions.

**Fig. 14:**
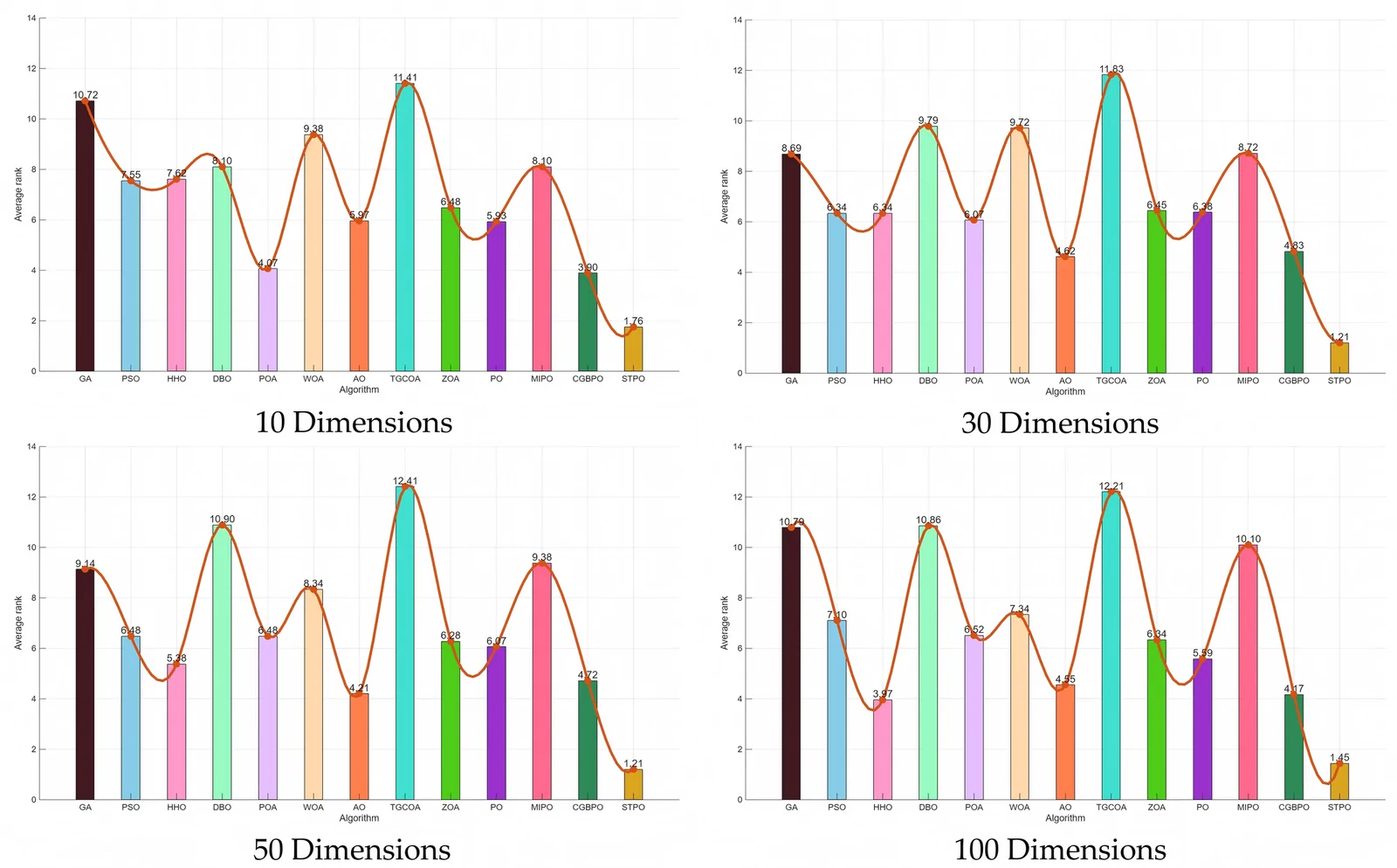
Average ranking plots of algorithms in CEC2017.

**Fig. 15:**
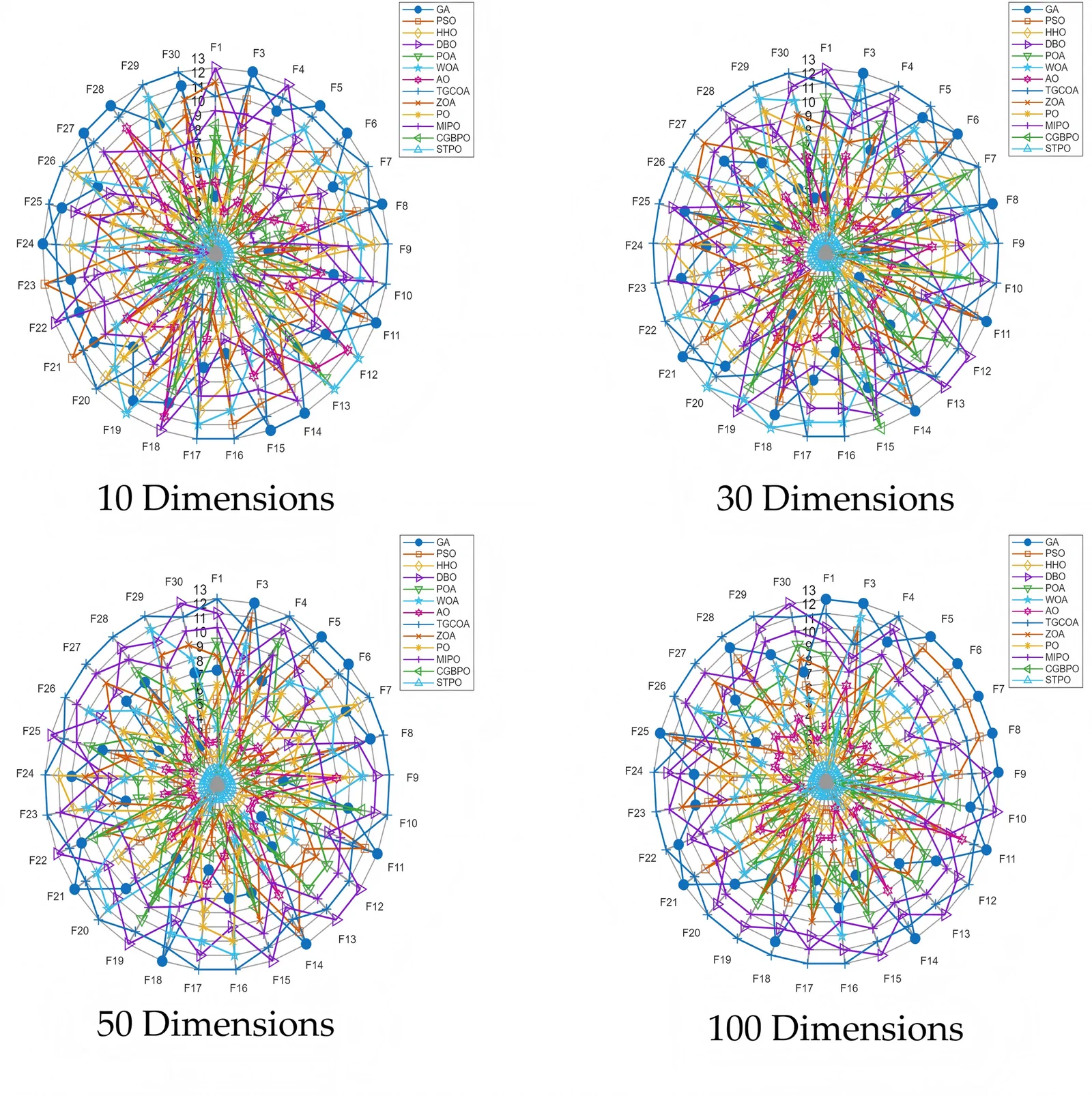
Radar charts of algorithms for each dimension in CEC2017.

**Fig. 16:**
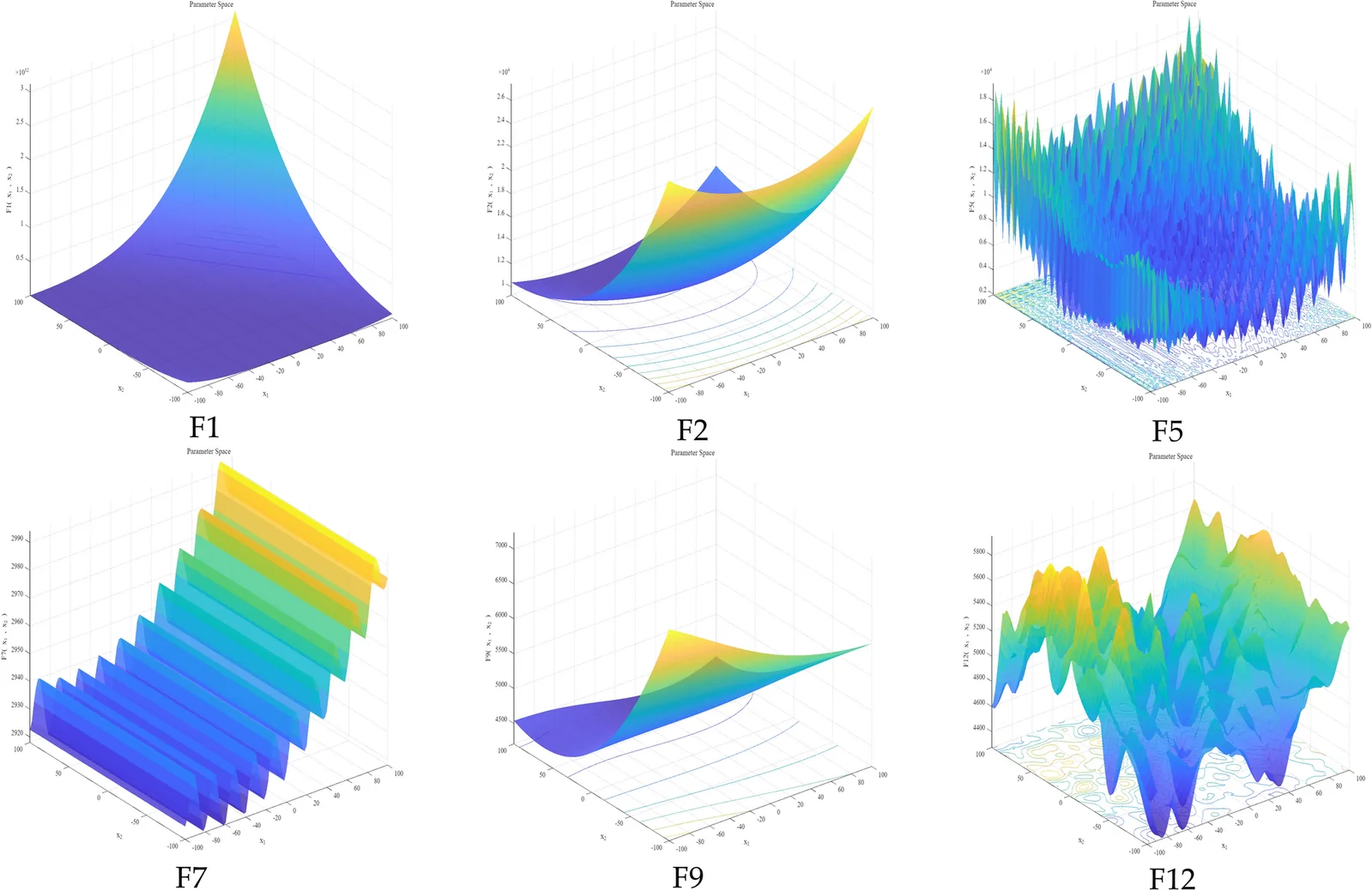
3D surface visualization of representative functions in the CEC2022 test suite.

**Fig. 17:**
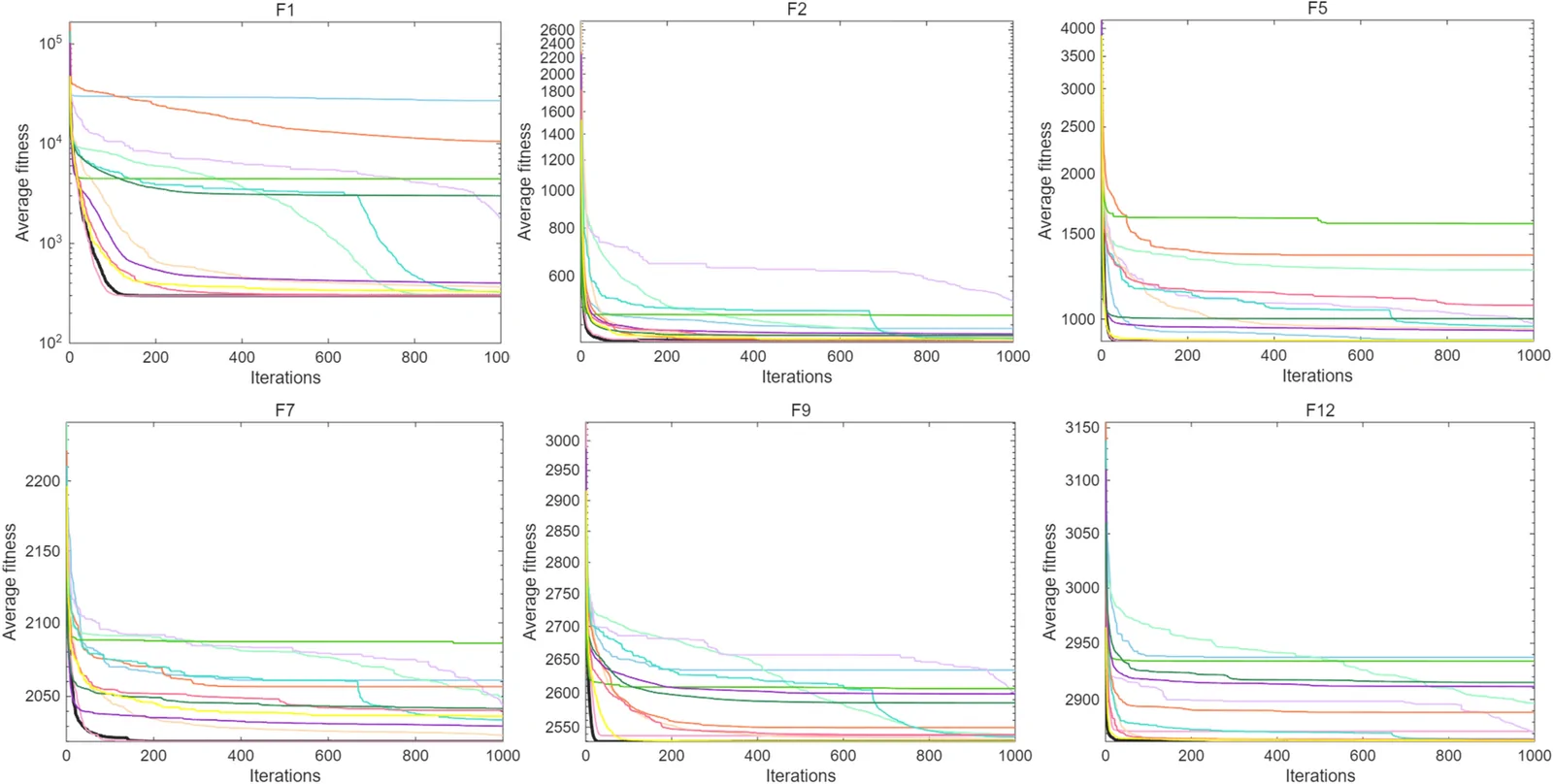
Convergence curves of CEC2022 benchmark functions in 10 dimensions.

**Fig. 18:**
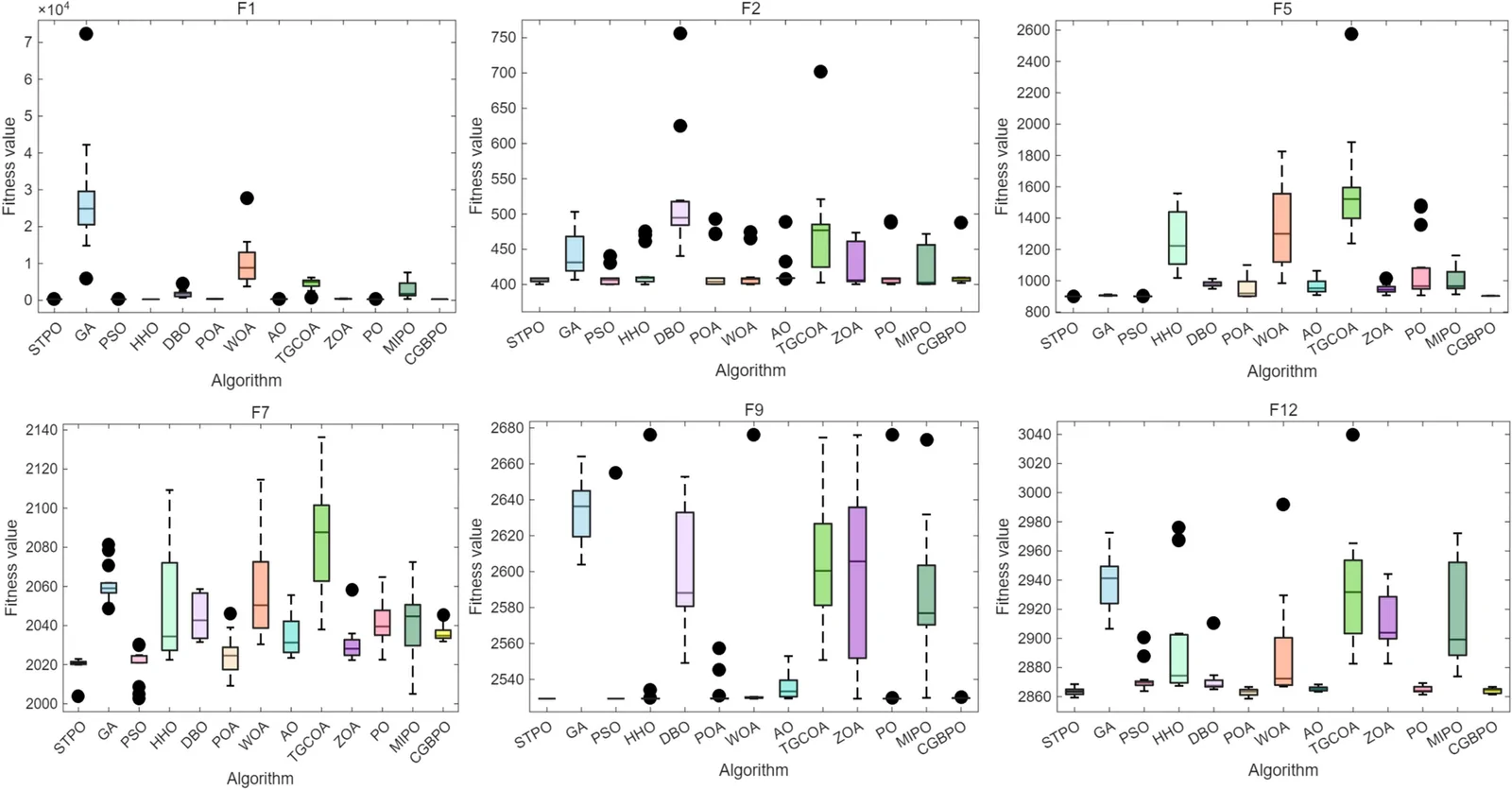
Box plots of CEC2022 benchmark functions in 10 dimensions.

**Fig. 19:**
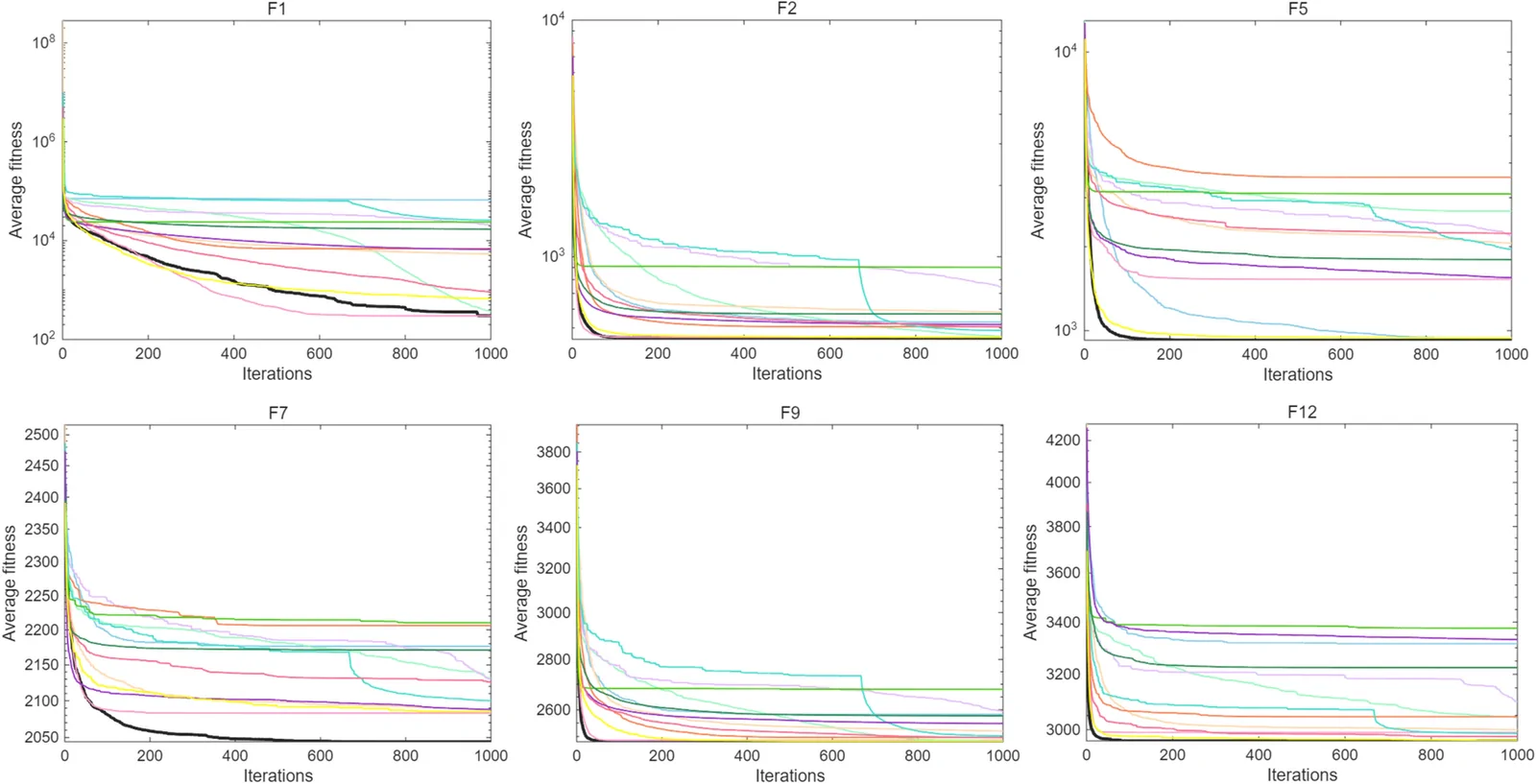
Convergence curves of CEC2022 benchmark functions in 20 dimensions.

**Fig. 20:**
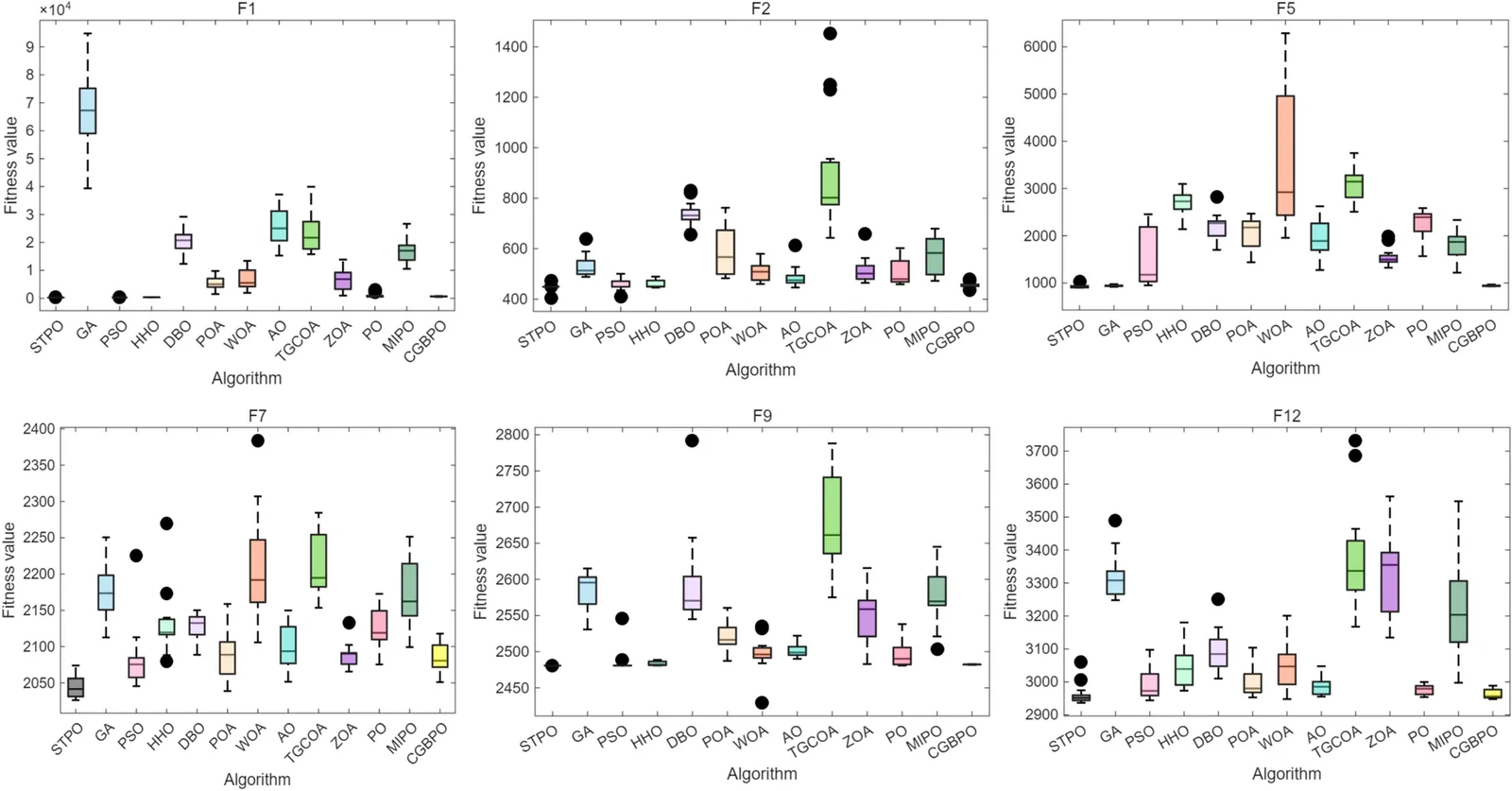
Box plots of CEC2022 benchmark functions in 20 dimensions.

**Fig. 21:**
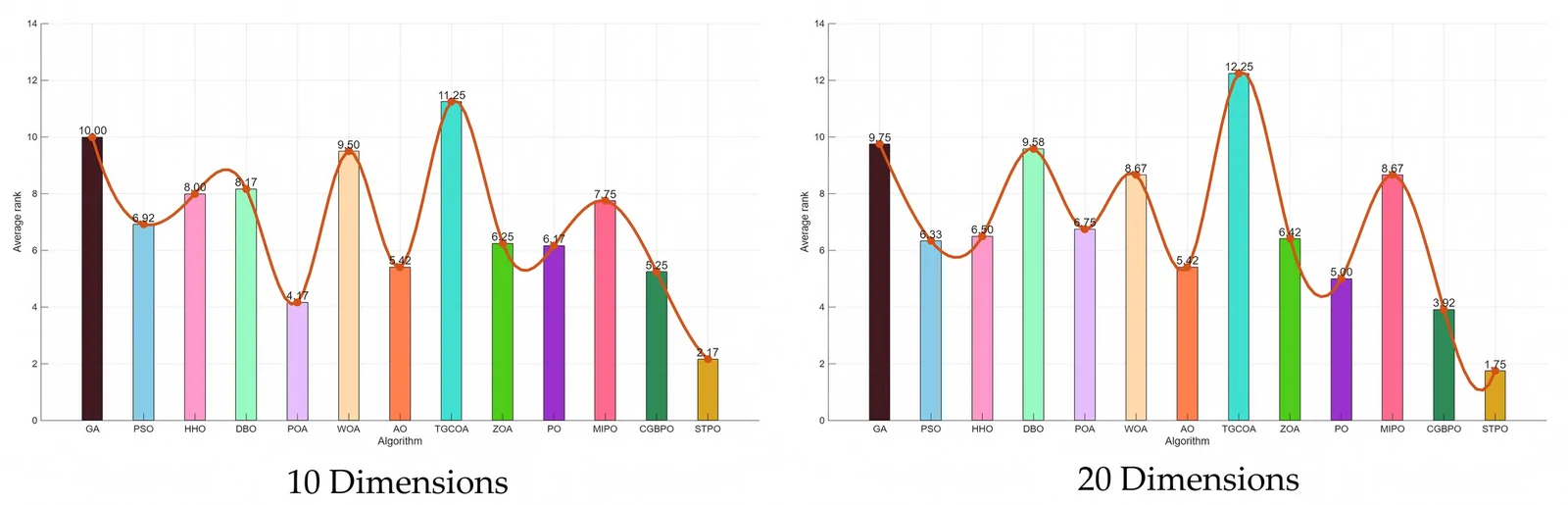
Average ranking plots of algorithms for each dimension in 2022.

**Fig. 22:**
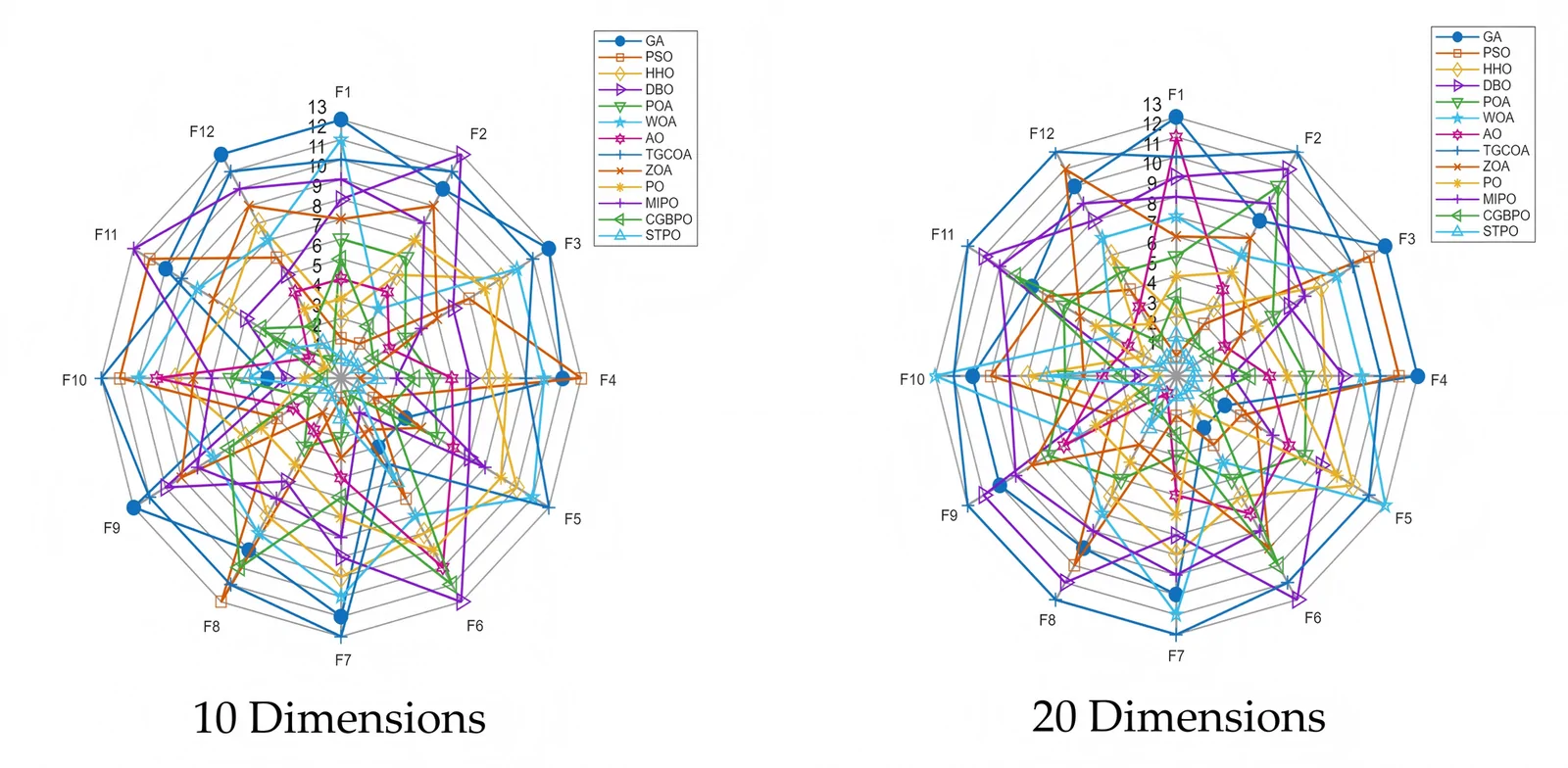
Radar charts of algorithms for each dimension in 2022.

We also ranked algorithms based on their average fitness scores, comparing standard deviations when means were equal. Ties occurred when both means and standard deviations were identical. Figures 4 and 16 present three-dimensional visualization plots of representative functions from the CEC2017 and CEC2022 test sets, respectively. Figure 5 show the convergence curves and box plots for each algorithm in the CEC2017 and CEC2022 test sets, respectively. Figures 6, 7, 8, 9, 10, 11, 12, 13 display convergence curves and box plots for optimizers on representative functions in CEC2017 (F1: unimodal function; F4: multimodal function; F12 and F17: mixed functions; and F24 and F27: composite functions). Radar charts provide a more intuitive representation of the ranking results of various algorithms across different test functions. A smaller enclosed area indicates a higher algorithm ranking. Average ranking plots offer a clear visualization of each algorithm’s performance^49^. Figures 14 and 15 present the average ranking plot and radar chart for CEC2017 algorithms, respectively. Figures 17, 18, 19, 20 display the convergence curves and box plots of optimizers for representative functions in CEC2022 (F1: unimodal function; F2 and F5: basic functions; F7 and F9: hybrid functions; and F12: composite function). Figures 21 and 22 present the average ranking plots and radar charts for each algorithm in CEC2022, respectively.

Furthermore, statistical results on the average runtime of each algorithm across the CEC2017 test set under 30-dimensional and 50-dimensional settings demonstrate that the proposed STPO exhibits significantly shorter computational time compared to the original PO. This indicates that the method achieves higher computational efficiency and engineering applicability while maintaining or enhancing solution performance. Detailed comparative runtime data are provided in Appendix B.

**Table 3:** Summarized experimental results of comparison experiments in CEC2017.

**MAs**	10Dim		30Dim		50Dim		100Dim	
	+/*≈*/-	Avg. Ranks	+/*≈*/-	Avg. Ranks	+/*≈*/-	Avg. Ranks	+/*≈*/-	Avg. Ranks
GA	27/3/0	10.72	29/1/0	8.68	30/0/0	9.14	30/0/0	10.79
PSO	25/5/0	7.55	24/5/1	6.43	24/6/0	6.48	27/3/0	7.10
HHO	25/5/0	7.62	29/1/0	6.21	29/0/1	5.38	26/3/1	3.97
DBO	25/4/1	8.10	29/1/0	9.75	30/0/0	10.90	30/0/0	10.86
POA	23/7/0	4.07	25/4/1	6.17	26/4/0	6.48	25/3/2	6.52
WOA	26/4/0	9.38	30/0/0	9.64	30/0/0	8.34	29/1/0	7.34
AO	25/5/0	5.96	29/1/0	4.57	29/1/0	4.21	28/1/1	4.55
TGCOA	29/1/0	11.41	30/0/0	11.79	30/0/0	12.41	30/0/0	12.21
ZOA	23/7/0	6.48	28/2/0	6.50	28/2/0	6.28	26/3/1	6.34
PO	22/8/0	5.93	29/1/0	6.36	29/0/1	6.07	26/3/1	5.59
MIPO	24/6/0	8.10	30/0/0	8.75	30/0/0	9.38	29/1/0	10.10
CGBPO	21/8/1	3.92	27/3/0	4.94	29/0/1	4.72	26/2/2	4.17
STPO	*∼*	1.76	*∼*	1.21	*∼*	1.21	*∼*	1.46

#### CEC2017 experimental analysis

This section presents the systematic experimental results of STPO on the CEC 2017 test dataset across four dimensionality levels. Statistical results indicate that STPO achieved average rankings of 1.76, 1.21, 1.21, and 1.46. It fully demonstrates its outstanding comprehensive optimization abilities.What’s more, STPO secures an outstanding average ranking of 1.21 in both 30-dimensional and 50-dimensional test environments, clearly outpaces its performance in 10-dimensional and 100-dimensional settings.This finding speaks to the algorithm’s steadily growing adaptability to medium and high dimensional optimization problems.

In addition to the average-rank results, the percentage improvement analysis further confirms the advantage of STPO over PO-family optimizers on CEC2017. Compared with the original PO, STPO achieves an overall mean improvement of 36.78% and a median improvement of 24.02%. Compared with MIPO and CGBPO, STPO achieves overall mean improvements of 48.17% and 30.23%, respectively, with corresponding median improvements of 43.08% and 16.86%. When PO, MIPO, and CGBPO are jointly considered as PO-family baselines, STPO obtains an overall mean improvement of 38.39% and a median improvement of 24.59%. Moreover, STPO achieves better mean objective values in 333 out of 348 PO-family comparisons, indicating that the proposed multi-strategy enhancement provides consistent improvement over the original PO framework and representative PO variants.

From the performance of test functions varying in complexity, STPO demonstrates a distinct advantage in handling nonlinear and multimodal problems:

When tested on the 10-dimensional unimodal function F1, STPO displayed rapid convergence comparable to the classical PSO algorithm, strongly demonstrating its efficiency in simple optimization tasks. As problem complexity increases, the inherent advantages of the STPO mechanism become increasingly evident. Across tests involving the multi-peak function F12, the mixed function F17, and the composite function F24, STPO comprehensively outperformed all comparison algorithms, demonstrating superiority in both convergence accuracy and convergence speed.

STPO’s performance in 30 and 50 dimensions further solidifies its competitive edge. In the 30-dimensional experiments, box plot statistical distributions reveal that STPO yields more concentrated solution sets. Upon transitioning to the 50-dimensional space, STPO’s performance advantage amplified further. Across the selected representative test functions, its convergence curves and statistical metrics consistently outperformed the comparison algorithms. This demonstrates that the STPO operator effectively mitigates the search efficiency degradation associated with dimensionality growth, making it highly suitable for tackling complex problems in large-scale optimization environments.

Even in settings of extremely high dimension, STPO maintains an average ranking of 1.46, outperforming all other compared algorithms. Yet when set against the experimental data from 50-dimensional tests, a clear trend emerges. The algorithm sees a measurable degree of performance degradation in spaces of ultra high dimension. This reveals clear room for further optimization, specifically in the algorithm’s capacity to address the curse of dimensionality.

Figure 14 uses a bar chart to present the average ranking of each compared algorithm on the CEC 2017 test suite. The pattern shown in the figure is unambiguous. Across all tested dimensions, STPO consistently maintains the lowest bar height.

Figure 15 presents a radar chart illustrating the specific performance distribution of each algorithm across 30 test functions from F1 to F30. The radar chart envelope of the STPO algorithm is significantly smaller than that of other algorithms, demonstrating the most outstanding comprehensive performance across all evaluation dimensions and overall superior performance.

#### CEC2022 experimental analysis

This subsection summarizes the comparative test results of STPO on the CEC 2022 benchmark dataset. It is clear that the STPO algorithm achieved average rankings of 2.15 and 1.74 for 10 and 20 dimensions respectively, with its overall performance metrics ranking first among all comparison algorithms.

The percentage improvement analysis also verifies the effectiveness of STPO on the CEC2022 benchmark suite. Compared with the original PO, STPO achieves an overall mean improvement of 9.95% and a median improvement of 2.30%. Compared with MIPO and CGBPO, STPO achieves overall mean improvements of 18.66% and 12.27%, respectively, with corresponding median improvements of 4.89% and 0.93%. Considering PO, MIPO, and CGBPO as PO-family baselines, STPO obtains an overall mean improvement of 13.63% and a median improvement of 1.94%. In addition, STPO obtains better mean objective values in 65 out of 72 PO-family comparisons, demonstrating that the proposed mechanisms can still provide stable enhancement on the newer CEC2022 test suite.

**Table 4:** Summarized experimental results of comparison experiments in CEC2022.

MAs	10Dim		20Dim	
	+/*≈*/-	Avg. Ranks	+/*≈*/-	Avg. Ranks
GA	10/2/0	10.00	12/0/0	9.83
PSO	8/3/1	6.92	10/1/1	6.25
HHO	10/2/0	8.00	10/1/1	6.50
DBO	10/2/0	8.17	11/1/0	9.58
POA	7/3/2	4.17	11/1/0	6.75
WOA	9/3/0	9.50	12/0/0	8.67
AO	10/2/0	5.42	10/2/0	5.42
TGCOA	11/1/0	11.25	12/0/0	12.25
ZOA	8/3/1	6.25	10/2/0	6.42
PO	8/4/0	6.17	10/2/0	5.00
MIPO	9/3/0	7.75	12/0/0	8.67
CGBPO	8/4/0	5.25	8/3/1	3.92
STPO	*∼*	2.15	*∼*	1.74

Convergence curve analysis across 10 dimensions shows that STPO achieves rapid convergence rates and high optimization accuracy for all representative test functions. Additionally, statistical distribution results from box plots indicate that the range of its solutions is significantly narrower compared to other comparison algorithms, with minimal fluctuation. This further emphasizes the robustness of the algorithm.

In further testing across 20 dimensions, STPO maintained its outstanding performance on the vast majority of representative test functions, achieving optimal optimization results. However, when tested against the unimodal function F1, STPO’s optimization accuracy was slightly inferior to that of the PSO algorithm. This result experimentally corroborates the NFL theorem in the field of optimization.

Despite minor shortcomings in some specific areas, STPO still offers significant advantages in solving highly challenging functions, such as multi-peak and composite problems. It demonstrates strong competitiveness in handling complex, nonlinear optimization tasks, proving its effectiveness in these demanding scenarios.

Figure 21 presents the average ranking histograms for each comparison algorithm across 10-dimensional and 20-dimensional scales in the CEC 2022 test dataset. The figure quantitatively reflects STPO’s consistently top-ranked average performance among the tested algorithms, confirming its exceptional capability in handling optimization tasks of varying scales.

Figure 22 depicts the specific ranking performance of each algorithm across 12 test functions from F1 to F12 using radar charts. The results indicate that it maintains a leading position in overall performance even in 20 dimensions.

Building upon the macro-level ranking analysis, to delve deeper into the performance distribution characteristics of algorithms across specific test functions, Fig. 22 employs a radar chart to illustrate the precise ranking performance of each algorithm across twelve test functions, from F1 to F12. Experimental results reveal that the radar chart envelope area corresponding to STPO is significantly smaller than that of other comparison algorithms, indicating it achieves the lowest ranking values across the vast majority of test functions. This minimal envelope characteristic not only validates STPO’s exceptional versatility across heterogeneous search spaces, but also demonstrates the algorithm’s high equilibrium across optimization problems with diverse mathematical properties. This effectively circumvents the common pitfall of traditional optimization algorithms failing on specific functions (Table 5).

### Ablation experiments on the CEC2017 test set for 30-dimensional and 50-dimensional models

To independently evaluate the performance contributions of each proposed strategy, ablation experiments were conducted on the CEC2017 benchmark dataset for 30D and 50D. First, the relevant algorithms are defined with the following abbreviations: PO denotes the original standard PO algorithm; PO-APM denotes PO incorporating the Alertness Mechanism (APM) from Sparrow Search; PO-EES denotes PO combined with the Experiential Exchange Strategy (EES); PO-DSP denotes PO integrated with the Differential Scale Perturbation (DSP) operator; STPO denotes the complete algorithm proposed in this paper. Table 6 summarizes the average rankings of each algorithm in the ablation experiments on the CEC2017 benchmark dataset. Detailed experimental data are provided in Appendix C.

**Table 5:** Summarized experimental results of comparison experiments in CEC2017.

MAs	Avg. Ranks	
	30Dim	50Dim
PO	4.00	4.09
PO-APM	2.98	2.74
PO-EES	3.02	2.98
PO-DSP	3.33	3.68
STPO	1.67	1.51

The ablation experiment results indicate that each individual strategy contributes to varying degrees in enhancing the performance of the PO algorithm. However, when only one strategy is introduced, the resulting performance improvement is typically confined to specific search phases–primarily boosting either early-stage global exploration capabilities or late-stage local exploitation capabilities. This phased advantage often leads to uneven algorithmic performance across different search phases, thereby constraining the stable improvement of overall performance to some extent. Consequently, this phenomenon further highlights the importance of adopting a comprehensive algorithm design framework.

A single strategy struggles to simultaneously balance global diversity and local search precision. By integrating the Alertness Mechanism (APM) from Sparrow Search Algorithm, the Experienced Exchange Strategy (EES), and the Worst-Guided Differential Scale Perturbation (DSP), the complementary strengths of each strategy can be fully leveraged across different search phases, constructing a synergistic search mechanism. This framework supports early-stage exploration and promotes refined convergence optimization in the later stages. Consequently, STPO enhances the coordination between global search and local refinement, contributing to the overall performance improvement observed in the CEC benchmark tests.

Experimental results also indicate that the combined effect of the three strategies consistently outperforms their individual applications in STPO, revealing significant synergistic effects that achieve more robust and balanced optimization performance. For details, please refer to Appendix C.

### Population diversity and exploration–exploitation analysis

To further investigate the search behavior of STPO, population diversity and exploration–exploitation analyses were conducted on representative 30-dimensional CEC2017 functions, including F1, F4, F12, F17, F24, and F27. Since STPO is developed from the PO framework, PO, MIPO, CGBPO, and STPO were selected for diversity comparison. Each algorithm was independently executed 30 times under the same population size and iteration budget.

14 $$\begin{aligned} Div(t)= \frac{1}{D} \sum _{d=1}^{D} \frac{1}{N} \sum _{i=1}^{N} \left| \textrm{median}\left( x_d(t)\right) -x_{id}(t) \right| \end{aligned}$$

where *N* is the population size, *D* is the problem dimension, $x_{id}(t)$ denotes the value of the *d*-th dimension of the *i*-th individual at iteration *t*, and $\textrm{median}(x_d(t))$ represents the median value of all individuals in the *d*-th dimension. A larger *Div*(*t*) indicates a more dispersed population, whereas a smaller value indicates stronger population aggregation.

15 $$\begin{aligned} Exploration(t)=\frac{Div(t)}{Div_{\max }}\times 100\% \end{aligned}$$

16 $$\begin{aligned} Exploitation(t)= \frac{\left| Div(t)-Div_{\max }\right| }{Div_{\max }} \times 100\% \end{aligned}$$

where $Div_{\max }$ denotes the maximum diversity value during the whole iterative process. The exploration ratio reflects the dispersion degree of the population, while the exploitation ratio reflects the convergence degree toward promising regions.

**Fig. 23:**
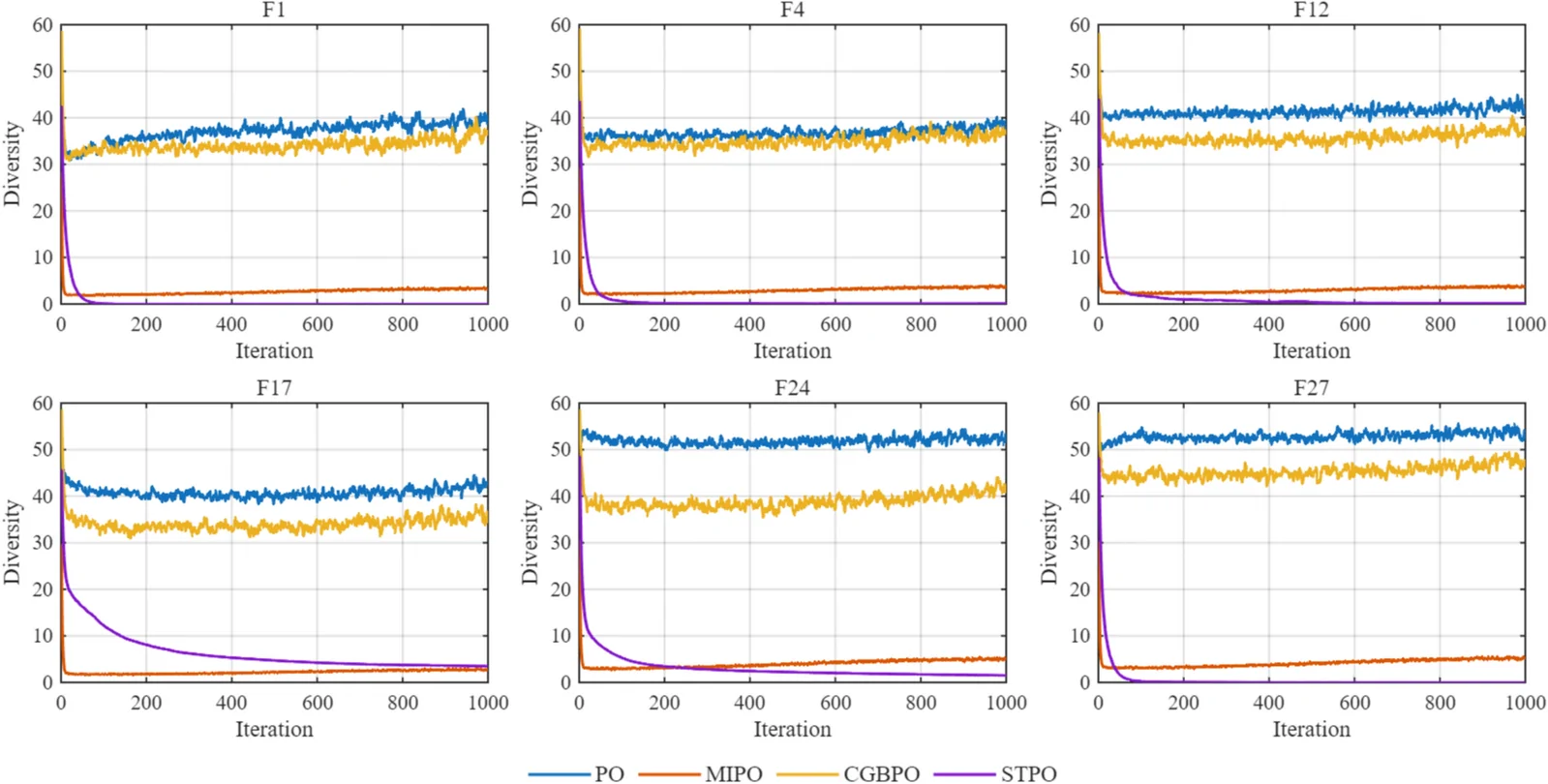
Population Diversity Curves on CEC2017 30D Representative Functions.

Figure 23 shows the population diversity curves of PO-family optimizers on representative CEC2017 30-dimensional functions. It can be observed that PO and CGBPO maintain relatively high diversity during most iterations, while STPO shows a rapid diversity contraction after the initial search stage.This indicates that STPO adaptively regulates population diversity during the optimization process, maintaining sufficient exploration in the early stage and then progressively guiding individuals toward promising regions for refined exploitation. This behavior is particularly clear on F1, F4, F12, and F27. For more complex functions such as F17 and F24, the diversity of STPO decreases more gradually, suggesting that STPO can preserve a certain level of exploration when the landscape is more difficult. Therefore, the diversity results reveal an adaptive population contraction behavior of STPO rather than a passive loss of diversity.

**Fig. 24:**
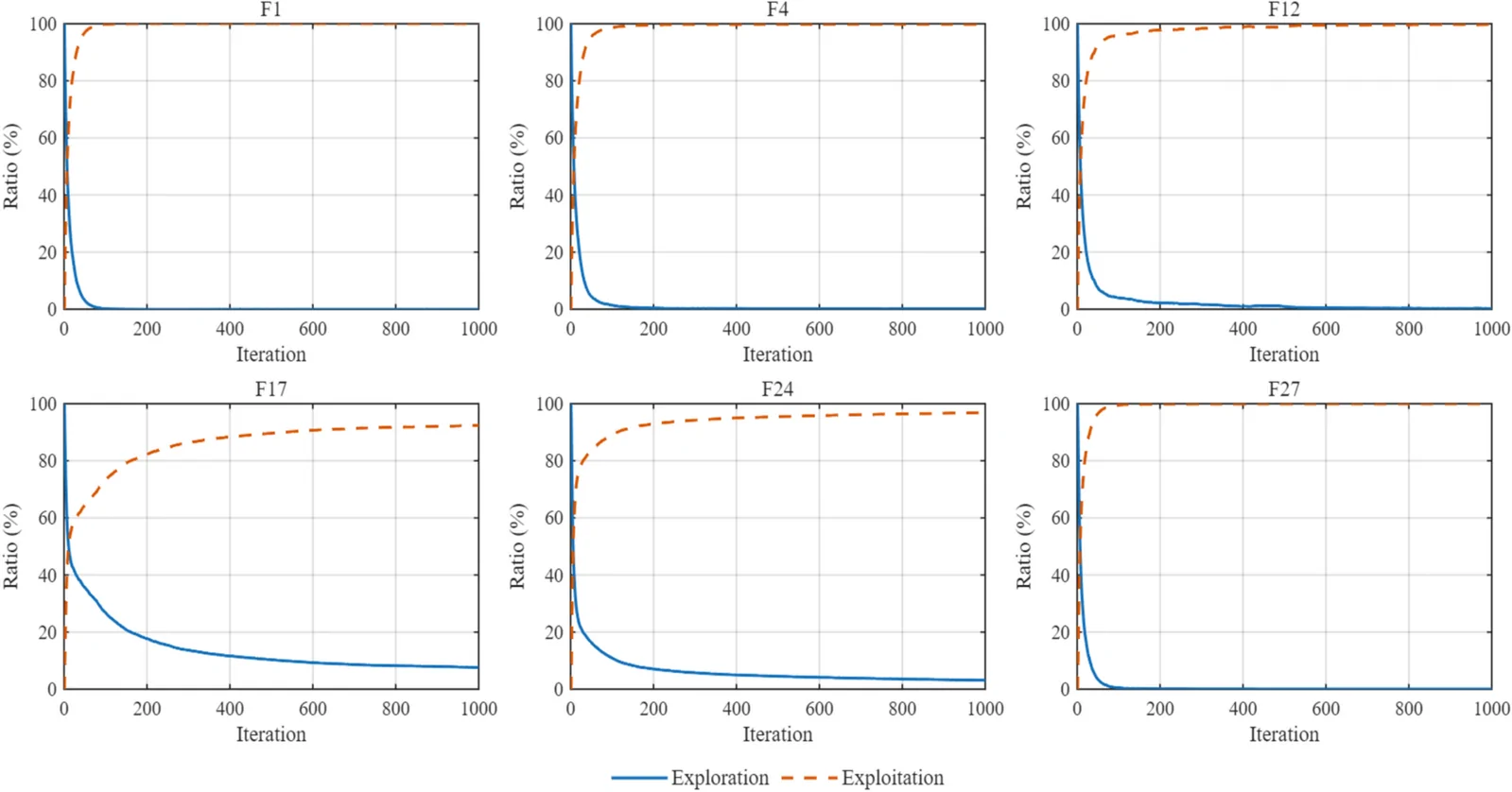
STPO Exploration–Exploitation Curves on CEC2017 30D.

Figure 24 further presents the exploration–exploitation behavior of STPO. At the beginning of the search process, STPO exhibits a high exploration ratio, indicating that the population is initially distributed over a relatively wide search region. As the iteration proceeds, the exploration ratio decreases and the exploitation ratio increases rapidly, showing that STPO can efficiently shift from global exploration to local exploitation. For F17 and F24, the exploration ratio decreases more slowly than that on simpler functions, which suggests that STPO can retain exploration for a longer period when facing more complex fitness landscapes.Overall, these results suggest that STPO achieves an adaptive coordination between exploration and exploitation. By maintaining adequate search diversity in the early stage and gradually strengthening exploitation around promising regions in the later stage.

## Numerical experiments in engineering problems

To ensure the representativeness and reliability of the test results, this section selected five classic engineering optimization benchmark problems as primary test cases, including the cantilever beam design problem (CBDP)^50^, three-bar truss design problem (TBTDP)^51^, tubular column design problem (TCDP)^52^, piston link design problem (PLDP)^53^, and weight minimization design problem for reducers (WMSR)^54^.

We resolved the above issues through numerical experiments, employing the optimization algorithms listed in Table 2 as comparison algorithms. Under identical experimental settings, each algorithm was run independently 15 times. Also, for fairness in comparison, the termination condition for each run was uniformly set to a maximum of 1000 iterations.The best average result among all comparison algorithms is shown in bold.

For the constrained engineering design problems, a unified constraint-evaluation protocol was adopted for all compared algorithms. Specifically, all algorithms were evaluated using the same built-in engineering benchmark module, in which the objective function, design-variable bounds, and constraint functions were kept identical for each problem. During the search process, candidate solutions were evaluated through the same objective-and-constraint evaluation interface, and the same boundary-control rule was applied to avoid unfair differences caused by implementation details. The final reported solutions were further checked according to the original constraint functions. To improve reproducibility, the exact mathematical formulations of all engineering benchmark problems, including objective functions, design variables, bounds, constraints, and references, are provided in Appendix D.

### Experimental testing of cantilever beam design problems

The goal of the cantilever beam design problem (CBDP) is to reduce the total weight or material usage of the beam, it must meet strength and structural requirements. The problem involves several continuous design variables. These variables describe the size of each segment of the cantilever beam, such as its width and height.

**Fig. 25:**
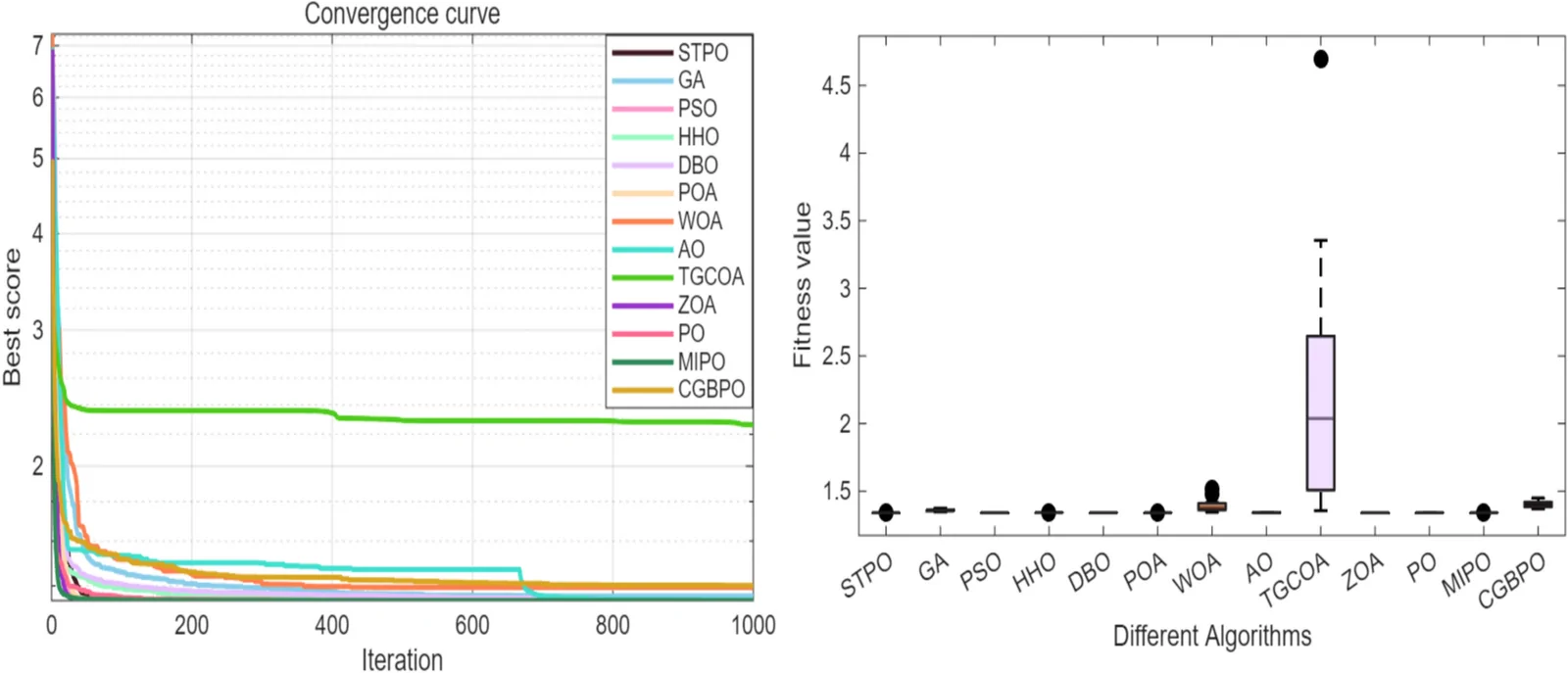
Convergence curves and box plots of CBDP.

By optimizing these design variables, the cantilever arm can achieve optimal material utilization while ensuring sufficient load-bearing capacity and structural safety.The formulation adopted in this study follows the commonly used version reported in reference^50^. Specific models and equations are detailed in Appendix D.1. Table 6 summarizes experimental results and statistical analysis, while Fig. 25 displays convergence curves and box plots.

**Table 6:** CBDP experimental results and statistical analysis.

MAs	mean	best	worst	std	Avg.runtime
GA	1.3586E+00$^{+}$	1.3459E+00	1.3734E+00	7.6098E-03	6.3113E-01
PSO	1.3400E+00$^{=}$	1.3400E+00	1.3400E+00	1.6072E-06	3.5502E-01
HHO	1.3415E+00$^{+}$	1.3403E+00	1.3429E+00	6.8748E-04	9.9643E-01
DBO	1.3400E+00$^{=}$	1.3400E+00	1.3401E+00	3.7180E-05	6.6276E-01
POA	1.3400E+00$^{=}$	1.3400E+00	1.3400E+00	1.1356E-05	6.2646E-01
WOA	1.3940E+00$^{+}$	1.3440E+00	1.5161E+00	4.7588E-02	4.4609E-01
AO	1.3414E+00$^{+}$	1.3404E+00	1.3426E+00	6.8526E-04	9.1835E-01
TGCOA	2.2615E+00$^{+}$	1.3551E+00	4.6979E+00	9.3422E-01	6.8510E-01
ZOA	1.3400E+00$^{=}$	1.3400E+00	1.3400E+00	1.0188E-05	6.2552E-01
PO	1.3405E+00$^{+}$	1.3400E+00	1.3414E+00	4.6912E-04	2.6734E+01
MIPO	1.3400E+00$^{=}$	1.3400E+00	1.3402E+00	6.8724E-05	8.5262E-01
CGBPO	1.4017E+00$^{+}$	1.3693E+00	1.4496E+00	2.2475E-02	2.7982E+00
STPO	**1.3400E+00**	1.3400E+00	1.3400E+00	7.4705E-06	6.1619E-01

The proposed STPO algorithm ranks among the top three in metrics of mean, standard deviation, and average running time, exhibiting exceptional comprehensive capabilities. Notably, it achieves an optimal mean value of 1.3400E+00, and its performance in addressing this issue significantly outperforms PO and its variant MIPO, further validating StPO’s formidable potential for engineering applications.

### Experimental testing of three-bar truss design problems

The goal of the Three-Bar Truss Bridge Design Problem (TBTDP) is to minimize the total volume of a three-bar truss while ensuring it meets engineering requirements like structural safety and normal serviceability performance. This problem usually involves several design variables that define the size of each truss member. At the same time, constraints such as buckling, deflection, and stress are applied to each member to make sure the final design follows engineering standards for stability, stiffness, and strength.

The key parameters of this problem are $A_1$ and $A_2$. The formulation adopted in this study follows the commonly used version reported in reference^51^. The specific models and equations can be found in Appendix D.2.

**Fig. 26:**
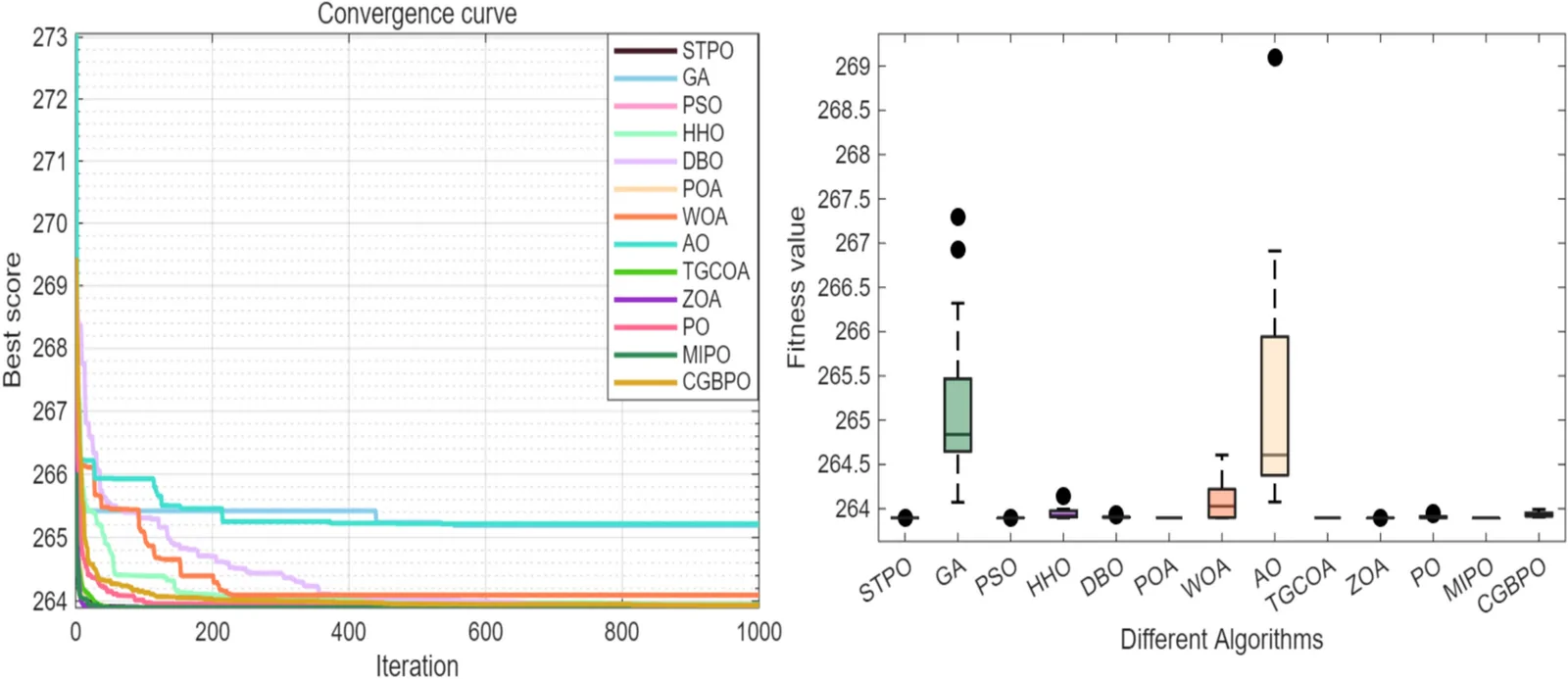
Convergence curves and box plots of TBTDP.

Table 7 summarizes the experimental results and statistical analysis, while Fig. 26 presents the convergence curves and box plots.

**Table 7:** TBTDP experimental results and statistical analysis.

MAs	mean	best	worst	std	Avg.runtime
GA	2.6519E+02$^{+}$	2.6407E+02	2.6730E+02	9.7607E-01	1.1751E+00
PSO	2.6390E+02$^{=}$	2.6390E+02	2.6390E+02	7.4648E-04	8.8341E-01
HHO	2.6395E+02$^{+}$	2.6390E+02	2.6414E+02	6.2562E-02	2.4154E+00
DBO	2.6391E+02$^{+}$	2.6390E+02	2.6394E+02	1.0524E-02	1.2244E+00
POA	2.6390E+02$^{=}$	2.6390E+02	2.6390E+02	1.1768E-13	1.7446E+00
WOA	2.6409E+02$^{+}$	2.6390E+02	2.6461E+02	2.3026E-01	1.0242E+00
AO	2.6522E+02$^{+}$	2.6408E+02	2.6910E+02	1.3895E+00	2.0121E+00
TGCOA	2.6390E+02$^{=}$	2.6390E+02	2.6390E+02	1.1768E-13	1.2405E+00
ZOA	2.6390E+02$^{=}$	2.6390E+02	2.6390E+02	8.3364E-04	1.7405E+00
PO	2.6391E+02$^{+}$	2.6390E+02	2.6395E+02	1.5797E-02	8.3384E+01
MIPO	2.6390E+02$^{=}$	2.6390E+02	2.6390E+02	3.2999E-04	1.9103E+00
CGBPO	2.6394E+02$^{+}$	2.6390E+02	2.6399E+02	2.8041E-02	4.3944E+00
STPO	**2.6390E+02**	2.6390E+02	2.6390E+02	2.1463E-04	1.1875E+00

It can be observed that multiple algorithms simultaneously achieved the optimal mean value. However, in the experiments, STPO demonstrated higher stability and faster convergence speed. This indicates that STPO exhibits greater robustness in repeated testing and possesses significant competitive advantages.

### Experimental testing of tubular column design problems

The Tubular Column Design Problem (TCDP) aims to reduce the total cost of tubular columns. It ensures that the columns meet technical requirements, such as pressure load capacity and structural stability. The problem defines the geometric parameters of different column sections using various design variables. These variables help the column safely bear axial pressure loads. They also ensure the design is both economically efficient and technically feasible.

This case includes two key design variables, namely the mean diameter of the column $(d = x_1)$ and the tube-wall thickness $(t = x_2)$. The formulation adopted in this study follows the commonly used version reported in reference^52^. The corresponding mathematical model and constraint equations are provided in Appendix D.3.

**Fig. 27:**
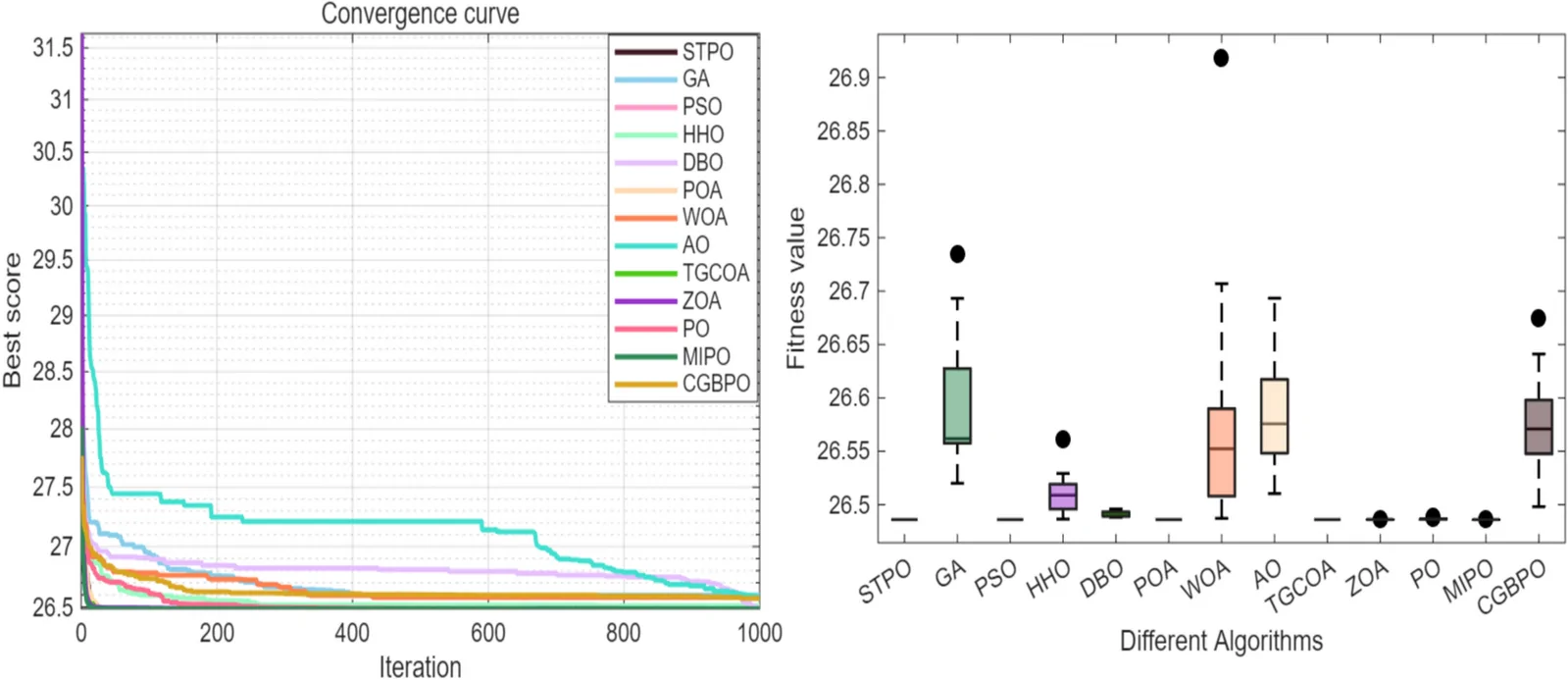
Convergence curves and box plots of TCDP.

Table 8 summarizes the experimental results and statistical analysis of the comparative algorithms on the TCDP, while Fig. 27 presents the convergence curves and box plots of different algorithms to facilitate an intuitive comparison of their optimization performance and robustness.

**Table 8:** TCDP experimental results and statistical analysis.

MAs	mean	best	worst	std	Avg.runtime
GA	2.6597E+01$^{+}$	2.6520E+01	2.6734E+01	6.3688E-02	7.9374E-01
PSO	2.6486E+01$^{=}$	2.6486E+01	2.6486E+01	7.3548E-15	4.5075E-01
HHO	2.6510E+01$^{+}$	2.6486E+01	2.6561E+01	1.9884E-02	1.3103E+00
DBO	2.6492E+01$^{+}$	2.6488E+01	2.6496E+01	2.6450E-03	7.6952E-01
POA	2.6486E+01$^{=}$	2.6486E+01	2.6486E+01	7.3548E-15	9.0149E-01
WOA	2.6579E+01$^{+}$	2.6487E+01	2.6919E+01	1.1024E-01	6.0955E-01
AO	2.6582E+01$^{+}$	2.6510E+01	2.6693E+01	4.9538E-02	1.1758E+00
TGCOA	2.6486E+01$^{=}$	2.6486E+01	2.6486E+01	7.3548E-15	8.0959E-01
ZOA	2.6486E+01$^{=}$	2.6486E+01	2.6486E+01	1.3195E-04	8.9509E-01
PO	2.6487E+01$^{+}$	2.6486E+01	2.6488E+01	7.0823E-04	4.2942E+01
MIPO	2.6486E+01$^{=}$	2.6486E+01	2.6486E+01	5.2505E-06	1.1511E+00
CGBPO	2.6573E+01$^{+}$	2.6498E+01	2.6674E+01	4.7066E-02	3.0705E+00
STPO	**2.6486E+01**	2.6486E+01	2.6486E+01	7.3548E-15	7.6690E-01

After a comprehensive comparison of evaluation metrics including mean, standard deviation, and average runtime, both the STPO algorithm and the PSO algorithm demonstrated the most outstanding overall performance. Specifically, both achieved a mean value of 2.6486E+01. These two algorithms not only delivered the optimal average optimization results but also exhibited significant advantages in two critical areas: computational efficiency and result stability.

### Experimental testing of piston link design problems

The objective of the Piston Link Design Problem (PLDP) is to minimize the required oil volume under the condition where the piston rod rises to 45 degrees, based on the corresponding mathematical model.

This problem typically involves multiple design variables used to determine the key geometric position parameters of the valve component, including $H(x_1)$, $B(x_2)$, $D(x_3)$, and $X(x_4)$.

Optimizing these design variables improves the operational efficiency of the hydraulic system. It also minimizes resource consumption. Meanwhile, the movement and functional requirements of the mechanism are met.

**Fig. 28:**
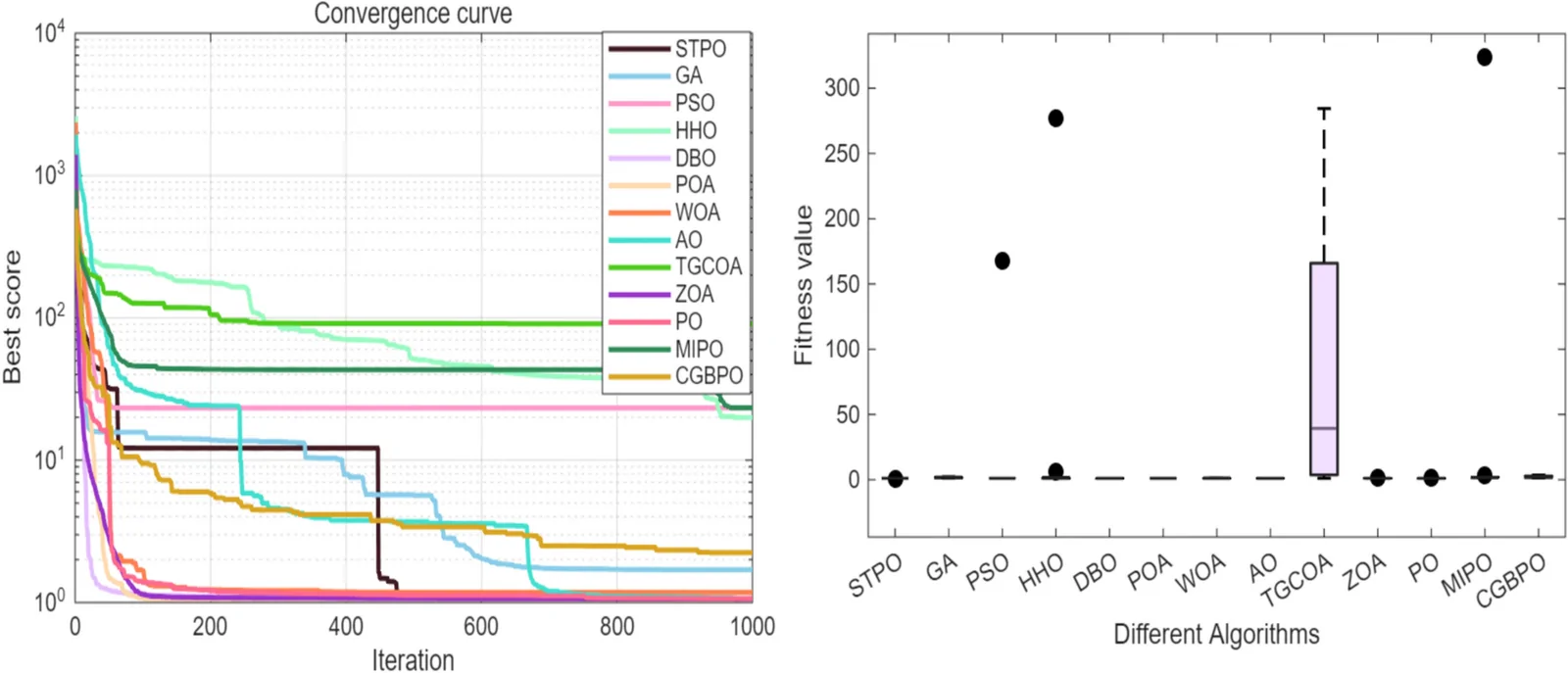
Convergence curves and box plots of PLDP.

The formulation adopted in this study follows the commonly used version reported in reference^53^.The specific models and equations are provided in Appendix D.4. Table 9 summarizes the experimental results and statistical analysis, while Fig. 28 presents the convergence curves and box plots.

**Table 9:** PLDP experimental results and statistical analysis.

MAs	mean	best	worst	std	Avg.runtime
GA	1.7039E+00$^{+}$	1.1402E+00	2.3150E+00	3.5699E-01	7.5107E-01
PSO	2.3246E+01$^{+}$	1.0574E+00	1.6747E+02	5.8556E+01	4.7791E-01
HHO	1.9920E+01$^{+}$	1.0576E+00	2.7716E+02	7.1174E+01	1.2275E+00
DBO	1.0586E+00$^{=}$	1.0578E+00	1.0595E+00	4.8344E-04	7.2153E-01
POA	1.0574E+00$^{=}$	1.0574E+00	1.0574E+00	8.5606E-14	8.2812E-01
WOA	1.1812E+00$^{+}$	1.0684E+00	1.3557E+00	9.2826E-02	5.6559E-01
AO	1.0678E+00$^{+}$	1.0618E+00	1.0776E+00	5.6290E-03	1.1293E+00
TGCOA	9.0609E+01$^{+}$	1.0700E+00	2.8448E+02	1.0453E+02	7.5945E-01
ZOA	1.0630E+00$^{+}$	1.0575E+00	1.1163E+00	1.5427E-02	8.4669E-01
PO	1.0707E+00$^{+}$	1.0574E+00	1.1624E+00	2.9168E-02	3.7844E+01
MIPO	2.3311E+01$^{+}$	1.2569E+00	3.2419E+02	8.3239E+01	1.0890E+00
CGBPO	2.2381E+00$^{+}$	1.3379E+00	3.6229E+00	6.9477E-01	2.9585E+00
STPO	**1.0574E+00**	1.0574E+00	1.0574E+00	2.3738E-16	7.5367E-01

The PLDP evaluation performed a detailed analysis of the algorithms’ performance based on multiple measurement criteria. These included the mean, standard deviation, and average runtime. The proposed STPO algorithm finished first in the overall ranking. It showed the best balance between optimization accuracy, stability, and computational power.

The PSO algorithm, which previously demonstrated superior performance in TCDP problems, showed a significant drop in optimization capability in this case study. This result supports the NFL thesis’s conclusions at the experimental level.Variations in objective functions, constraint conditions, and search domain structures lead to different degrees of adaptability of optimization algorithms to problem characteristics. Even the same algorithm can produce very different optimization results when applied to different problems.

### Experimental testing of the weight minimization design problem for speed reducers

The goal of the Weight Minimization Design Problem for Speed Reducers (WMSR) is to reduce the total weight of the reducer. Simultaneously, it must meet essential technical requirements, including transmission performance, strength, and structural limitations. The challenge lies in achieving an optimal balance between minimizing weight and meeting performance standards.

This problem involves several design variables that define the key components of the reducer. These include gear sizes, shaft shapes, and housing parameters. Optimizing these variables allows for a lighter structure and better material use. It ensures that transmission performance and structural reliability are not compromised.

**Fig. 29:**
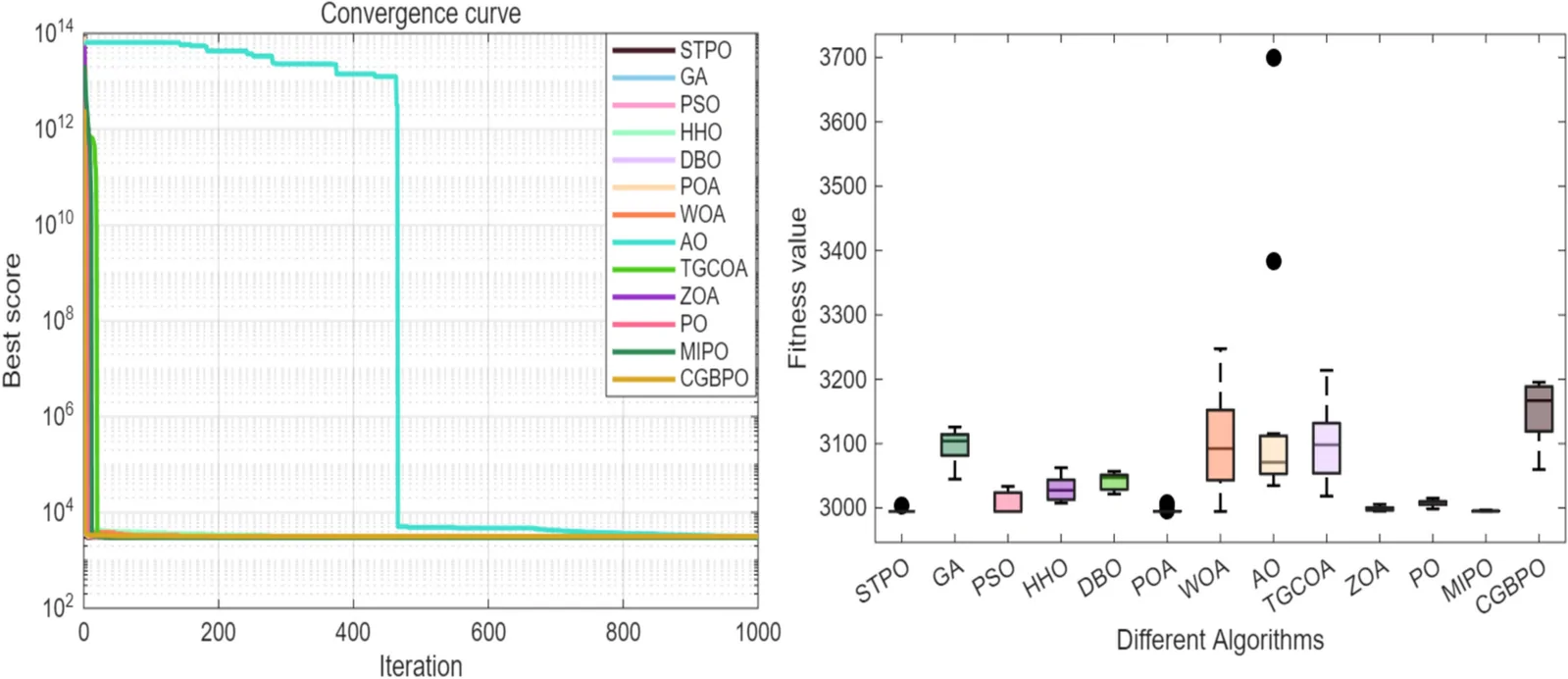
Convergence curves and box plots of WMSR.

The formulation adopted in this study follows the commonly used version reported in reference^54^. Specific models and equations are detailed in Appendix D.5. Table 10 summarizes the experimental results and statistical analysis, while Fig. 29 displays the convergence curve and box plot.

**Table 10:** WMSR experimental results and statistical analysis.

MAs	mean	best	worst	std	Avg.runtime
GA	3.0987E+03$^{+}$	3.0448E+03	3.1258E+03	2.1469E+01	1.6248E+00
PSO	3.0049E+03$^{+}$	2.9944E+03	3.0337E+03	1.7979E+01	1.3550E+00
HHO	3.0293E+03$^{+}$	3.0077E+03	3.0627E+03	1.8491E+01	3.5002E+00
DBO	3.0415E+03$^{+}$	3.0216E+03	3.0571E+03	1.2824E+01	1.7414E+00
POA	2.9961E+03$^{+}$	2.9944E+03	3.0073E+03	3.9366E+00	2.6328E+00
WOA	3.1007E+03$^{+}$	2.9946E+03	3.2473E+03	7.4552E+01	1.4855E+00
AO	3.1708E+03$^{+}$	3.0347E+03	3.7002E+03	2.3082E+02	3.0365E+00
TGCOA	3.0987E+03$^{+}$	3.0185E+03	3.2138E+03	5.2266E+01	1.7156E+00
ZOA	2.9989E+03$^{+}$	2.9951E+03	3.0054E+03	3.1372E+00	2.6661E+00
PO	3.0078E+03$^{+}$	2.9986E+03	3.0151E+03	4.7621E+00	1.2822E+02
MIPO	2.9953E+03$^{+}$	2.9944E+03	2.9968E+03	7.0555E-01	2.8297E+00
CGBPO	3.1500E+03$^{+}$	3.0598E+03	3.1953E+03	4.6655E+01	5.8447E+00
STPO	**2.9950E+03**	2.9944E+03	3.0038E+03	2.4104E+00	1.7156E+00

In the WMSR evaluation, the proposed STPO algorithm achieved optimal performance across all evaluation metrics, ranking first overall. Its optimization results not only outperformed several highly cited classical meta-heuristic algorithms but also demonstrated significant advantages over the PO algorithm and its various variants. This result fully demonstrates that the three improvement strategies introduced in this paper play a crucial role in enhancing the optimization accuracy, convergence performance, and stability of the PO algorithm, thereby effectively strengthening the algorithm’s overall competitiveness in complex engineering optimization problems.

## Application of STPO in mobile robot path planning problems

### Problem introduction and environment modeling

Mobile robot path planning aims to construct a collision-free shortest feasible path from the start point *s* to the end point *e* under given environmental constraints^55^. In this paper, the workspace is discretized using an occupancy grid, where the resolution of the occupancy grid map is *K*, i.e., the number of grid cells along the horizontal and vertical directions of the map. The environment is represented as a binary matrix $G \in \{0,1\}^{K \times K}$:

17 $$\begin{aligned} G(i,j)= \left\{ \begin{array}{ll} 1, & (i,j)\in \Omega _{obs},\\ 0, & (i,j)\in \Omega _{free}. \end{array} \right. \end{aligned}$$

where $\Omega _{obs}$ and $\Omega _{free}$ denote the obstacle set and the free (traversable) set, respectively. The start and goal points are denoted as:

18 $$\begin{aligned} s=(1,1), \qquad e=(K,K) \end{aligned}$$

To ensure fairness in comparing different algorithms, a random strategy generates four test maps and imposes a consistent computational budget on all algorithms–specifically, the same maximum iteration count and a 20-second time limit. Algorithm performance is primarily measured by the shortest feasible path length obtained, supplemented by comprehensive evaluation using statistical metrics such as average ranking.

### Objective function: minimum feasible path setting

Path length is defined as the sum of Euclidean distances between adjacent path points:

19 $$\begin{aligned} L(P)=\sum _{m=1}^{M-1}\left\Vert P_{m+1}-P_m \right\Vert _{2} \end{aligned}$$

where *P* denotes the path, $P_m$ is the *m*-th waypoint on the path, and *M* is the total number of waypoints.

Define the collision count function:

20 $$\begin{aligned} C(P)=\sum _{m=1}^{M}\mathbb {I}\!\left( P_m \in \Omega _{obs}\right) \end{aligned}$$

where $\mathbb {I}(\cdot )$ denotes the indicator function. To facilitate solving with a metaheuristic algorithm in a continuous domain, the constrained optimization problem is transformed into an unconstrained form with a penalty term:

21 $$\begin{aligned} \min _{x \in [1,K]^{K-2}} f(x)=L(P(x))+\lambda C(P(x)),\quad \lambda>0 \end{aligned}$$

Within 20 seconds, Equation (17) will output multiple sets of fitness values. The shortest attainable path corresponding to the superior fitness will serve as the primary evaluation metric. We will further calculate the average ranking over four sets of maps to reflect the algorithm’s overall stability.

### Test results

**Fig. 30:**
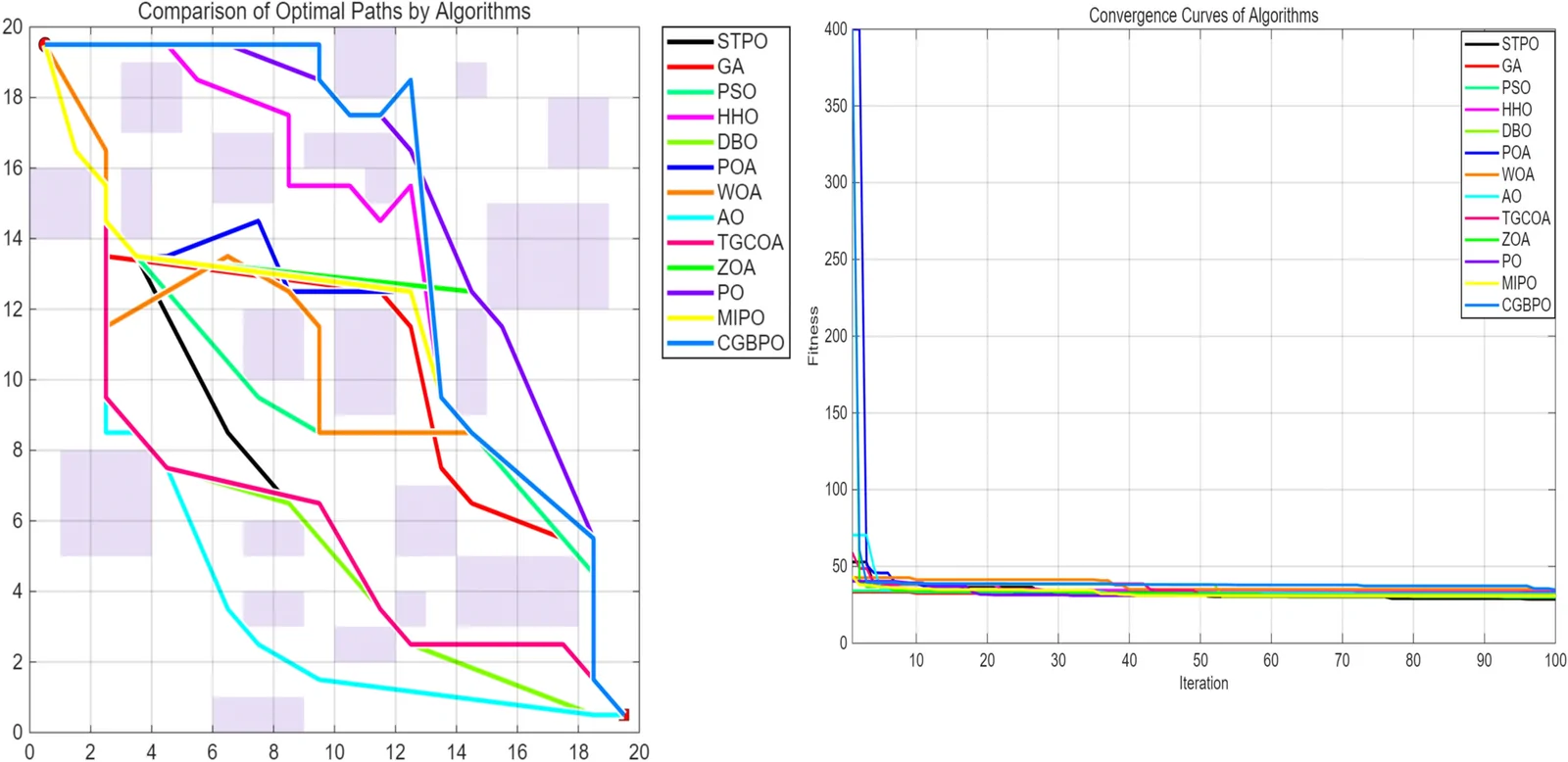
2D map and convergence curve of Map I.

**Fig. 31:**
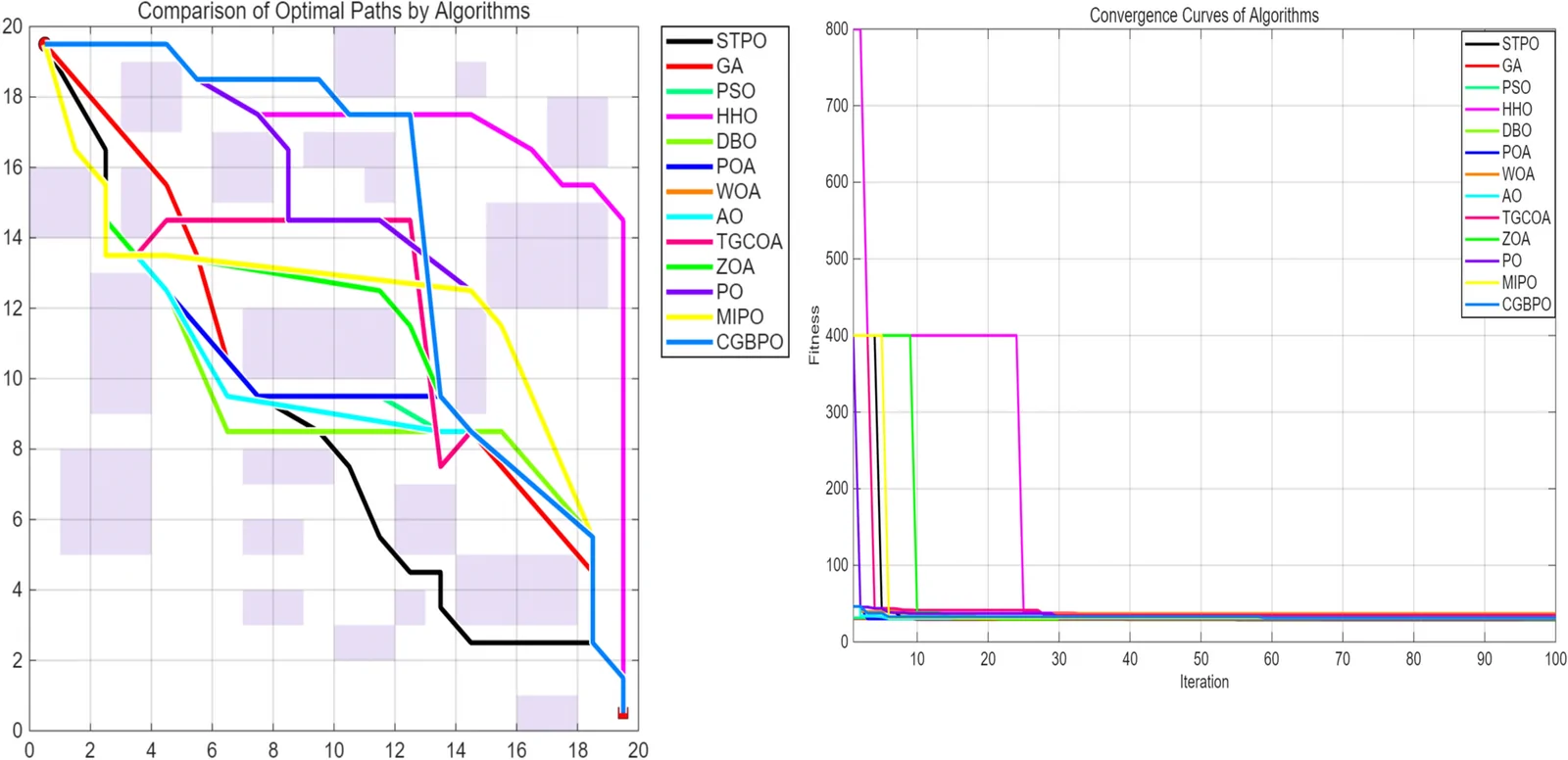
2D map and convergence curve of Map II.

**Fig. 32:**
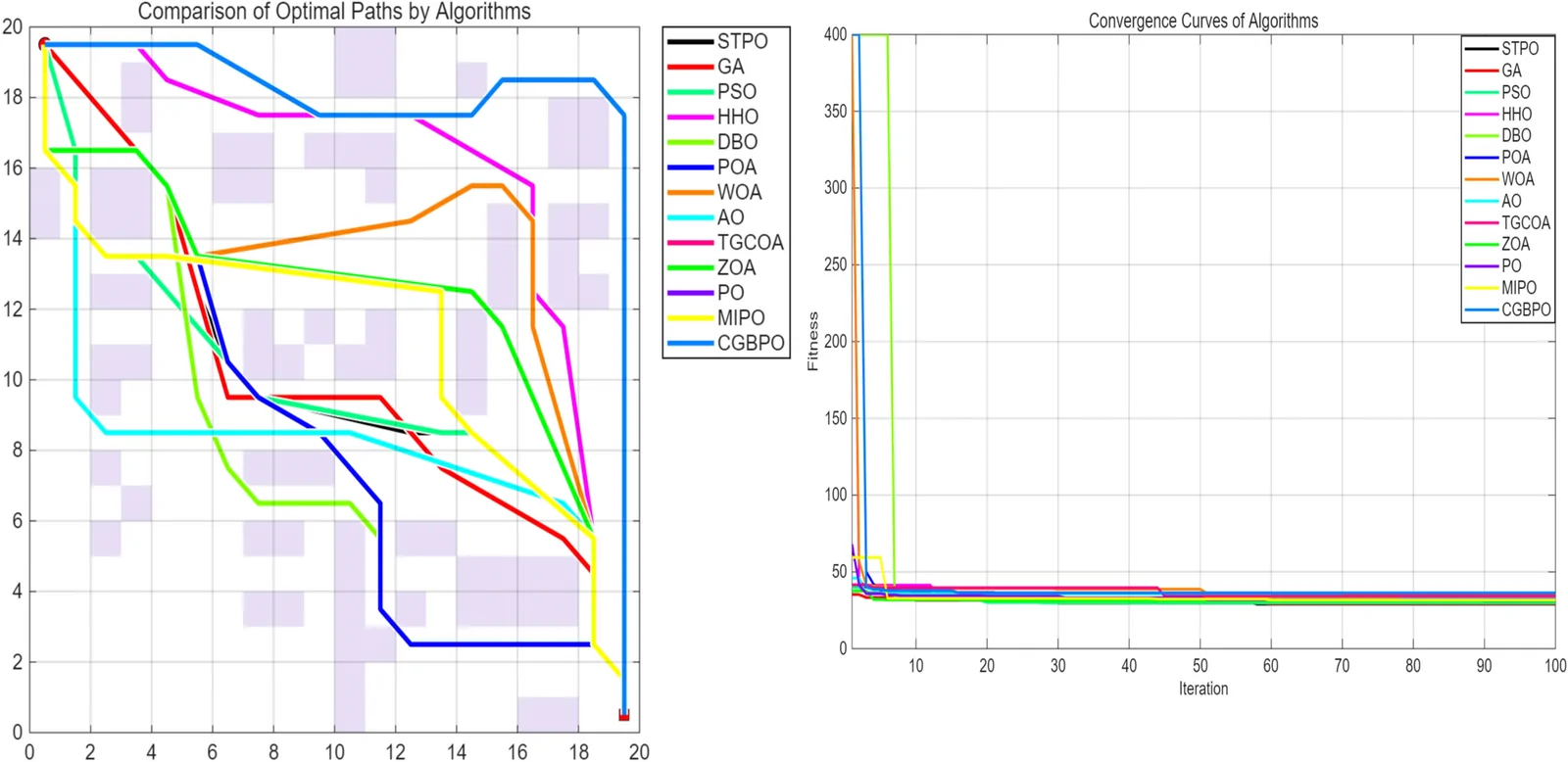
2D map and convergence curve of Map III.

**Fig. 33:**
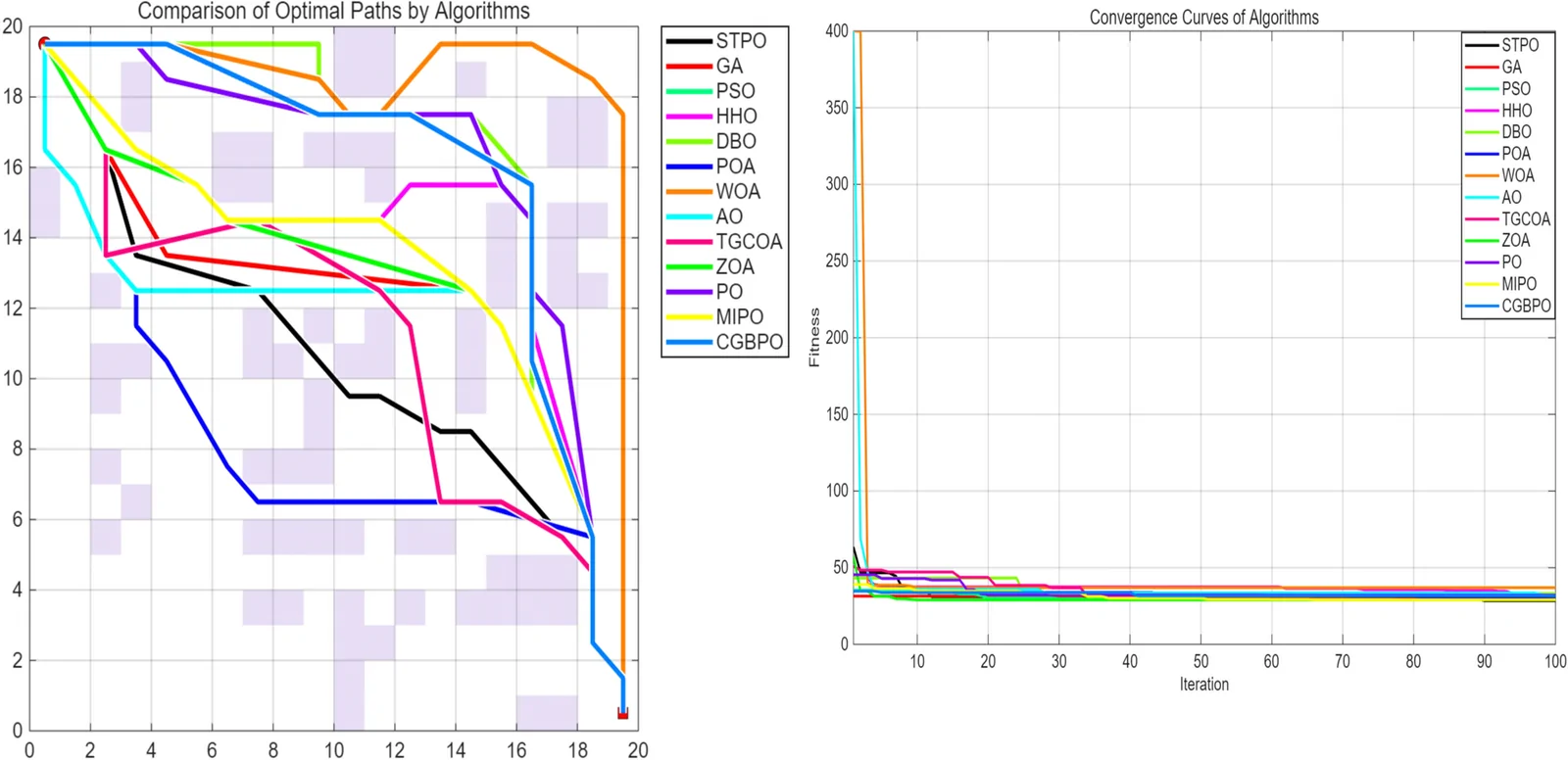
2D map and convergence curve of Map IV.

Table 11 presents the average ranking and shortest path statistics for each algorithm, with the minimum value for each measure highlighted in bold black. Figures 30, 31, 32, 33 combine convergence curves and 2D path visualization for the four test situations. These figures demonstrate the robustness and optimization efficiency of the proposed STPO algorithm under constrained computational budgets, based on both convergence behavior and solution quality.

**Table 11:** Shortest Paths and Average Ranking of Each Algorithm Across Four Maps.

MAs	Figure.30	Figure.31	Figure.32	Figure.33	Avg.rank
GA	3.157E+01	2.911E+01	3.050E+01	2.980E+01	4.00
PSO	2.995E+01	2.973E+01	3.038E+01	2.897E+01	3.33
HHO	3.332E+01	3.196E+01	2.951E+01	3.246E+01	7.00
DBO	3.054E+01	3.239E+01	3.614E+01	3.313E+01	10.67
POA	3.241E+01	3.112E+01	3.373E+01	3.104E+01	6.33
WOA	3.514E+01	3.511E+01	3.297E+01	3.699E+01	10.67
AO	3.217E+01	3.294E+01	3.324E+01	3.160E+01	8.33
TGCOA	3.135E+01	3.419E+01	4.433E+01	3.175E+01	11.00
ZOA	3.157E+01	3.124E+01	2.954E+01	2.896E+01	3.33
PO	3.100E+01	3.194E+01	3.199E+01	3.207E+01	7.67
MIPO	3.104E+01	3.171E+01	3.378E+01	2.904E+01	6.67
CGBPO	3.471E+01	3.630E+01	3.692E+01	3.166E+01	11.00
STPO	**2.863E+01**	**2.898E+01**	**2.799E+01**	**2.844E+01**	**1.00**

As can be seen, the vast majority of tested algorithms converged within 10 iterations, yielding different shortest paths. STPO excelled in completing the task, emerging as the top performer.

Therefore, it can be concluded that under a unified computational budget constraint, the proposed STPO algorithm can obtain relatively optimal viable path solutions within shorter runtime. Combining the above convergence curve results, STPO demonstrates stronger global search capabilities and the ability to escape local optima during iterative optimization, ultimately converging to stability at a faster pace, reflecting high convergence efficiency and robustness. Furthermore, comparing the 2D path traces across four randomly generated maps reveals that paths generated by STPO consistently satisfy obstacle constraints while also demonstrating advantages in overall path length metrics.

The results collectively affirm the effectiveness and competitiveness of STPO in mobile robot path planning, making it particularly suitable for scenarios requiring rapid, high-quality path solutions under limited time constraints.

## Conclusion

In order to deal with difficult challenges in complex optimization problems, such as local optima traps, search stagnation, and the delicate balance between exploitation and exploration, this article proposes a new multi-strategy enhanced parrot optimization algorithm (STPO). It builds on the original foraging mechanism and includes a guard mechanism from sparrow search algorithms to increase the agility of individuals in escaping suboptimal regions. Combined with an experience exchange strategy, STPO improves information diffusion among individuals and strengthens the coordination between population-level search and individual refinement. The integration of a differential perturbation scale mechanism increases the algorithm’s probability of escaping local optima. By creating a framework for collaborative search that includes escape from vigilance, experience sharing, and differential control, the algorithm’s convergence stability and global optimization capabilities are significantly improved.

Complexity analysis indicates that STPO enhances performance without introducing additional time complexity. By eliminating the cumbersome sorting operations of PO, it actually reduces computational burden and demonstrates excellent scalability in high dimensions.

The performance of STPO was evaluated on the CEC2017 and CEC2022 benchmark datasets, with statistical significance verified using the Mann–Whitney U test and Friedman test. Ablation experiments revealed that a single strategy struggles to balance global exploration and local exploitation. The alert mechanism, experience exchange, and differential-scale perturbation exhibit complementary effects during the search process, and their synergistic interaction forms the core performance driver for enhancing the algorithm’s comprehensive optimization capabilities. Furthermore, the population diversity and exploration–exploitation analyses provide additional evidence that STPO exhibits an adaptive search transition, enabling effective early-stage exploration and efficient later-stage exploitation on representative CEC benchmark functions.

In 5 typical engineering numerical experiments, STPO consistently obtained high-quality feasible solutions, ranking among the top performers in terms of objective value and ruggedness indicators. This confirms its suitability not only for standard continuous benchmark functions but also for adapting well to diverse constraint structures and engineering design requirements.

Additionally, this paper applies STPO to path planning tasks for mobile robots. By modeling grid environments and handling penalty function constraints, it transforms collision-free shortest feasible path planning into an optimizable objective. The method was evaluated on four randomly generated maps under uniform computational budget constraints. Experimental results demonstrate that STPO consistently generates shorter and feasible path solutions within finite time constraints. Its overall best ranking further indicates the method’s robust computational efficiency and practical applicability under resource-constrained conditions.

Despite the competitive performance of STPO, several limitations should be acknowledged. First, although STPO shows strong performance on most CEC benchmark functions, its advantage becomes less pronounced on some simple unimodal functions and extremely high-dimensional cases, which indicates that further improvement is still needed for large-scale optimization. Second, the proposed method introduces additional strategy-selection logic, and its performance may be influenced by parameters such as the perturbation probability and population size. Although fixed settings were used in this study for fairness, adaptive parameter control may further improve robustness. Finally, the current study mainly focuses on continuous single-objective optimization and grid-based path planning. Future work will extend STPO to discrete, multi-objective, dynamic, and large-scale real-world optimization scenarios.

In the near future, we believe the STPO algorithm can efficiently integrate new technologies and concepts. For instance, it can play a pivotal role in complex multi-robot collaborative tasks, helping optimize robot operation paths and material scheduling on automated production lines to further enhance productivity. STPO can also be extended to big data environments for massive data grouping and classification. Its efficient global search capabilities particularly optimize model performance when handling high-dimensional data. In interdisciplinary fields like medical and biological informatics, STPO finds applications in medical image processing and optimizing personalized treatment plans.

## Figures and Tables

**Fig. 34:**
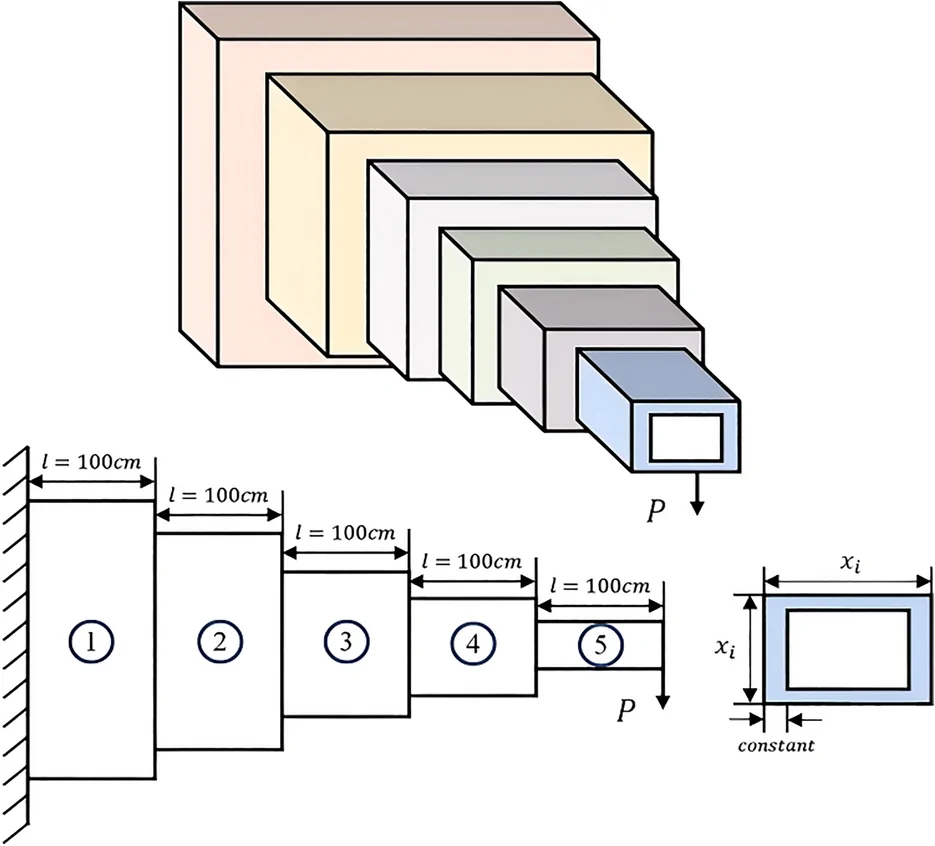
Illustration of the Cantilever Beam Design Problem.

**Fig. 35:**
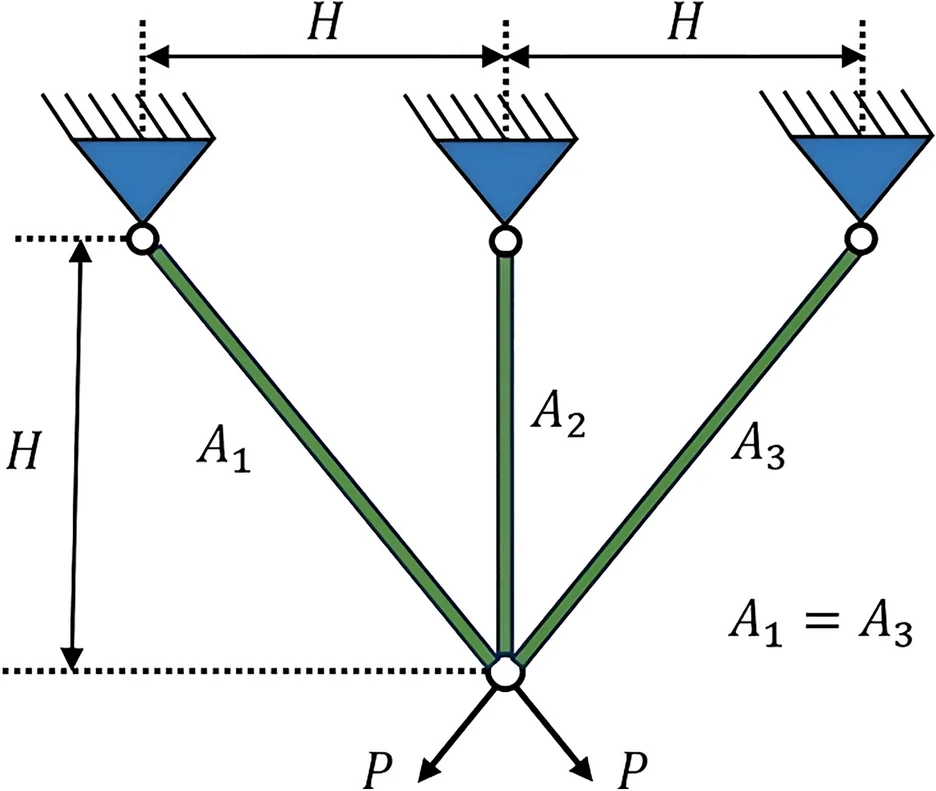
Illustration of the Three-Bar Truss Design Problem.

**Fig. 36:**
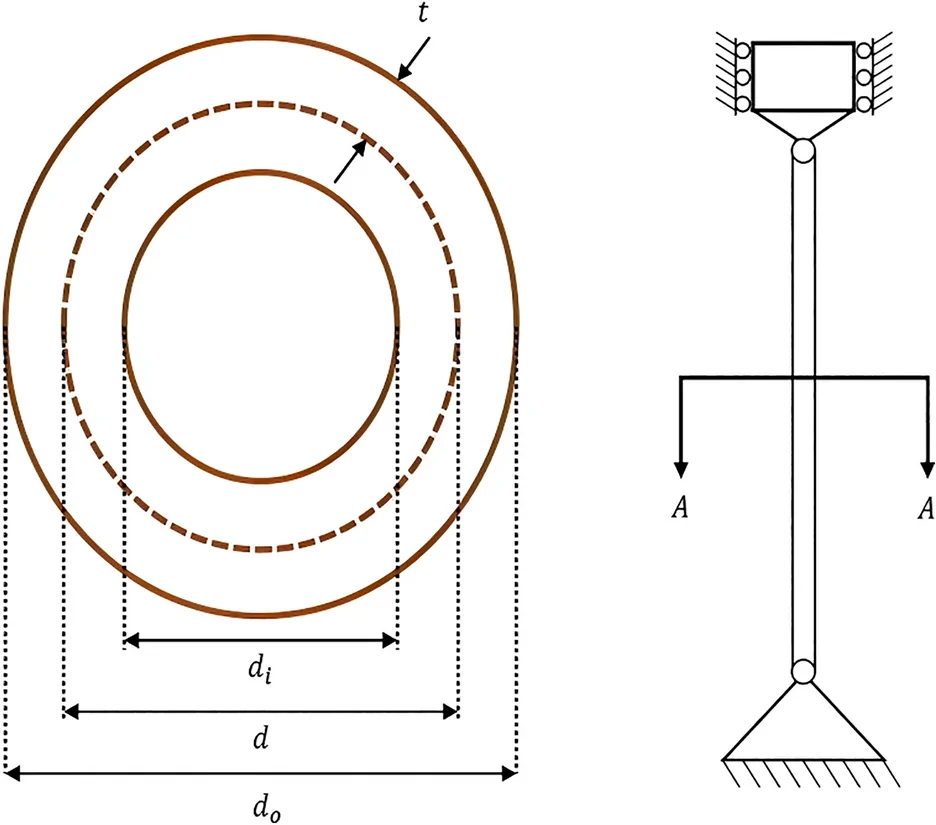
Illustration of the Tubular Column Design Problem.

**Fig. 37:**
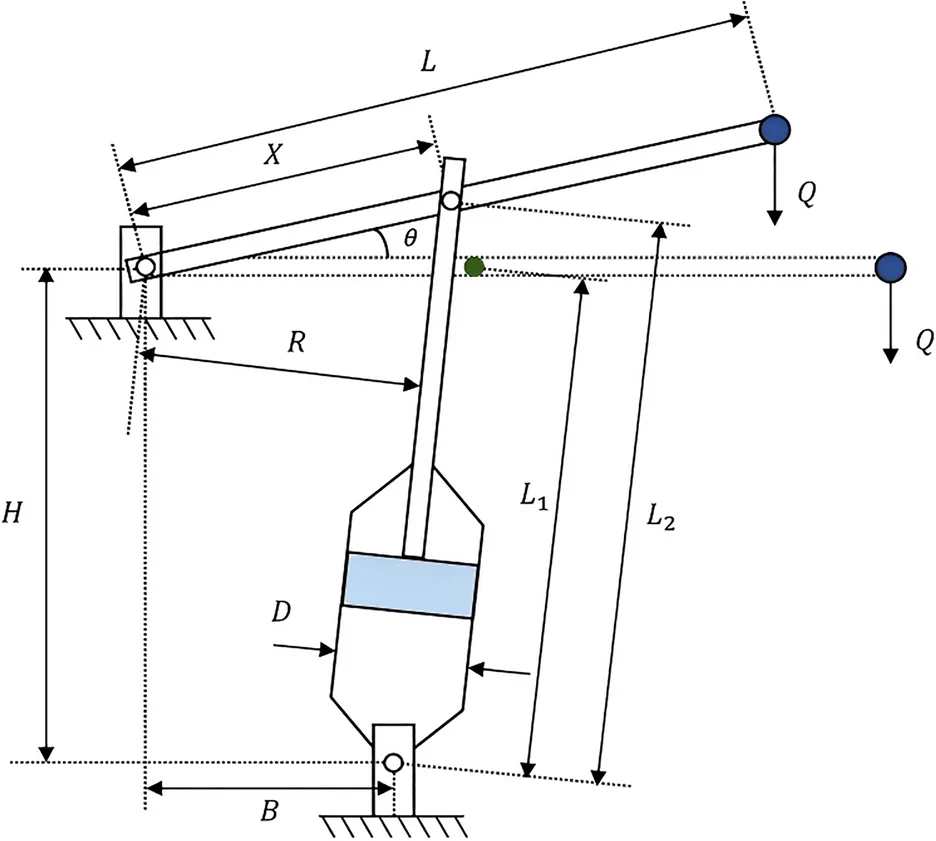
Illustration of the Piston Link Design Problem.

**Fig. 38:**
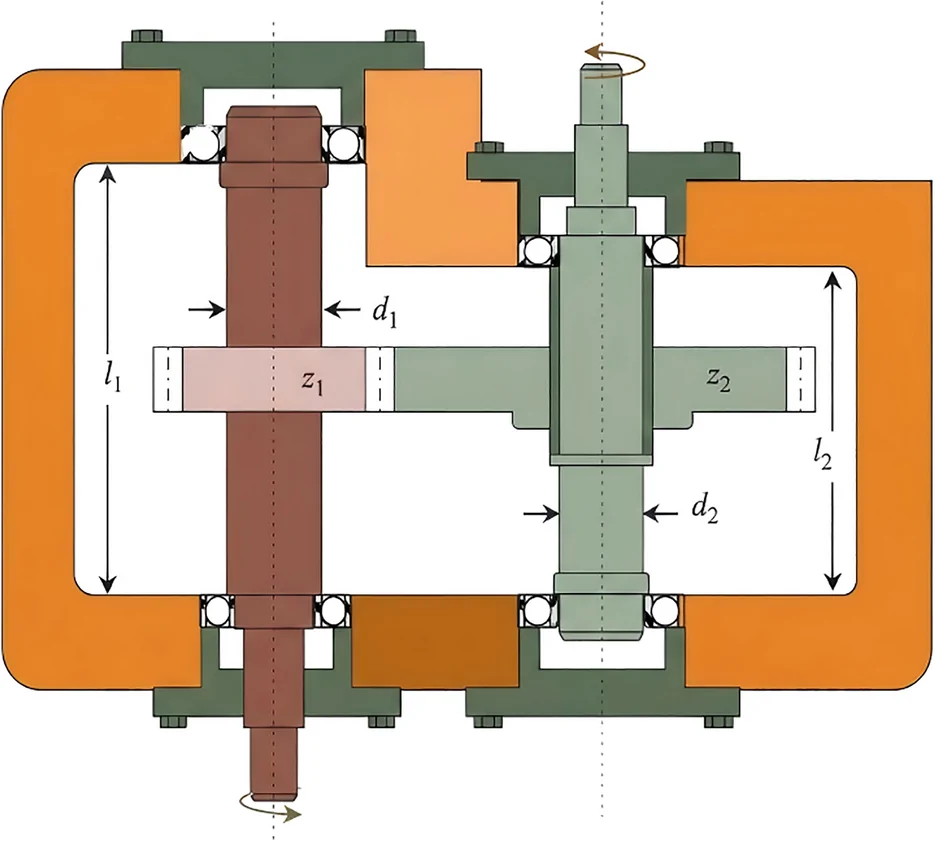
Illustration of the Weight Minimization Design Problem for Reducers.

## Data Availability

Data are contained within the article.

## A: Basic information about the CEC test set

Table 12 shows the basic information about the CEC2017 benchmark set. Where $\text {Uni.}$ refers to unimodal functions, $\text {Multi.}$ refers to simple multimodal functions, $\text {Hybrid.}$ refers to hybrid functions, and $\text {Comp.}$ refers to composite functions.

**Table 12:** Basic Information of CEC2017 Functions.

No.	Func.	Feature.	Optimum.
f1	Shifted and Rotated Bent Cigar function	Uni.	100
f3	Shifted and Rotated Rastrigin’s function		300
f4	Shifted and Rotated Expanded Scaffer’s F6 function		400
f5	Shifted and Rotated Lunacek Bi Rastrigin function		500
f6	Shifted and Rotated Lunacek Bi Rastrigin function	Multi.	600
f7	Shifted and Rotated Non-Continuous Rastrigin’s function		700
f8	Shifted and Rotated Levy function		800
f9	Shifted and Rotated Schwefel’s function		900
f10	Hybrid function 1(*N=3*)		1000
f11	Hybrid function 2(*N=3*)		1100
f12	Hybrid function 3(*N=3*)		1200
f13	Hybrid function 4(*N=4*)		1300
f14	Hybrid function 5(*N=4*)		1400
f15	Hybrid function 6(*N=4*)	Hybrid.	1500
f16	Hybrid function 6(*N=5*)		1600
f17	Hybrid function 6(*N=5*)		1700
f18	Hybrid function 6(*N=5*)		1800
f19	Hybrid function 6(*N=6*)		1900
f20	Composition function 1(*N=3*)		2000
f21	Composition function 2(*N=3*)		2100
f22	Composition function 3(*N=4*)		2200
f23	Composition function 4(*N=4*)		2300
f24	Composition function 5(*N=5*)		2400
f25	Composition function 6(*N=5*)	Comp.	2500
f26	Composition function 7(*N=6*)		2600
f27	Composition function 8(*N=6*)		2700
f28	Composition function 9(*N=3*)		2800
f29	Composition function 10(*N=3*)		2900
f30	Composition function 11(*N=3*)		3000

Table 13 shows the basic information about the CEC2022 benchmark set. Where $\text {Uni.}$ refers to unimodal functions, $\text {Basic.}$ refers to basic functions, and $\text {Hybrid.}$ refers to hybrid functions.

**Table 13:** Basic Information of CEC2022 Functions.

No.	Func.	Feature	Optimum
f1	Shifted and full Rotated Zakharov Function	Uni.	300
f2	Shifted and full Rotated Rosenbrock’s Function		400
f3	Shifted and full Rotated Expanded Scaffer’s f6. Function		600
f4	Shifted and full Rotated Non-Continuous Rastrigin’s Function	Basic.	800
f5	Shifted and full Rotated Levy Function		900
f6	Hybrid function 1(*N=3*)	Hybrid.	1800
f7	Hybrid function 2(*N=6*)		2000
f8	Hybrid function 3(*N=5*)		2200
f9	Composition function 1(*N=5*)		2300
f10	Composition function 2(*N=4*)		2400
f11	Composition function 3(*N=5*)	Comp.	2600
f12	Composition function 3(*N=6*)		2700

## B: Basic information about the CEC test set

Tables 14, 15, 16, 17, 18, 19, 20 and 21 summarize the experimental results of each dimension in CEC2017. Tables 22, 23 summarize the experimental results of each dimension in CEC2022. Tables 24, 25 summarize the average running time of each algorithm on the CEC2017 benchmark set with 30 dimensions and 50 dimensions.The best result among all compared algorithms is highlighted in bold.

**Table 14:** Detailed results of optimizers in 10-D CEC2017.

Func.	Metric	GA	PSO	HHO	DBO	POA	WOA	AO	TGCOA	ZOA	PO	MIPO	CGBPO	STPO
F1	std	6.66E+00	1.55E+00	9.27E+00	3.88E+00	5.48E+00	9.53E+00	5.12E+00	4.93E+00	2.94E+00	8.56E+00	1.00E+01	1.38E+00	2.08E-04
	mean	6.46E+02$^{+}$	6.31E+02$^{+}$	6.23E+02$^{+}$	6.15E+02$^{+}$	6.09E+02$^{+}$	6.29E+02$^{+}$	6.11E+02$^{+}$	6.41E+02$^{+}$	6.12E+02$^{+}$	6.19E+02$^{+}$	6.18E+02$^{+}$	6.03E+02$^{+}$	**6.00E+02**
F3	std	9.39E+00	4.75E+00	1.66E+01	1.04E+01	1.93E+01	2.63E+01	1.34E+01	3.47E+01	8.72E+00	1.73E+01	1.07E+01	5.54E+00	2.48E+00
	mean	7.69E+02$^{+}$	7.64E+02$^{+}$	7.81E+02$^{+}$	7.67E+02$^{+}$	7.62E+02$^{+}$	7.71E+02$^{+}$	7.46E+02$^{+}$	7.93E+02$^{+}$	7.35E+02$^{+}$	7.65E+02$^{+}$	7.41E+02$^{+}$	7.41E+02$^{+}$	**7.18E+02**
F4	std	6.34E+00	6.18E+00	1.07E+01	4.19E+00	6.59E+00	1.29E+01	7.67E+00	1.49E+01	5.32E+00	9.00E+00	8.95E+00	3.96E+00	4.71E+00
	mean	8.60E+02$^{+}$	8.36E+02$^{+}$	8.31E+02$^{+}$	8.28E+02$^{+}$	8.23E+02$^{+}$	8.34E+02$^{+}$	8.27E+02$^{+}$	8.45E+02$^{+}$	8.12E+02$^{=}$	8.27E+02$^{+}$	8.16E+02$^{=}$	8.17E+02$^{+}$	**8.09E+02**
F5	std	3.28E+00	3.71E-02	2.56E+02	2.31E+01	9.19E+01	3.46E+02	6.36E+01	3.48E+02	6.04E+01	1.14E+02	1.60E+02	8.64E-01	3.15E-02
	mean	9.11E+02$^{+}$	9.04E+02$^{+}$	1.28E+03$^{+}$	9.73E+02$^{+}$	9.63E+02$^{+}$	1.27E+03$^{+}$	9.54E+02$^{+}$	1.72E+03$^{+}$	9.67E+02$^{+}$	1.02E+03$^{+}$	1.03E+03$^{+}$	9.03E+02$^{+}$	**9.00E+02**
F6	std	2.82E+02	3.00E+02	3.30E+02	2.84E+02	2.35E+02	3.04E+02	3.05E+02	2.50E+02	1.80E+02	2.66E+02	3.84E+02	2.54E+02	2.93E+02
	mean	1.89E+03$^{+}$	1.74E+03$^{+}$	1.76E+03$^{+}$	1.90E+03$^{+}$	1.62E+03$^{+}$	1.91E+03$^{+}$	1.82E+03$^{+}$	2.29E+03$^{+}$	1.48E+03$^{=}$	1.80E+03$^{+}$	1.96E+03$^{+}$	1.80E+03$^{+}$	**1.42E+03**
F7	std	1.89E+03	2.58E+01	7.35E+01	3.76E+01	2.00E+01	7.82E+01	2.54E+01	5.56E+01	4.16E+01	3.12E+01	3.41E+01	2.02E+01	3.14E+00
	mean	2.31E+03$^{+}$	2.31E+03$^{+}$	1.18E+03$^{+}$	1.18E+03$^{+}$	1.13E+03$^{+}$	1.19E+03$^{+}$	1.15E+03$^{+}$	1.21E+03$^{+}$	1.15E+03$^{+}$	1.15E+03$^{+}$	1.15E+03$^{+}$	1.15E+03$^{+}$	**1.10E+03**
F8	std	2.72E+06	1.84E+04	1.39E+06	2.11E+06	7.04E+03	5.36E+06	2.97E+06	2.33E+06	1.07E+06	7.99E+05	5.03E+05	1.09E+06	1.11E+04
	mean	2.49E+06$^{+}$	1.90E+04$^{=}$	1.39E+06$^{+}$	1.52E+06$^{+}$	1.51E+04$^{=}$	5.98E+06$^{+}$	2.95E+06$^{+}$	2.60E+06$^{+}$	1.41E+06$^{+}$	7.59E+05$^{+}$	5.75E+05$^{+}$	1.36E+06$^{+}$	**1.05E+04**
F9	std	9.09E+03	3.54E+03	9.92E+03	1.36E+04	3.12E+02	1.93E+04	1.59E+04	8.10E+03	8.75E+03	1.18E+04	6.62E+03	5.65E+03	6.81E+03
	mean	1.55E+04$^{+}$	1.20E+04$^{=}$	1.22E+04$^{=}$	1.33E+04$^{+}$	1.82E+04$^{+}$	1.85E+04$^{=}$	1.75E+04$^{+}$	1.28E+04$^{=}$	1.29E+04$^{=}$	1.67E+04$^{+}$	9.04E+03$^{=}$	9.24E+03$^{=}$	**7.55E+03**
F10	std	5.99E+03	7.80E+01	2.03E+01	5.89E+02	1.20E+01	3.72E+01	1.43E+02	1.23E+02	9.84E+02	4.54E+01	1.57E+03	2.91E+01	2.59E+01
	mean	7.80E+03$^{+}$	2.48E+03$^{+}$	1.51E+03$^{+}$	1.82E+03$^{+}$	**1.44E+03**$^{=}$	1.53E+03$^{+}$	1.66E+03$^{+}$	1.55E+03$^{+}$	2.03E+03$^{+}$	1.51E+03$^{+}$	3.04E+03$^{+}$	1.49E+03$^{+}$	1.45E+03
F11	std	1.00E+04	2.74E+02	1.04E+02	7.80E+02	3.59E+01	1.91E+03	1.53E+03	5.26E+03	5.46E+02	1.06E+02	3.30E+03	1.10E+02	4.59E+01
	mean	1.17E+04$^{+}$	5.73E+03$^{+}$	1.70E+03$^{+}$	2.38E+03$^{+}$	1.56E+03$^{+}$	3.23E+03$^{+}$	3.69E+03$^{+}$	6.91E+03$^{+}$	2.22E+03$^{+}$	1.64E+03$^{+}$	4.96E+03$^{+}$	1.79E+03$^{+}$	**1.56E+03**
F12	std	7.28E+01	1.54E+02	1.34E+02	2.34E+01	7.94E+01	1.32E+02	6.68E+01	1.93E+02	1.01E+02	8.37E+01	1.13E+02	4.17E+01	1.39E+02
	mean	1.74E+03$^{+}$	1.92E+03$^{+}$	1.84E+03$^{+}$	1.73E+03$^{+}$	1.68E+03$^{=}$	1.87E+03$^{+}$	1.66E+03$^{=}$	2.04E+03$^{+}$	1.75E+03$^{+}$	1.72E+03$^{=}$	1.85E+03$^{+}$	**1.65E+03**$^{=}$	1.72E+03
F13	std	6.66E+00	1.55E+00	9.27E+00	3.88E+00	5.48E+00	9.53E+00	5.12E+00	4.93E+00	2.94E+00	8.56E+00	1.00E+01	1.38E+00	2.08E-04
	mean	6.46E+02$^{+}$	6.31E+02$^{+}$	6.23E+02$^{+}$	6.15E+02$^{+}$	6.09E+02$^{+}$	6.29E+02$^{+}$	6.11E+02$^{+}$	6.41E+02$^{+}$	6.12E+02$^{+}$	6.19E+02$^{+}$	6.18E+02$^{+}$	6.03E+02$^{+}$	**6.00E+02**
F14	std	9.39E+00	4.75E+00	1.66E+01	1.04E+01	1.93E+01	2.63E+01	1.34E+01	3.47E+01	8.72E+00	1.73E+01	1.07E+01	5.54E+00	2.48E+00
	mean	7.69E+02$^{+}$	7.64E+02$^{+}$	7.81E+02$^{+}$	7.67E+02$^{+}$	7.62E+02$^{+}$	7.71E+02$^{+}$	7.46E+02$^{+}$	7.93E+02$^{+}$	7.35E+02$^{+}$	7.65E+02$^{+}$	7.41E+02$^{+}$	7.41E+02$^{+}$	**7.18E+02**
F15	std	6.34E+00	6.18E+00	1.07E+01	4.19E+00	6.59E+00	1.29E+01	7.67E+00	1.49E+01	5.32E+00	9.00E+00	8.95E+00	3.96E+00	4.71E+00
	mean	8.60E+02$^{+}$	8.36E+02$^{+}$	8.31E+02$^{+}$	8.28E+02$^{+}$	8.23E+02$^{+}$	8.34E+02$^{+}$	8.27E+02$^{+}$	8.45E+02$^{+}$	8.12E+02$^{=}$	8.27E+02$^{+}$	8.16E+02$^{=}$	8.17E+02$^{+}$	**8.09E+02**
F16	std	3.28E+00	3.71E-02	2.56E+02	2.31E+01	9.19E+01	3.46E+02	6.36E+01	3.48E+02	6.04E+01	1.14E+02	1.60E+02	8.64E-01	3.15E-02
	mean	9.11E+02$^{+}$	9.04E+02$^{+}$	1.28E+03$^{+}$	9.73E+02$^{+}$	9.63E+02$^{+}$	1.27E+03$^{+}$	9.54E+02$^{+}$	1.72E+03$^{+}$	9.67E+02$^{+}$	1.02E+03$^{+}$	1.03E+03$^{+}$	9.03E+02$^{+}$	**9.00E+02**

**Table 15:** Detailed results of optimizers in 10-D CEC2017. (continued).

Func.	Metric	GA	PSO	HHO	DBO	POA	WOA	AO	TGCOA	ZOA	PO	MIPO	CGBPO	STPO
F17	std	3.62E+01	2.12E+01	3.83E+01	1.29E+01	9.40E+00	3.20E+01	2.75E+01	1.32E+02	9.49E+00	1.16E+01	3.08E+01	1.05E+01	1.82E+01
	mean	1.77E+03$^{+}$	**1.74E+03**$^{=}$	1.78E+03$^{+}$	1.77E+03$^{+}$	**1.74E+03**$^{=}$	1.79E+03$^{+}$	1.76E+03$^{+}$	1.90E+03$^{+}$	1.75E+03$^{=}$	1.76E+03$^{+}$	1.77E+03$^{+}$	1.76E+03$^{+}$	**1.74E+03**
F18	std	1.39E+04	8.99E+03	1.05E+04	1.31E+04	1.34E+02	1.36E+04	1.30E+04	3.69E+03	5.80E+03	1.55E+04	8.04E+03	1.27E+04	1.47E+03
	mean	2.09E+04$^{+}$	1.49E+04$^{+}$	1.27E+04$^{+}$	4.20E+04$^{+}$	2.97E+03$^{+}$	1.76E+04$^{+}$	2.13E+04$^{+}$	6.17E+03$^{+}$	7.34E+03$^{+}$	1.94E+04$^{+}$	1.05E+04$^{+}$	2.09E+04$^{+}$	**2.82E+03**
F19	std	9.89E+03	1.51E+03	8.51E+03	3.47E+02	1.41E+01	1.45E+04	1.98E+03	7.55E+03	2.81E+03	3.20E+03	3.26E+03	6.90E+01	3.75E+01
	mean	1.32E+04$^{+}$	7.16E+03$^{+}$	8.82E+03$^{+}$	2.32E+03$^{+}$	1.96E+03$^{+}$	2.01E+04$^{+}$	3.17E+03$^{+}$	6.50E+03$^{+}$	3.81E+03$^{+}$	2.85E+03$^{+}$	6.63E+03$^{+}$	2.00E+03$^{+}$	**1.94E+03**
F20	std	6.77E+01	4.14E+01	6.16E+01	2.08E+01	2.67E+01	7.35E+01	4.17E+01	6.65E+01	2.30E+01	4.65E+01	6.39E+01	1.57E+01	3.52E+01
	mean	2.11E+03$^{+}$	**1.46E+03**$^{+}$	2.15E+03$^{+}$	2.06E+03$^{+}$	2.06E+03$^{+}$	2.12E+03$^{+}$	2.09E+03$^{+}$	2.17E+03$^{+}$	2.05E+03$^{+}$	2.12E+03$^{+}$	2.11E+03$^{+}$	2.06E+03$^{+}$	2.02E+03
F21	std	3.49E+01	6.24E+01	7.05E+01	4.19E+00	4.45E+01	6.44E+01	5.89E+01	2.94E+01	5.82E+01	2.27E+00	3.27E+01	9.73E-01	4.57E+01
	mean	2.34E+03$^{+}$	2.38E+03$^{+}$	2.26E+03$^{=}$	2.21E+03$^{=}$	2.22E+03$^{=}$	2.29E+03$^{+}$	2.28E+03$^{+}$	2.34E+03$^{+}$	2.27E+03$^{+}$	**2.20E+03**$^{=}$	2.32E+03$^{+}$	**2.20E+03**$^{=}$	2.23E+03
F22	std	1.58E+01	3.18E+00	5.54E+00	4.13E+01	3.16E+01	2.67E+01	2.16E+01	6.35E+01	1.77E+01	2.89E+01	2.81E+01	8.73E-01	1.95E+01
	mean	2.35E+03$^{+}$	2.34E+03$^{+}$	2.32E+03$^{+}$	2.40E+03$^{+}$	2.31E+03$^{+}$	**2.30E+03**$^{=}$	**2.30E+03**$^{=}$	2.39E+03$^{+}$	2.32E+03$^{+}$	**2.30E+03**$^{=}$	2.33E+03$^{+}$	2.31E+03$^{+}$	**2.30E+03**
F23	std	1.20E+01	1.26E+01	1.67E+01	1.68E+01	8.71E+01	1.68E+01	1.37E+01	1.92E+01	1.81E+01	1.53E+01	2.60E+01	7.99E+00	8.75E+00
	mean	2.68E+03$^{+}$	2.69E+03$^{+}$	2.65E+03$^{+}$	2.65E+03$^{+}$	2.63E+03$^{+}$	2.65E+03$^{+}$	2.63E+03$^{+}$	2.68E+03$^{+}$	2.64E+03$^{+}$	2.64E+03$^{+}$	2.67E+03$^{+}$	2.63E+03$^{+}$	**2.62E+03**
F24	std	1.79E+01	9.06E+01	8.73E+01	5.92E+01	1.39E+02	7.82E+01	1.08E+02	7.03E+01	9.30E+01	1.38E+02	9.84E+01	1.10E+02	6.66E+01
	mean	2.82E+03$^{+}$	2.72E+03$^{=}$	2.78E+03$^{+}$	**2.56E+03**$^{-}$	2.63E+03$^{=}$	2.77E+03$^{+}$	2.71E+03$^{=}$	2.78E+03$^{+}$	2.73E+03$^{=}$	2.63E+03$^{=}$	2.74E+03$^{=}$	2.57E+03$^{-}$	2.74E+03
F25	std	1.14E+01	2.15E+01	1.99E+01	5.89E+01	7.71E+01	2.32E+01	3.39E+01	6.43E+01	2.86E+01	3.96E+01	2.24E+01	2.09E+01	1.97E+01
	mean	2.98E+03$^{+}$	2.98E+03$^{+}$	2.94E+03$^{=}$	2.97E+03$^{=}$	2.94E+03$^{+}$	2.94E+03$^{+}$	**2.93E+03**$^{=}$	2.98E+03$^{+}$	2.95E+03$^{=}$	2.94E+03$^{=}$	2.95E+03$^{+}$	**2.93E+03**$^{=}$	2.94E+03
F26	std	3.07E+02	7.18E+01	4.46E+02	6.37E+01	1.52E+02	4.87E+02	1.12E+02	4.61E+02	2.39E+02	1.71E+02	1.90E+02	4.41E+01	5.03E+01
	mean	3.14E+03$^{+}$	3.05E+03$^{+}$	3.39E+03$^{+}$	3.12E+03$^{+}$	2.96E+03$^{+}$	3.23E+03$^{+}$	3.05E+03$^{+}$	3.49E+03$^{+}$	3.05E+03$^{+}$	3.12E+03$^{+}$	3.11E+03$^{+}$	2.93E+03$^{+}$	**2.91E+03**
F27	std	1.60E+01	3.45E+01	3.34E+01	4.16E+00	4.62E+00	2.46E+01	2.36E+00	3.09E+01	2.98E+01	2.90E+00	3.98E+01	2.35E+00	3.87E+00
	mean	3.17E+03$^{+}$	3.12E+03$^{+}$	3.13E+03$^{+}$	3.10E+03$^{+}$	3.10E+03$^{=}$	3.12E+03$^{+}$	3.10E+03$^{+}$	3.16E+03$^{+}$	3.15E+03$^{+}$	3.10E+03$^{+}$	3.15E+03$^{+}$	**3.09E+03**$^{=}$	**3.09E+03**
F28	std	1.45E+01	1.67E+02	1.17E+02	9.01E+01	6.56E+01	1.13E+02	9.39E+01	1.12E+02	1.38E+02	1.01E+02	1.80E+02	1.25E+02	1.44E+02
	mean	3.23E+03$^{=}$	3.34E+03$^{+}$	**3.22E+03**$^{=}$	3.25E+03$^{=}$	3.29E+03$^{+}$	3.34E+03$^{=}$	3.35E+03$^{+}$	3.39E+03$^{+}$	3.35E+03$^{+}$	3.26E+03$^{=}$	3.34E+03$^{=}$	3.27E+03$^{=}$	3.25E+03
F29	std	4.70E+01	7.08E+01	5.12E+01	2.91E+01	2.37E+01	9.54E+01	2.98E+01	1.03E+02	2.35E+01	2.45E+01	3.63E+01	1.71E+01	4.29E+01
	mean	3.27E+03$^{+}$	3.24E+03$^{+}$	3.32E+03$^{+}$	3.22E+03$^{+}$	3.19E+03$^{+}$	3.34E+03$^{+}$	3.20E+03$^{=}$	3.35E+03$^{+}$	3.19E+03$^{=}$	3.22E+03$^{+}$	3.23E+03$^{+}$	**3.18E+03**$^{=}$	**3.18E+03**
F30	std	1.34E+06	5.10E+05	7.07E+05	7.54E+05	2.77E+04	4.54E+05	6.27E+05	3.40E+06	9.50E+05	4.72E+05	9.90E+05	3.92E+05	4.66E+05
	mean	8.29E+05$^{=}$	7.32E+05$^{+}$	4.79E+05$^{=}$	6.79E+05$^{=}$	2.55E+05$^{+}$	3.45E+05$^{=}$	3.33E+05$^{+}$	4.22E+06$^{+}$	7.37E+05$^{+}$	3.24E+05$^{=}$	6.76E+05$^{=}$	2.87E+05$^{=}$	**2.26E+05**
+/=/-:		27/3/0	25/5/0	25/5/0	25/4/1	23/7/0	26/4/0	25/5/0	29/1/0	23/7/0	22/8/0	24/6/0	21/8/1	*∼*
Avg. rank:		10.72	7.55	7.62	8.10	4.07	9.38	5.96	11.41	6.48	5.93	8.10	3.92	1.76

**Table 16:** Detailed results of optimizers in 30-D CEC2017.

Func.	Metric	GA	PSO	HHO	DBO	POA	WOA	AO	TGCOA	ZOA	PO	MIPO	CGBPO	STPO
F1	std	1.19E+07	5.35E+08	3.22E+06	2.43E+09	4.41E+09	3.62E+07	5.41E+06	5.09E+09	2.93E+09	7.23E+08	3.29E+09	4.70E+07	3.84E+03
	mean	1.96E+07$^{+}$	4.21E+08$^{+}$	1.27E+07$^{+}$	2.05E+10$^{+}$	1.12E+10$^{+}$	1.02E+08$^{+}$	1.78E+07$^{+}$	2.01E+10$^{+}$	6.27E+09$^{+}$	6.42E+08$^{+}$	8.40E+09$^{+}$	2.91E+08$^{+}$	**3.31E+03**
F3	std	4.55E+04	5.30E+03	3.41E+03	6.26E+03	8.23E+03	6.46E+04	5.02E+03	1.33E+04	5.74E+03	6.33E+03	1.31E+04	2.95E+03	4.20E+03
	mean	2.53E+05$^{+}$	**7.09E+03**$^{-}$	1.32E+04$^{=}$	6.30E+04$^{+}$	2.86E+04$^{+}$	2.30E+05$^{+}$	2.91E+04$^{+}$	7.86E+04$^{+}$	2.93E+04$^{+}$	1.46E+04$^{=}$	4.51E+04$^{+}$	9.87E+03$^{=}$	1.23E+04
F4	std	5.27E+01	1.34E+02	3.24E+01	8.05E+02	6.73E+02	5.43E+01	3.92E+01	1.64E+03	4.90E+02	5.60E+01	9.71E+02	2.37E+01	3.46E+01
	mean	6.36E+02$^{+}$	5.75E+02$^{+}$	5.43E+02$^{+}$	4.02E+03$^{+}$	1.21E+03$^{+}$	5.92E+02$^{+}$	5.64E+02$^{+}$	4.71E+03$^{+}$	1.07E+03$^{+}$	5.65E+02$^{+}$	1.61E+03$^{+}$	5.44E+02$^{+}$	**4.82E+02**
F5	std	3.25E+01	3.85E+01	1.78E+01	2.84E+01	4.48E+01	3.66E+01	3.07E+01	4.93E+01	2.54E+01	4.32E+01	2.15E+01	2.70E+01	2.19E+01
	mean	7.96E+02$^{+}$	7.88E+02$^{+}$	7.29E+02$^{+}$	7.84E+02$^{+}$	7.48E+02$^{+}$	7.93E+02$^{+}$	6.65E+02$^{+}$	8.63E+02$^{+}$	6.61E+02$^{+}$	7.28E+02$^{+}$	6.87E+02$^{+}$	6.81E+02$^{+}$	**5.69E+02**
F6	std	1.22E+01	7.17E+00	4.05E+00	5.44E+00	8.98E+00	1.18E+01	6.83E+00	1.07E+01	5.32E+00	7.87E+00	5.95E+00	4.55E+00	3.53E+00
	mean	6.93E+02$^{+}$	6.89E+02$^{+}$	6.61E+02$^{+}$	6.60E+02$^{+}$	6.51E+02$^{+}$	6.72E+02$^{+}$	6.44E+02$^{+}$	6.84E+02$^{+}$	6.45E+02$^{+}$	6.60E+02$^{+}$	6.55E+02$^{+}$	6.20E+02$^{+}$	**6.02E+02**
F7	std	3.53E+01	3.43E+01	7.88E+01	2.98E+01	9.31E+01	8.83E+01	4.28E+01	1.03E+02	7.17E+01	6.10E+01	9.13E+01	1.51E+01	1.47E+01
	mean	1.09E+03$^{+}$	9.64E+02$^{+}$	1.23E+03$^{+}$	1.14E+03$^{+}$	1.23E+03$^{+}$	1.20E+03$^{+}$	1.02E+03$^{+}$	1.33E+03$^{+}$	1.07E+03$^{+}$	1.16E+03$^{+}$	1.17E+03$^{+}$	9.53E+02$^{+}$	**8.03E+02**
F8	std	3.11E+01	2.20E+01	2.21E+01	2.84E+01	2.28E+01	8.01E+01	2.57E+01	4.62E+01	1.77E+01	3.95E+01	2.47E+01	2.01E+01	1.36E+01
	mean	1.10E+03$^{+}$	1.01E+03$^{+}$	9.68E+02$^{+}$	1.02E+03$^{+}$	9.73E+02$^{+}$	1.02E+03$^{+}$	9.45E+02$^{+}$	1.08E+03$^{+}$	9.34E+02$^{+}$	9.83E+02$^{+}$	9.44E+02$^{+}$	9.70E+02$^{+}$	**8.66E+02**
F9	std	8.63E+02	1.04E+03	8.58E+02	7.93E+02	9.33E+02	3.03E+03	1.36E+03	3.70E+03	7.24E+02	7.44E+02	8.85E+02	1.06E+02	3.10E+02
	mean	1.92E+03$^{+}$	2.80E+03$^{+}$	6.66E+03$^{+}$	6.23E+03$^{+}$	4.99E+03$^{+}$	8.32E+03$^{+}$	5.55E+03$^{+}$	1.07E+04$^{+}$	3.72E+03$^{+}$	5.74E+03$^{+}$	4.21E+03$^{+}$	**1.22E+03**$^{=}$	1.25E+03
F10	std	8.17E+02	4.97E+02	8.38E+02	5.62E+02	4.50E+02	7.07E+02	6.47E+02	4.38E+02	5.42E+02	6.34E+02	5.95E+02	5.84E+02	9.61E+02
	mean	5.70E+03$^{=}$	4.97E+03$^{+}$	5.73E+03$^{+}$	7.21E+03$^{+}$	4.73E+03$^{=}$	6.12E+03$^{+}$	5.40E+03$^{+}$	8.09E+03$^{+}$	**4.57E+03**$^{=}$	6.11E+03$^{+}$	6.07E+03$^{+}$	7.11E+03$^{+}$	4.96E+03
F11	std	7.73E+03	5.41E+01	4.74E+01	4.89E+02	1.82E+02	1.48E+03	1.01E+02	1.25E+03	2.69E+02	8.89E+01	1.33E+03	4.82E+01	4.17E+01
	mean	9.83E+03$^{+}$	7.26E+03$^{+}$	1.27E+03$^{+}$	2.77E+03$^{+}$	1.51E+03$^{+}$	2.81E+03$^{+}$	1.42E+03$^{+}$	4.31E+03$^{+}$	1.64E+03$^{+}$	1.33E+03$^{+}$	2.99E+03$^{+}$	1.42E+03$^{+}$	**1.19E+03**
F12	std	1.34E+07	5.60E+07	6.39E+06	1.19E+09	5.17E+08	6.76E+07	1.63E+07	1.05E+09	2.29E+08	4.20E+07	4.10E+08	1.79E+07	1.44E+05
	mean	1.40E+07$^{+}$	2.11E+07$^{=}$	1.23E+07$^{+}$	2.50E+09$^{+}$	4.42E+08$^{+}$	8.02E+07$^{+}$	2.52E+07$^{+}$	2.20E+09$^{+}$	1.94E+08$^{+}$	4.14E+07$^{+}$	3.43E+08$^{+}$	5.71E+07$^{+}$	**1.38E+05**
F13	std	3.61E+05	1.14E+06	1.36E+05	8.44E+08	6.62E+06	1.00E+05	1.40E+05	6.25E+08	1.21E+07	6.63E+04	1.60E+08	3.58E+06	1.98E+04
	mean	4.17E+05$^{+}$	3.80E+05$^{+}$	2.98E+05$^{+}$	5.99E+08$^{+}$	2.44E+06$^{+}$	1.74E+05$^{+}$	3.30E+05$^{+}$	3.96E+08$^{+}$	3.18E+06$^{+}$	1.14E+05$^{+}$	6.21E+07$^{+}$	1.49E+07$^{+}$	**2.27E+04**
F14	std	4.15E+06	3.22E+04	6.86E+04	2.59E+05	2.52E+03	1.48E+06	1.72E+05	4.79E+05	2.98E+05	4.16E+04	3.64E+05	2.78E+04	1.92E+04
	mean	3.49E+06$^{+}$	3.20E+06$^{+}$	6.93E+04$^{+}$	2.10E+05$^{+}$	**2.90E+03**$^{-}$	1.10E+06$^{+}$	2.89E+05$^{+}$	3.53E+05$^{+}$	1.32E+05$^{+}$	5.94E+04$^{+}$	3.56E+05$^{+}$	3.78E+04$^{+}$	1.81E+04
F15	std	1.71E+05	1.18E+04	3.55E+04	8.02E+05	2.64E+04	7.80E+04	5.36E+04	3.61E+04	1.07E+05	4.15E+04	7.09E+05	8.25E+05	1.35E+04
	mean	1.25E+05$^{+}$	1.21E+05$^{+}$	6.20E+04$^{+}$	1.33E+06$^{+}$	3.30E+04$^{+}$	1.22E+05$^{+}$	8.44E+04$^{+}$	5.98E+04$^{+}$	7.17E+04$^{+}$	6.61E+04$^{+}$	6.00E+05$^{+}$	1.66E+06$^{+}$	**1.17E+04**
F16	std	2.14E+02	1.40E+02	2.29E+02	2.27E+02	2.08E+02	5.41E+02	3.04E+02	6.94E+02	2.68E+02	4.82E+02	3.67E+02	2.82E+02	2.79E+02
	mean	3.09E+03$^{+}$	2.81E+03$^{+}$	3.21E+03$^{+}$	3.45E+03$^{+}$	2.78E+03$^{+}$	3.56E+03$^{+}$	3.04E+03$^{+}$	4.69E+03$^{+}$	2.84E+03$^{+}$	3.11E+03$^{+}$	3.18E+03$^{+}$	3.04E+03$^{+}$	**2.47E+03**

**Table 17:** Detailed results of optimizers in 30-D CEC2017. (continued).

Func.	Metric	GA	PSO	HHO	DBO	POA	WOA	AO	TGCOA	ZOA	PO	MIPO	CGBPO	STPO
F17	std	2.30E+02	2.42E+02	3.45E+02	2.53E+02	1.86E+02	2.59E+02	3.05E+02	3.37E+02	2.09E+02	2.68E+02	2.56E+02	1.01E+02	1.56E+02
	mean	2.54E+03$^{+}$	2.17E+03$^{=}$	2.54E+03$^{+}$	2.58E+03$^{+}$	2.09E+03$^{=}$	2.66E+03$^{+}$	2.26E+03$^{+}$	3.17E+03$^{+}$	2.23E+03$^{+}$	2.31E+03$^{+}$	2.42E+03$^{+}$	**2.06E+03**$^{=}$	**2.06E+03**
F18	std	3.40E+06	1.64E+05	1.64E+06	3.09E+06	1.18E+05	2.94E+06	1.24E+06	1.88E+06	5.36E+05	7.33E+05	6.72E+06	3.10E+05	6.75E+04
	mean	3.36E+06$^{+}$	3.23E+06$^{+}$	1.54E+06$^{+}$	2.42E+06$^{+}$	1.21E+05$^{=}$	4.20E+06$^{+}$	1.59E+06$^{+}$	1.94E+06$^{+}$	4.41E+05$^{+}$	9.03E+05$^{+}$	2.74E+06$^{+}$	5.75E+05$^{+}$	**9.99E+04**
F19	std	3.45E+05	5.01E+04	2.28E+05	9.83E+07	3.23E+05	4.99E+06	4.37E+05	2.84E+06	1.37E+06	9.08E+05	5.30E+05	1.89E+06	7.20E+03
	mean	3.72E+05$^{+}$	2.70E+04$^{=}$	2.82E+05$^{+}$	1.15E+08$^{+}$	2.97E+05$^{+}$	6.65E+06$^{+}$	8.59E+05$^{+}$	3.14E+06$^{+}$	1.71E+06$^{+}$	1.72E+06$^{+}$	5.47E+05$^{+}$	3.90E+06$^{+}$	**1.01E+04**
F20	std	2.40E+02	1.46E+02	2.02E+02	1.23E+02	8.31E+01	2.39E+02	1.92E+02	1.03E+02	5.32E+01	1.77E+02	2.01E+02	1.16E+02	1.03E+02
	mean	2.76E+03$^{+}$	2.46E+03$^{+}$	2.68E+03$^{+}$	2.59E+03$^{+}$	2.36E+03$^{=}$	2.85E+03$^{+}$	2.52E+03$^{+}$	2.85E+03$^{+}$	2.35E+03$^{=}$	2.56E+03$^{+}$	2.64E+03$^{+}$	2.50E+03$^{+}$	**2.33E+03**
F21	std	4.66E+01	5.15E+01	6.34E+01	1.22E+02	7.55E+01	5.96E+01	3.31E+01	5.51E+01	3.31E+01	5.59E+01	3.86E+01	1.82E+01	1.18E+01
	mean	2.70E+03$^{+}$	2.63E+03$^{+}$	2.56E+03$^{+}$	2.44E+03$^{=}$	2.49E+03$^{+}$	2.55E+03$^{+}$	2.45E+03$^{+}$	2.65E+03$^{+}$	2.47E+03$^{+}$	2.51E+03$^{+}$	2.49E+03$^{+}$	2.47E+03$^{+}$	**2.35E+03**
F22	std	2.43E+03	2.02E+03	2.07E+03	4.03E+02	1.00E+03	1.99E+03	7.55E+00	1.17E+03	1.18E+03	2.15E+03	1.79E+03	9.07E+00	1.41E+00
	mean	4.91E+03$^{+}$	4.76E+03$^{+}$	6.19E+03$^{+}$	4.94E+03$^{+}$	3.89E+03$^{+}$	6.99E+03$^{+}$	2.33E+03$^{+}$	9.23E+03$^{+}$	4.20E+03$^{+}$	3.57E+03$^{+}$	4.75E+03$^{+}$	2.38E+03$^{+}$	**2.30E+03**
F23	std	4.24E+01	4.89E+01	8.41E+01	7.77E+01	6.81E+01	6.72E+01	6.08E+01	8.85E+01	6.99E+01	6.00E+01	1.26E+02	3.60E+01	3.59E+01
	mean	3.10E+03$^{+}$	3.09E+03$^{+}$	3.10E+03$^{+}$	3.08E+03$^{+}$	2.94E+03$^{+}$	3.09E+03$^{+}$	2.92E+03$^{+}$	3.32E+03$^{+}$	3.08E+03$^{+}$	2.96E+03$^{+}$	3.20E+03$^{+}$	2.89E+03$^{+}$	**2.72E+03**
F24	std	5.92E+01	6.25E+01	1.41E+02	1.06E+02	5.35E+01	9.13E+01	4.53E+01	1.15E+02	9.94E+01	7.92E+01	8.97E+01	3.72E+01	3.63E+01
	mean	3.30E+03$^{+}$	3.04E+03$^{+}$	3.39E+03$^{+}$	3.25E+03$^{+}$	3.13E+03$^{+}$	3.20E+03$^{+}$	3.03E+03$^{+}$	3.49E+03$^{+}$	3.31E+03$^{+}$	3.14E+03$^{+}$	3.33E+03$^{+}$	3.03E+03$^{+}$	**2.91E+03**
F25	std	1.59E+02	1.78E+01	2.34E+01	1.41E+02	1.78E+02	4.15E+01	2.16E+01	2.35E+02	7.79E+01	3.30E+01	7.78E+01	2.27E+01	1.72E+01
	mean	3.32E+03$^{+}$	3.21E+03$^{=}$	2.92E+03$^{+}$	3.55E+03$^{+}$	3.16E+03$^{+}$	3.01E+03$^{+}$	2.92E+03$^{+}$	3.71E+03$^{+}$	3.08E+03$^{+}$	2.99E+03$^{+}$	3.13E+03$^{+}$	2.94E+03$^{+}$	**2.90E+03**
F26	std	5.01E+02	1.28E+03	1.43E+03	5.16E+02	1.34E+03	1.67E+03	1.58E+03	1.88E+03	9.47E+02	1.13E+03	1.48E+03	3.96E+02	9.00E+02
	mean	6.08E+03$^{+}$	4.92E+03$^{=}$	7.01E+03$^{+}$	6.61E+03$^{+}$	6.91E+03$^{+}$	7.43E+03$^{+}$	4.90E+03$^{=}$	9.02E+03$^{+}$	7.41E+03$^{+}$	6.58E+03$^{+}$	7.25E+03$^{+}$	6.26E+03$^{+}$	**4.18E+03**
F27	std	7.85E+01	5.00E+01	5.55E+01	1.24E+02	5.20E+01	1.05E+02	3.16E+01	3.02E+02	1.47E+02	5.94E+01	2.09E+02	2.47E+01	2.11E+01
	mean	3.59E+03$^{+}$	3.49E+03$^{+}$	3.34E+03$^{+}$	3.52E+03$^{+}$	3.33E+03$^{+}$	3.39E+03$^{+}$	3.32E+03$^{+}$	4.05E+03$^{+}$	3.74E+03$^{+}$	3.33E+03$^{+}$	3.64E+03$^{+}$	3.26E+03$^{+}$	**3.24E+03**
F28	std	1.51E+02	3.44E+01	2.55E+01	3.49E+02	3.37E+02	5.27E+01	3.69E+01	3.68E+02	3.40E+02	4.82E+01	3.29E+02	1.80E+01	4.75E+01
	mean	3.73E+03$^{+}$	3.28E+03$^{+}$	3.28E+03$^{+}$	4.48E+03$^{+}$	3.77E+03$^{+}$	3.38E+03$^{+}$	3.33E+03$^{+}$	4.75E+03$^{+}$	3.73E+03$^{+}$	3.35E+03$^{+}$	3.87E+03$^{+}$	3.30E+03$^{+}$	**3.18E+03**
F29	std	3.13E+02	2.21E+02	4.28E+02	3.64E+02	2.76E+02	4.09E+02	2.23E+02	1.11E+03	2.79E+02	3.24E+02	3.74E+02	3.17E+02	1.80E+02
	mean	4.39E+03$^{+}$	4.38E+03$^{+}$	4.46E+03$^{+}$	4.55E+03$^{+}$	4.50E+03$^{+}$	4.90E+03$^{+}$	4.30E+03$^{+}$	5.78E+03$^{+}$	4.47E+03$^{+}$	4.78E+03$^{+}$	4.65E+03$^{+}$	4.31E+03$^{+}$	**3.73E+03**
F30	std	3.49E+06	3.96E+04	9.71E+05	3.16E+07	2.71E+06	1.19E+07	6.25E+06	1.02E+08	8.44E+06	6.86E+06	9.29E+06	6.00E+06	4.24E+03
	mean	2.95E+06$^{+}$	2.93E+06$^{+}$	2.04E+06$^{+}$	4.99E+07$^{+}$	5.09E+06$^{+}$	1.47E+07$^{+}$	8.95E+06$^{+}$	7.88E+07$^{+}$	1.20E+07$^{+}$	9.96E+06$^{+}$	8.65E+06$^{+}$	9.30E+06$^{+}$	**9.67E+03**
+/=/-:		29/1/0	24/5/1	29/1/0	29/1/0	25/4/1	30/0/0	29/1/0	30/0/0	28/2/0	29/1/0	30/0/0	27/3/0	*∼*
Avg. rank:		8.68	6.43	6.21	9.75	6.17	9.64	4.57	11.79	6.50	6.36	8.75	4.94	1.21

**Table 18:** Detailed results of optimizers in 50-D CEC2017.

Func.	Metric	GA	PSO	HHO	DBO	POA	WOA	AO	TGCOA	ZOA	PO	MIPO	CGBPO	STPO
F1	std	4.34E+09	2.11E+09	1.18E+07	4.98E+09	9.50E+09	3.16E+08	1.12E+08	9.37E+09	5.69E+09	1.95E+09	9.74E+09	1.18E+08	3.17E+03
	mean	9.69E+09$^{+}$	2.12E+09$^{+}$	8.04E+07$^{+}$	5.81E+10$^{+}$	4.00E+10$^{+}$	6.86E+08$^{+}$	2.84E+08$^{+}$	6.02E+10$^{+}$	1.97E+10$^{+}$	5.16E+09$^{+}$	4.37E+10$^{+}$	1.03E+09$^{+}$	**1.86E+03**
F3	std	1.13E+05	1.81E+04	1.24E+04	1.96E+04	1.51E+04	4.59E+04	1.62E+04	2.70E+04	8.27E+03	1.22E+04	1.44E+04	7.03E+03	1.74E+04
	mean	4.35E+05$^{+}$	4.25E+05$^{+}$	5.39E+04$^{-}$	1.61E+05$^{+}$	8.24E+04$^{=}$	1.68E+05$^{+}$	1.50E+05$^{+}$	1.94E+05$^{+}$	8.76E+04$^{+}$	5.38E+04$^{-}$	1.47E+05$^{+}$	**4.35E+04**$^{-}$	7.60E+04
F4	std	3.05E+02	1.72E+02	3.72E+01	1.86E+03	2.55E+03	1.26E+02	6.10E+01	2.57E+03	1.30E+03	3.62E+02	2.11E+03	4.77E+01	4.42E+01
	mean	1.46E+03$^{+}$	7.97E+02$^{+}$	6.70E+02$^{+}$	1.34E+04$^{+}$	6.21E+03$^{+}$	9.39E+02$^{+}$	7.87E+02$^{+}$	1.52E+04$^{+}$	3.09E+03$^{+}$	1.23E+03$^{+}$	5.72E+03$^{+}$	7.23E+02$^{+}$	**5.17E+02**
F5	std	6.83E+01	4.78E+01	3.96E+01	3.11E+01	3.08E+01	7.36E+01	3.07E+01	4.86E+01	4.10E+01	4.64E+01	6.12E+01	2.38E+01	1.83E+01
	mean	1.14E+03$^{+}$	1.08E+03$^{+}$	8.89E+02$^{+}$	1.04E+03$^{+}$	9.22E+02$^{+}$	9.44E+02$^{+}$	8.41E+02$^{+}$	1.10E+03$^{+}$	8.25E+02$^{+}$	9.31E+02$^{+}$	9.19E+02$^{+}$	8.61E+02$^{+}$	**6.58E+02**
F6	std	8.38E+00	7.94E+00	5.69E+00	6.22E+00	3.77E+00	1.04E+01	4.34E+00	8.47E+00	5.15E+00	4.49E+00	6.96E+00	6.53E+00	4.17E+00
	mean	7.20E+02$^{+}$	6.94E+02$^{+}$	6.71E+02$^{+}$	6.77E+02$^{+}$	6.69E+02$^{+}$	6.90E+02$^{+}$	6.63E+02$^{+}$	6.95E+02$^{+}$	6.58E+02$^{+}$	6.68E+02$^{+}$	6.69E+02$^{+}$	6.32E+02$^{+}$	**6.09E+02**
F7	std	1.69E+02	1.01E+02	6.14E+01	6.09E+01	6.17E+01	8.56E+01	8.04E+01	8.39E+01	1.09E+02	9.83E+01	1.30E+02	2.30E+01	5.81E+01
	mean	1.81E+03$^{+}$	1.11E+03$^{+}$	1.83E+03$^{+}$	1.66E+03$^{+}$	1.74E+03$^{+}$	1.75E+03$^{+}$	1.46E+03$^{+}$	1.89E+03$^{+}$	1.52E+03$^{+}$	1.71E+03$^{+}$	1.71E+03$^{+}$	1.22E+03$^{+}$	**9.44E+02**
F8	std	4.36E+01	3.53E+01	2.72E+01	3.41E+01	4.72E+01	7.47E+01	2.60E+01	6.29E+01	4.25E+01	5.55E+01	4.05E+01	4.47E+01	3.13E+01
	mean	1.38E+03$^{+}$	1.31E+03$^{+}$	1.17E+03$^{+}$	1.34E+03$^{+}$	1.24E+03$^{+}$	1.26E+03$^{+}$	1.15E+03$^{+}$	1.43E+03$^{+}$	1.12E+03$^{+}$	1.21E+03$^{+}$	1.25E+03$^{+}$	1.19E+03$^{+}$	**9.56E+02**
F9	std	6.33E+03	1.92E+03	2.93E+03	3.71E+03	1.41E+03	8.02E+03	4.45E+03	4.23E+03	2.85E+03	2.67E+03	5.23E+03	1.07E+03	1.45E+03
	mean	1.44E+04$^{+}$	1.42E+04$^{+}$	2.15E+04$^{+}$	2.89E+04$^{+}$	1.67E+04$^{+}$	2.89E+04$^{+}$	2.01E+04$^{+}$	3.03E+04$^{+}$	1.34E+04$^{+}$	1.67E+04$^{+}$	1.72E+04$^{+}$	4.64E+03$^{+}$	**3.45E+03**
F10	std	1.14E+03	1.44E+03	9.77E+02	1.23E+03	7.91E+02	1.05E+03	1.33E+03	6.75E+02	4.92E+02	1.23E+03	1.22E+03	8.57E+02	1.27E+03
	mean	1.19E+04$^{+}$	1.01E+04$^{=}$	8.79E+03$^{+}$	1.33E+04$^{+}$	8.54E+03$^{+}$	1.07E+04$^{+}$	8.77E+03$^{+}$	1.38E+04$^{+}$	8.32E+03$^{+}$	1.01E+04$^{+}$	1.12E+04$^{+}$	1.23E+04$^{+}$	**7.44E+03**
F11	std	1.42E+04	8.94E+01	1.07E+02	2.02E+03	1.32E+03	2.78E+02	1.23E+02	5.43E+03	1.26E+03	2.15E+02	3.81E+03	1.04E+02	4.58E+01
	mean	2.92E+04$^{+}$	2.41E+04$^{+}$	1.51E+03$^{+}$	9.67E+03$^{+}$	5.39E+03$^{+}$	2.24E+03$^{+}$	1.75E+03$^{+}$	1.90E+04$^{+}$	2.79E+03$^{+}$	2.01E+03$^{+}$	1.06E+04$^{+}$	1.96E+03$^{+}$	**1.27E+03**
F12	std	2.71E+08	1.00E+09	4.49E+07	4.67E+09	5.07E+09	2.01E+08	1.55E+08	1.03E+10	2.89E+09	3.91E+08	9.93E+09	1.69E+08	1.45E+06
	mean	3.54E+08$^{+}$	4.91E+08$^{+}$	9.66E+07$^{+}$	2.47E+10$^{+}$	7.10E+09$^{+}$	4.72E+08$^{+}$	2.22E+08$^{+}$	2.39E+10$^{+}$	3.86E+09$^{+}$	4.29E+08$^{+}$	1.28E+10$^{+}$	4.25E+08$^{+}$	**1.83E+06**
F13	std	2.06E+07	9.14E+08	1.28E+06	4.77E+09	2.66E+09	6.36E+06	2.24E+07	2.35E+09	1.19E+08	2.55E+07	2.73E+09	1.37E+07	1.04E+04
	mean	1.75E+07$^{+}$	3.70E+08$^{+}$	2.78E+06$^{+}$	6.95E+09$^{+}$	1.43E+09$^{+}$	7.47E+06$^{+}$	8.19E+06$^{+}$	3.58E+09$^{+}$	2.98E+08$^{+}$	1.16E+07$^{+}$	3.42E+09$^{+}$	7.43E+07$^{+}$	**1.07E+04**
F14	std	1.35E+07	2.41E+05	4.33E+05	4.19E+06	1.25E+05	9.80E+05	2.21E+06	8.14E+06	1.28E+06	3.40E+05	1.48E+06	2.05E+05	5.53E+04
	mean	1.60E+07$^{+}$	1.16E+07$^{+}$	6.91E+05$^{+}$	4.66E+06$^{+}$	1.45E+05$^{=}$	1.68E+06$^{+}$	2.54E+06$^{+}$	7.54E+06$^{+}$	1.06E+06$^{+}$	5.71E+05$^{+}$	1.89E+06$^{+}$	3.82E+05$^{+}$	**1.15E+05**
F15	std	2.99E+07	1.05E+04	2.15E+05	5.76E+08	1.32E+07	5.70E+05	2.77E+05	4.35E+08	2.48E+07	1.35E+06	2.22E+08	4.46E+06	3.98E+03
	mean	1.18E+07$^{+}$	1.06E+07$^{+}$	3.59E+05$^{+}$	6.61E+08$^{+}$	6.73E+06$^{+}$	4.85E+05$^{+}$	4.12E+05$^{+}$	3.46E+08$^{+}$	2.76E+07$^{+}$	6.50E+05$^{+}$	9.58E+07$^{+}$	2.18E+07$^{+}$	**8.29E+03**
F16	std	5.64E+02	4.77E+02	5.80E+02	5.75E+02	5.14E+02	7.16E+02	6.61E+02	1.49E+03	3.57E+02	4.72E+02	6.95E+02	4.62E+02	4.12E+02
	mean	4.56E+03$^{+}$	4.27E+03$^{=}$	4.35E+03$^{+}$	4.90E+03$^{+}$	3.78E+03$^{+}$	5.64E+03$^{+}$	4.23E+03$^{+}$	7.47E+03$^{+}$	3.68E+03$^{+}$	4.93E+03$^{+}$	4.74E+03$^{+}$	4.06E+03$^{+}$	**3.08E+03**

**Table 19:** Detailed results of optimizers in 50-D CEC2017. (continued).

Func.	Metric	GA	PSO	HHO	DBO	POA	WOA	AO	TGCOA	ZOA	PO	MIPO	CGBPO	STPO
F17	std	4.17E+02	2.49E+02	2.48E+02	3.16E+02	4.28E+02	5.65E+02	3.98E+02	7.43E+02	2.99E+02	6.40E+02	5.16E+02	3.42E+02	2.22E+02
	mean	3.67E+03$^{+}$	3.33E+03$^{+}$	3.72E+03$^{+}$	4.33E+03$^{+}$	3.23E+03$^{+}$	4.16E+03$^{+}$	3.68E+03$^{+}$	4.55E+03$^{+}$	3.32E+03$^{+}$	3.99E+03$^{+}$	3.69E+03$^{+}$	3.51E+03$^{+}$	**2.78E+03**
F18	std	2.24E+07	3.78E+05	3.11E+06	9.48E+06	6.69E+05	1.05E+07	3.42E+06	1.92E+07	9.38E+06	1.84E+06	7.80E+06	1.75E+06	5.85E+05
	mean	3.15E+07$^{+}$	**4.06E+05**$^{=}$	5.14E+06$^{+}$	1.05E+07$^{+}$	1.26E+06$^{+}$	1.06E+07$^{+}$	5.52E+06$^{+}$	2.50E+07$^{+}$	7.41E+06$^{+}$	2.68E+06$^{+}$	7.85E+06$^{+}$	3.03E+06$^{+}$	5.11E+05
F19	std	2.82E+06	9.75E+04	7.05E+05	1.09E+08	7.63E+07	3.05E+06	9.89E+05	6.07E+07	1.47E+07	2.16E+06	1.36E+07	3.06E+06	7.10E+03
	mean	3.98E+06$^{+}$	3.50E+06$^{=}$	7.02E+05$^{+}$	1.74E+08$^{+}$	2.34E+07$^{+}$	4.33E+06$^{+}$	1.49E+06$^{+}$	5.93E+07$^{+}$	8.38E+06$^{+}$	2.76E+06$^{+}$	1.62E+07$^{+}$	9.92E+06$^{+}$	**1.23E+04**
F20	std	3.10E+02	2.76E+02	2.05E+02	2.33E+02	1.59E+02	3.00E+02	2.42E+02	1.81E+02	1.61E+02	2.61E+02	3.27E+02	2.83E+02	2.54E+02
	mean	3.48E+03$^{+}$	3.11E+03$^{=}$	3.44E+03$^{+}$	3.58E+03$^{+}$	2.94E+03$^{=}$	3.88E+03$^{+}$	3.13E+03$^{=}$	4.09E+03$^{+}$	**2.83E+03**$^{=}$	3.43E+03$^{+}$	3.46E+03$^{+}$	3.34E+03$^{+}$	2.97E+03
F21	std	7.21E+01	3.49E+01	8.10E+01	4.42E+01	6.51E+01	1.06E+02	5.87E+01	9.90E+01	5.28E+01	8.77E+01	4.78E+01	2.79E+01	3.34E+01
	mean	3.09E+03$^{+}$	2.78E+03$^{+}$	2.83E+03$^{+}$	2.88E+03$^{+}$	2.76E+03$^{+}$	2.91E+03$^{+}$	2.65E+03$^{+}$	3.06E+03$^{+}$	2.67E+03$^{+}$	2.75E+03$^{+}$	2.79E+03$^{+}$	2.68E+03$^{+}$	**2.43E+03**
F22	std	2.42E+03	1.60E+03	7.67E+02	1.70E+03	1.03E+03	1.44E+03	2.76E+03	8.19E+02	1.21E+03	1.08E+03	1.04E+03	2.84E+03	3.29E+03
	mean	1.44E+04$^{+}$	1.30E+04$^{=}$	1.10E+04$^{+}$	1.53E+04$^{+}$	1.04E+04$^{=}$	1.20E+04$^{+}$	9.74E+03$^{+}$	1.51E+04$^{+}$	1.04E+04$^{=}$	1.17E+04$^{+}$	1.30E+04$^{+}$	1.37E+04$^{+}$	**9.04E+03**
F23	std	1.28E+02	7.49E+01	1.19E+02	1.06E+02	1.56E+02	1.35E+02	9.78E+01	2.07E+02	1.44E+02	1.29E+02	2.47E+02	6.26E+01	6.58E+01
	mean	3.63E+03$^{+}$	3.27E+03$^{+}$	3.75E+03$^{+}$	3.68E+03$^{+}$	3.43E+03$^{+}$	3.69E+03$^{+}$	3.34E+03$^{+}$	4.12E+03$^{+}$	3.66E+03$^{+}$	3.39E+03$^{+}$	4.05E+03$^{+}$	3.29E+03$^{+}$	**2.91E+03**
F24	std	1.30E+02	1.23E+02	2.42E+02	1.74E+02	1.09E+02	1.26E+02	1.17E+02	2.33E+02	1.04E+02	1.73E+02	1.62E+02	8.00E+01	5.53E+01
	mean	4.02E+03$^{+}$	3.45E+03$^{+}$	4.11E+03$^{+}$	3.94E+03$^{+}$	3.59E+03$^{+}$	3.81E+03$^{+}$	3.45E+03$^{+}$	4.48E+03$^{+}$	3.98E+03$^{+}$	3.57E+03$^{+}$	3.96E+03$^{+}$	3.45E+03$^{+}$	**3.10E+03**
F25	std	8.91E+02	4.68E+01	3.75E+01	8.51E+02	1.30E+03	9.08E+01	4.81E+01	8.40E+02	5.02E+02	1.89E+02	8.03E+02	4.80E+01	2.58E+01
	mean	5.32E+03$^{+}$	5.21E+03$^{+}$	3.16E+03$^{+}$	9.25E+03$^{+}$	5.70E+03$^{+}$	3.39E+03$^{+}$	3.24E+03$^{+}$	8.86E+03$^{+}$	4.73E+03$^{+}$	3.56E+03$^{+}$	5.89E+03$^{+}$	3.18E+03$^{+}$	**3.07E+03**
F26	std	8.75E+02	1.60E+03	1.58E+03	4.84E+02	9.74E+02	1.22E+03	2.40E+03	1.33E+03	7.63E+02	1.61E+03	9.83E+02	5.22E+02	1.49E+03
	mean	1.01E+04$^{+}$	7.95E+03$^{+}$	1.09E+04$^{+}$	1.26E+04$^{+}$	1.19E+04$^{+}$	1.27E+04$^{+}$	7.91E+03$^{+}$	1.48E+04$^{+}$	1.22E+04$^{+}$	1.21E+04$^{+}$	1.31E+04$^{+}$	9.47E+03$^{+}$	**5.20E+03**
F27	std	2.69E+02	2.33E+02	3.24E+02	2.25E+02	2.12E+02	3.21E+02	1.97E+02	4.54E+02	3.50E+02	2.96E+02	5.66E+02	1.41E+02	8.21E+01
	mean	4.56E+03$^{+}$	4.28E+03$^{+}$	4.11E+03$^{+}$	4.74E+03$^{+}$	3.96E+03$^{+}$	4.36E+03$^{+}$	3.95E+03$^{+}$	5.81E+03$^{+}$	5.27E+03$^{+}$	4.01E+03$^{+}$	5.30E+03$^{+}$	3.61E+03$^{+}$	**3.46E+03**
F28	std	1.17E+03	5.60E+02	6.33E+01	5.59E+02	6.66E+02	2.56E+02	1.61E+02	9.81E+02	5.31E+02	2.95E+02	6.12E+02	4.71E+01	3.59E+01
	mean	6.06E+03$^{+}$	5.76E+03$^{+}$	3.47E+03$^{+}$	7.26E+03$^{+}$	6.22E+03$^{+}$	4.03E+03$^{+}$	3.85E+03$^{+}$	8.68E+03$^{+}$	5.20E+03$^{+}$	4.18E+03$^{+}$	6.52E+03$^{+}$	3.41E+03$^{+}$	**3.33E+03**
F29	std	5.06E+02	2.16E+02	6.13E+02	1.17E+03	7.28E+02	1.35E+03	5.64E+02	1.67E+03	8.73E+02	6.95E+02	8.09E+02	4.75E+02	4.61E+02
	mean	5.58E+03$^{+}$	5.04E+03$^{+}$	5.71E+03$^{+}$	8.03E+03$^{+}$	6.20E+03$^{+}$	8.12E+03$^{+}$	5.91E+03$^{+}$	1.05E+04$^{+}$	7.26E+03$^{+}$	6.70E+03$^{+}$	7.09E+03$^{+}$	6.04E+03$^{+}$	**4.24E+03**
F30	std	4.91E+07	4.21E+06	9.31E+06	3.26E+08	6.21E+07	7.66E+07	1.61E+07	3.53E+08	6.17E+07	3.41E+07	7.58E+07	2.91E+07	3.75E+05
	mean	1.50E+08$^{+}$	7.65E+07$^{+}$	2.99E+07$^{+}$	7.97E+08$^{+}$	1.49E+08$^{+}$	1.62E+08$^{+}$	7.50E+07$^{+}$	6.18E+08$^{+}$	1.70E+08$^{+}$	1.41E+08$^{+}$	1.88E+08$^{+}$	1.45E+08$^{+}$	**1.12E+06**
+/=/-:		30/0/0	24/6/0	29/0/1	30/0/0	26/4/0	30/0/0	29/1/0	30/0/0	28/2/0	29/0/1	30/0/0	29/0/1	*∼*
Avg. rank:		9.14	6.48	5.38	10.90	6.48	8.34	4.21	12.41	6.28	6.07	9.38	4.72	1.21

**Table 20:** Detailed results of optimizers in 100-D CEC2017.

Func.	Metric	GA	PSO	HHO	DBO	POA	WOA	AO	TGCOA	ZOA	PO	MIPO	CGBPO	STPO
F1	std	3.09E+10	7.03E+09	1.56E+08	9.00E+09	1.67E+10	3.23E+09	1.87E+09	1.43E+10	1.67E+10	1.15E+10	1.88E+10	4.49E+08	4.33E+03
	mean	2.58E+11$^{+}$	2.14E+10$^{+}$	9.06E+08$^{+}$	2.00E+11$^{+}$	1.24E+11$^{+}$	1.49E+10$^{+}$	8.44E+09$^{+}$	2.06E+11$^{+}$	8.67E+10$^{+}$	4.75E+10$^{+}$	1.61E+11$^{+}$	5.45E+09$^{+}$	**6.39E+03**
F3	std	1.07E+05	4.07E+04	2.19E+04	3.94E+04	1.84E+04	1.09E+05	1.73E+04	8.03E+04	1.32E+04	1.69E+04	1.52E+04	2.06E+04	1.69E+04
	mean	9.34E+05$^{+}$	8.44E+05$^{+}$	2.30E+05$^{-}$	3.71E+05$^{+}$	2.41E+05$^{-}$	8.93E+05$^{+}$	3.16E+05$^{+}$	4.56E+05$^{+}$	2.69E+05$^{=}$	**2.18E+05**$^{-}$	3.22E+05$^{+}$	2.47E+05$^{-}$	2.67E+05
F4	std	9.05E+03	1.27E+03	1.38E+02	4.75E+03	5.23E+03	6.02E+02	3.14E+02	8.98E+03	1.87E+03	1.20E+03	4.69E+03	1.24E+02	6.13E+01
	mean	3.58E+04$^{+}$	3.35E+03$^{+}$	1.22E+03$^{+}$	4.54E+04$^{+}$	1.74E+04$^{+}$	3.60E+03$^{+}$	2.38E+03$^{+}$	5.13E+04$^{+}$	9.84E+03$^{+}$	4.86E+03$^{+}$	2.55E+04$^{+}$	1.32E+03$^{+}$	**7.33E+02**
F5	std	1.39E+02	9.37E+01	4.53E+01	5.58E+01	5.32E+01	9.66E+01	5.83E+01	8.21E+01	9.48E+01	6.47E+01	5.23E+01	7.75E+01	6.98E+01
	mean	2.29E+03$^{+}$	2.21E+03$^{+}$	1.50E+03$^{+}$	1.87E+03$^{+}$	1.55E+03$^{+}$	1.68E+03$^{+}$	1.45E+03$^{+}$	2.00E+03$^{+}$	1.39E+03$^{+}$	1.63E+03$^{+}$	1.71E+03$^{+}$	1.51E+03$^{+}$	**9.66E+02**
F6	std	7.66E+00	5.16E+00	3.27E+00	3.38E+00	4.09E+00	9.76E+00	4.10E+00	6.44E+00	5.27E+00	4.40E+00	4.68E+00	5.49E+00	5.50E+00
	mean	7.36E+02$^{+}$	7.28E+02$^{+}$	6.84E+02$^{+}$	6.95E+02$^{+}$	6.77E+02$^{+}$	6.88E+02$^{+}$	6.76E+02$^{+}$	7.02E+02$^{+}$	6.73E+02$^{+}$	6.82E+02$^{+}$	6.94E+02$^{+}$	6.51E+02$^{+}$	**6.27E+02**
F7	std	9.04E+02	2.35E+02	8.80E+01	4.09E+01	8.56E+01	1.95E+02	1.57E+02	1.51E+02	1.74E+02	9.92E+01	1.18E+02	4.82E+01	1.08E+02
	mean	6.80E+03$^{+}$	6.40E+03$^{+}$	3.66E+03$^{+}$	3.49E+03$^{+}$	3.42E+03$^{+}$	3.49E+03$^{+}$	3.08E+03$^{+}$	3.82E+03$^{+}$	3.09E+03$^{+}$	3.47E+03$^{+}$	3.56E+03$^{+}$	2.06E+03$^{+}$	**1.65E+03**
F8	std	1.24E+02	7.32E+01	6.77E+01	7.05E+01	8.15E+01	9.64E+01	8.07E+01	8.24E+01	8.02E+01	1.10E+02	5.16E+01	6.51E+01	5.58E+01
	mean	2.63E+03$^{+}$	2.46E+03$^{+}$	1.95E+03$^{+}$	2.26E+03$^{+}$	2.04E+03$^{+}$	2.10E+03$^{+}$	1.87E+03$^{+}$	2.46E+03$^{+}$	1.83E+03$^{+}$	2.06E+03$^{+}$	2.19E+03$^{+}$	1.85E+03$^{+}$	**1.26E+03**
F9	std	1.71E+04	3.68E+03	5.13E+03	6.39E+03	2.12E+03	1.58E+04	5.54E+03	8.61E+03	3.07E+03	5.04E+03	9.66E+03	3.66E+03	8.94E+03
	mean	7.25E+04$^{+}$	6.41E+04$^{+}$	4.93E+04$^{+}$	7.23E+04$^{+}$	3.54E+04$^{=}$	5.59E+04$^{+}$	5.33E+04$^{+}$	7.04E+04$^{+}$	3.96E+04$^{+}$	3.79E+04$^{=}$	5.48E+04$^{+}$	**2.06E+04**$^{-}$	3.33E+04
F10	std	9.39E+02	1.81E+03	1.84E+03	9.60E+02	1.29E+03	1.34E+03	2.08E+03	1.25E+03	1.35E+03	2.15E+03	2.13E+03	9.14E+02	6.95E+03
	mean	2.96E+04$^{+}$	2.19E+04$^{+}$	2.14E+04$^{=}$	3.09E+04$^{+}$	**1.90E+04**$^{-}$	2.56E+04$^{=}$	1.99E+04$^{-}$	3.05E+04$^{+}$	1.97E+04$^{-}$	2.29E+04$^{=}$	2.60E+04$^{=}$	2.92E+04$^{+}$	2.43E+04
F11	std	6.38E+04	3.18E+03	5.76E+03	2.42E+04	1.13E+04	4.41E+04	2.96E+04	3.00E+04	9.92E+03	7.20E+03	1.72E+04	2.57E+03	7.37E+03
	mean	2.37E+05$^{+}$	1.37E+05$^{+}$	1.47E+04$^{=}$	1.39E+05$^{+}$	5.69E+04$^{+}$	1.10E+05$^{+}$	1.42E+05$^{+}$	1.82E+05$^{+}$	5.15E+04$^{+}$	2.81E+04$^{+}$	1.12E+05$^{+}$	1.20E+04$^{=}$	**1.19E+04**
F12	std	1.13E+10	4.36E+09	2.75E+08	1.23E+10	1.88E+10	8.43E+08	4.41E+08	2.37E+10	7.45E+09	2.48E+09	1.82E+10	3.51E+08	5.67E+06
	mean	5.03E+10$^{+}$	4.86E+09$^{+}$	7.39E+08$^{+}$	1.03E+11$^{+}$	4.90E+10$^{+}$	2.59E+09$^{+}$	1.50E+09$^{+}$	1.07E+11$^{+}$	2.35E+10$^{+}$	5.37E+09$^{+}$	6.64E+10$^{+}$	2.10E+09$^{+}$	**1.29E+07**
F13	std	2.92E+09	5.43E+08	1.60E+06	4.25E+09	6.06E+09	1.28E+07	4.39E+06	4.30E+09	2.34E+09	6.21E+08	2.68E+09	2.62E+07	4.78E+03
	mean	3.32E+09$^{+}$	3.12E+09$^{+}$	8.29E+06$^{+}$	2.10E+10$^{+}$	1.11E+10$^{+}$	2.11E+07$^{+}$	1.15E+07$^{+}$	1.80E+10$^{+}$	3.81E+09$^{+}$	4.89E+08$^{+}$	1.26E+10$^{+}$	1.97E+08$^{+}$	**6.50E+03**
F14	std	2.77E+07	7.96E+05	1.26E+06	4.84E+06	3.26E+06	3.33E+06	3.09E+06	2.01E+07	3.31E+06	2.30E+06	5.96E+06	1.40E+06	5.25E+05
	mean	5.77E+07$^{+}$	**1.01E+06**$^{=}$	3.18E+06$^{+}$	1.61E+07$^{+}$	4.99E+06$^{+}$	7.52E+06$^{+}$	8.74E+06$^{+}$	3.27E+07$^{+}$	6.60E+06$^{+}$	4.73E+06$^{+}$	1.15E+07$^{+}$	4.48E+06$^{+}$	1.07E+06
F15	std	4.99E+07	3.08E+04	1.30E+06	1.76E+09	1.79E+09	3.04E+06	1.10E+06	2.28E+09	1.45E+09	8.36E+06	2.43E+09	9.11E+06	2.20E+03
	mean	6.23E+07$^{+}$	3.92E+07$^{+}$	2.12E+06$^{+}$	6.64E+09$^{+}$	2.59E+09$^{+}$	3.55E+06$^{+}$	2.11E+06$^{+}$	4.89E+09$^{+}$	1.02E+09$^{+}$	7.77E+06$^{+}$	3.57E+09$^{+}$	6.98E+07$^{+}$	**4.02E+03**
F16	std	1.14E+03	4.50E+02	7.24E+02	9.04E+02	1.55E+03	2.02E+03	1.00E+03	2.41E+03	9.62E+02	1.58E+03	2.12E+03	6.85E+02	4.38E+02
	mean	1.12E+04$^{+}$	6.37E+03$^{+}$	7.49E+03$^{+}$	1.40E+04$^{+}$	9.41E+03$^{+}$	1.29E+04$^{+}$	8.39E+03$^{+}$	1.92E+04$^{+}$	8.53E+03$^{+}$	1.05E+04$^{+}$	1.20E+04$^{+}$	9.45E+03$^{+}$	**5.53E+03**

**Table 21:** Detailed results of optimizers in 100-D CEC2017. (continued).

Func.	Metric	GA	PSO	HHO	DBO	POA	WOA	AO	TGCOA	ZOA	PO	MIPO	CGBPO	STPO
F17	std	8.84E+02	1.57E+03	7.25E+02	1.26E+04	3.86E+03	1.33E+03	6.94E+02	3.66E+05	2.81E+03	1.18E+03	9.37E+04	5.00E+02	4.62E+02
	mean	7.97E+03$^{+}$	6.10E+03$^{+}$	6.52E+03$^{+}$	1.84E+04$^{+}$	8.93E+03$^{+}$	7.99E+03$^{+}$	6.59E+03$^{+}$	2.59E+05$^{+}$	9.14E+03$^{+}$	7.76E+03$^{+}$	8.34E+04$^{+}$	7.14E+03$^{+}$	**4.56E+03**
F18	std	1.87E+07	6.36E+05	1.40E+06	1.36E+07	1.66E+06	4.56E+06	2.70E+06	3.80E+07	1.77E+06	2.21E+06	1.18E+07	1.28E+06	6.50E+05
	mean	3.08E+07$^{+}$	8.38E+06$^{+}$	3.99E+06$^{+}$	2.76E+07$^{+}$	4.07E+06$^{+}$	6.66E+06$^{+}$	6.99E+06$^{+}$	4.83E+07$^{+}$	4.46E+06$^{+}$	4.78E+06$^{+}$	1.47E+07$^{+}$	5.75E+06$^{+}$	**1.45E+06**
F19	std	1.12E+08	2.11E+08	3.70E+06	2.05E+09	1.99E+09	2.57E+07	6.69E+06	1.85E+09	9.91E+08	3.23E+07	2.13E+09	1.07E+07	2.26E+03
	mean	1.38E+08$^{+}$	1.32E+08$^{+}$	8.73E+06$^{+}$	4.69E+09$^{+}$	1.80E+09$^{+}$	4.63E+07$^{+}$	1.20E+07$^{+}$	6.09E+09$^{+}$	8.03E+08$^{+}$	3.02E+07$^{+}$	4.36E+09$^{+}$	8.05E+07$^{+}$	**4.41E+03**
F20	std	4.85E+02	4.76E+02	4.29E+02	4.73E+02	3.93E+02	5.25E+02	4.99E+02	5.96E+02	2.88E+02	4.58E+02	4.74E+02	4.32E+02	7.39E+02
	mean	6.79E+03$^{+}$	5.05E+03$^{=}$	6.05E+03$^{+}$	6.89E+03$^{+}$	5.03E+03$^{=}$	6.52E+03$^{+}$	5.52E+03$^{+}$	7.33E+03$^{+}$	4.94E+03$^{=}$	6.18E+03$^{+}$	6.85E+03$^{+}$	6.39E+03$^{+}$	**4.75E+03**
F21	std	2.34E+02	1.63E+02	1.63E+02	1.16E+02	1.34E+02	2.01E+02	2.72E+02	2.26E+02	1.56E+02	1.43E+02	1.94E+02	7.39E+01	8.34E+01
	mean	4.57E+03$^{+}$	4.33E+03$^{+}$	4.00E+03$^{+}$	4.14E+03$^{+}$	3.70E+03$^{+}$	4.10E+03$^{+}$	3.83E+03$^{+}$	4.53E+03$^{+}$	3.69E+03$^{+}$	3.69E+03$^{+}$	4.16E+03$^{+}$	3.53E+03$^{+}$	**2.78E+03**
F22	std	1.39E+03	1.70E+03	1.07E+03	3.36E+03	1.17E+03	1.26E+03	1.92E+03	1.57E+03	9.95E+02	1.59E+03	1.74E+03	9.99E+03	8.36E+03
	mean	3.25E+04$^{+}$	**1.92E+04**$^{=}$	2.41E+04$^{=}$	3.20E+04$^{+}$	2.29E+04$^{=}$	2.80E+04$^{+}$	2.37E+04$^{=}$	3.28E+04$^{+}$	2.34E+04$^{=}$	2.57E+04$^{=}$	2.91E+04$^{+}$	2.74E+04$^{=}$	2.29E+04
F23	std	2.27E+02	2.70E+02	1.88E+02	2.40E+02	2.04E+02	3.66E+02	2.76E+02	3.98E+02	2.32E+02	2.53E+02	8.30E+02	1.82E+02	6.92E+01
	mean	5.37E+03$^{+}$	5.28E+03$^{+}$	4.83E+03$^{+}$	5.19E+03$^{+}$	4.63E+03$^{+}$	5.07E+03$^{+}$	4.42E+03$^{+}$	6.23E+03$^{+}$	5.46E+03$^{+}$	4.48E+03$^{+}$	6.15E+03$^{+}$	4.52E+03$^{+}$	**3.41E+03**
F24	std	4.47E+02	5.30E+02	4.79E+02	9.01E+02	3.22E+02	4.03E+02	2.93E+02	1.08E+03	3.13E+02	3.12E+02	6.18E+02	2.59E+02	1.61E+02
	mean	7.39E+03$^{+}$	6.47E+03$^{+}$	6.52E+03$^{+}$	8.22E+03$^{+}$	5.93E+03$^{+}$	6.26E+03$^{+}$	5.45E+03$^{+}$	9.75E+03$^{+}$	7.31E+03$^{+}$	5.79E+03$^{+}$	7.80E+03$^{+}$	5.03E+03$^{+}$	**4.00E+03**
F25	std	3.77E+03	6.44E+02	9.01E+01	8.68E+02	1.72E+03	3.02E+02	2.58E+02	2.16E+03	1.51E+03	7.45E+02	2.13E+03	1.39E+02	5.15E+01
	mean	2.26E+04$^{+}$	2.19E+04$^{+}$	3.81E+03$^{+}$	1.80E+04$^{+}$	1.16E+04$^{+}$	5.15E+03$^{+}$	4.51E+03$^{+}$	2.08E+04$^{+}$	8.41E+03$^{+}$	6.01E+03$^{+}$	1.48E+04$^{+}$	3.99E+03$^{+}$	**3.36E+03**
F26	std	2.93E+03	2.56E+03	5.03E+03	3.47E+03	2.71E+03	3.35E+03	2.24E+03	3.30E+03	1.88E+03	2.38E+03	2.89E+03	1.30E+03	2.31E+03
	mean	3.22E+04$^{+}$	3.21E+04$^{+}$	2.54E+04$^{+}$	3.48E+04$^{+}$	3.49E+04$^{+}$	3.51E+04$^{+}$	2.63E+04$^{+}$	4.44E+04$^{+}$	3.38E+04$^{+}$	3.30E+04$^{+}$	3.54E+04$^{+}$	2.24E+04$^{+}$	**1.40E+04**
F27	std	5.19E+02	2.34E+02	2.12E+02	8.98E+02	4.50E+02	7.02E+02	3.67E+02	1.45E+03	6.80E+02	4.98E+02	2.14E+03	1.34E+02	8.51E+01
	mean	6.47E+03$^{+}$	4.08E+03$^{+}$	4.52E+03$^{+}$	7.47E+03$^{+}$	5.32E+03$^{+}$	5.16E+03$^{+}$	4.71E+03$^{+}$	9.96E+03$^{+}$	8.63E+03$^{+}$	4.84E+03$^{+}$	8.77E+03$^{+}$	3.89E+03$^{+}$	**3.67E+03**
F28	std	4.40E+03	1.31E+03	1.38E+02	1.57E+03	1.80E+03	5.83E+02	3.94E+02	2.81E+03	2.39E+03	8.19E+02	2.41E+03	9.60E+01	4.74E+01
	mean	2.28E+04$^{+}$	2.12E+04$^{+}$	4.11E+03$^{+}$	2.00E+04$^{+}$	1.54E+04$^{+}$	6.37E+03$^{+}$	5.83E+03$^{+}$	2.54E+04$^{+}$	1.28E+04$^{+}$	6.60E+03$^{+}$	1.97E+04$^{+}$	3.93E+03$^{+}$	**3.48E+03**
F29	std	7.68E+03	6.24E+02	7.94E+02	6.23E+03	4.26E+03	1.99E+03	8.52E+02	2.00E+04	1.99E+03	1.23E+03	4.21E+03	9.10E+02	3.94E+02
	mean	1.61E+04$^{+}$	8.57E+03$^{+}$	9.89E+03$^{+}$	2.70E+04$^{+}$	1.54E+04$^{+}$	1.58E+04$^{+}$	1.15E+04$^{+}$	3.85E+04$^{+}$	1.43E+04$^{+}$	1.45E+04$^{+}$	1.77E+04$^{+}$	1.14E+04$^{+}$	**6.53E+03**
F30	std	2.09E+09	5.09E+08	2.30E+07	3.30E+09	3.86E+09	3.81E+08	1.09E+08	3.92E+09	1.55E+09	2.84E+08	3.03E+09	1.07E+08	4.37E+03
	mean	2.69E+09$^{+}$	2.41E+09$^{+}$	5.41E+07$^{+}$	1.79E+10$^{+}$	7.05E+09$^{+}$	6.96E+08$^{+}$	2.26E+08$^{+}$	1.23E+10$^{+}$	2.99E+09$^{+}$	5.24E+08$^{+}$	1.06E+10$^{+}$	3.88E+08$^{+}$	**1.15E+04**
+/=/-:		30/0/0	27/3/0	26/3/1	30/0/0	25/3/2	29/1/0	28/1/1	30/0/0	26/3/1	26/3/1	29/1/0	26/2/2	*∼*
Avg. rank:		10.79	7.10	3.97	10.86	6.52	7.34	4.55	12.21	6.34	5.59	10.10	4.17	1.46

**Table 22:** Detailed results of optimizers in 10-D CEC2022.

Func.	Metric	GA	PSO	HHO	DBO	POA	WOA	AO	TGCOA	ZOA	PO	MIPO	CGBPO	STPO
F1	std	1.50E+04	2.63E-14	3.07E-01	9.54E+02	4.78E+01	6.14E+03	2.51E+01	1.48E+03	6.59E+01	3.16E+00	2.35E+03	8.24E+00	2.15E-14
	mean	2.71E+04$^{+}$	**3.00E+02**$^{=}$	3.01E+02$^{+}$	1.73E+03$^{+}$	3.67E+02$^{+}$	1.06E+04$^{+}$	3.27E+02$^{+}$	4.45E+03$^{+}$	4.02E+02$^{+}$	3.03E+02$^{+}$	3.01E+03$^{+}$	3.31E+02$^{+}$	**3.00E+02**
F2	std	2.82E+01	1.18E+01	2.65E+01	7.67E+01	3.16E+01	2.31E+01	2.11E+01	7.27E+01	3.07E+01	3.45E+01	3.06E+01	2.10E+01	3.22E+00
	mean	4.41E+02$^{+}$	4.09E+02$^{=}$	4.18E+02$^{=}$	5.19E+02$^{+}$	4.18E+02$^{=}$	4.14E+02$^{=}$	4.16E+02$^{+}$	4.76E+02$^{+}$	4.27E+02$^{=}$	4.22E+02$^{=}$	4.23E+02$^{=}$	4.12E+02$^{=}$	**4.06E+02**
F3	std	5.36E+00	1.57E+00	1.18E+01	2.88E+00	6.52E+00	1.37E+01	3.65E+00	1.23E+01	5.55E+00	7.27E+00	5.90E+00	2.35E+00	2.31E-06
	mean	6.51E+02$^{+}$	6.16E+02$^{+}$	6.23E+02$^{+}$	6.15E+02$^{+}$	6.10E+02$^{+}$	6.32E+02$^{+}$	6.08E+02$^{+}$	6.43E+02$^{+}$	6.12E+02$^{+}$	6.16E+02$^{+}$	6.12E+02$^{+}$	6.04E+02$^{+}$	**6.00E+02**
F4	std	1.06E+01	6.81E+00	8.78E+00	6.89E+00	3.50E+00	1.54E+01	6.14E+00	9.00E+00	3.08E+00	7.49E+00	4.81E+00	2.91E+00	3.70E+00
	mean	8.55E+02$^{+}$	8.56E+02$^{+}$	8.24E+02$^{+}$	8.22E+02$^{+}$	8.18E+02$^{+}$	8.33E+02$^{+}$	8.20E+02$^{+}$	8.33E+02$^{+}$	**8.09E+02**$^{=}$	8.26E+02$^{+}$	8.11E+02$^{=}$	8.15E+02$^{+}$	**8.09E+02**
F5	std	2.58E+00	6.96E-01	1.93E+02	1.79E+01	7.41E+01	2.88E+02	4.38E+01	3.20E+02	2.81E+01	1.98E+02	7.46E+01	8.78E-01	1.44E-01
	mean	9.07E+02$^{+}$	**9.00E+02**$^{-}$	1.26E+03$^{+}$	9.80E+02$^{+}$	9.56E+02$^{+}$	1.36E+03$^{+}$	9.66E+02$^{+}$	1.58E+03$^{+}$	9.47E+02$^{+}$	1.07E+03$^{+}$	1.00E+03$^{+}$	9.03E+02$^{+}$	**9.00E+02**
F6	std	1.26E+03	2.11E+03	2.61E+03	2.10E+05	4.65E+01	2.14E+03	4.12E+03	1.45E+03	1.14E+03	2.60E+03	8.45E+02	2.63E+04	1.61E+03
	mean	3.40E+03$^{=}$	3.75E+03$^{+}$	4.44E+03$^{=}$	8.98E+04$^{+}$	**1.89E+03**$^{-}$	4.10E+03$^{=}$	8.30E+03$^{+}$	3.50E+03$^{=}$	3.05E+03$^{=}$	5.23E+03$^{+}$	2.58E+03$^{=}$	5.73E+04$^{+}$	3.69E+03
F7	std	9.25E+00	8.19E+00	2.64E+01	1.10E+01	1.01E+01	2.38E+01	9.55E+00	2.86E+01	8.92E+00	1.11E+01	1.79E+01	4.15E+00	4.50E+00
	mean	2.06E+03$^{+}$	**2.02E+03**$^{=}$	2.05E+03$^{+}$	2.04E+03$^{+}$	**2.02E+03**$^{=}$	2.06E+03$^{+}$	2.03E+03$^{+}$	2.09E+03$^{+}$	2.03E+03$^{+}$	2.04E+03$^{+}$	2.04E+03$^{+}$	2.04E+03$^{+}$	**2.02E+03**
F8	std	4.97E+00	3.13E+01	1.08E+01	4.55E+00	8.38E+00	6.30E+00	1.40E+00	8.24E+01	5.90E+00	8.10E+00	2.97E+00	5.66E+00	8.11E+00
	mean	2.23E+03$^{+}$	2.43E+03$^{+}$	2.23E+03$^{+}$	2.23E+03$^{+}$	2.23E+03$^{+}$	2.23E+03$^{+}$	2.23E+03$^{+}$	2.29E+03$^{+}$	**2.22E+03**$^{-}$	2.23E+03$^{+}$	2.23E+03$^{+}$	2.23E+03$^{+}$	**2.22E+03**
F9	std	1.86E+01	3.24E+01	3.79E+01	3.20E+01	8.08E+00	5.15E+01	6.87E+00	3.29E+01	4.59E+01	3.79E+01	3.68E+01	1.49E-01	0.00E+00
	mean	2.63E+03$^{+}$	2.54E+03$^{+}$	2.54E+03$^{+}$	2.60E+03$^{+}$	**2.53E+03**$^{-}$	2.55E+03$^{+}$	2.54E+03$^{+}$	2.61E+03$^{+}$	2.60E+03$^{+}$	2.54E+03$^{+}$	2.59E+03$^{+}$	2.54E+03$^{+}$	**2.53E+03**
F10	std	4.57E+01	6.87E+01	6.82E+01	2.30E+00	2.95E+01	7.35E+01	6.03E+01	8.19E+01	5.79E+01	2.74E-01	6.16E+01	1.00E-01	5.16E+01
	mean	2.53E+03$^{=}$	2.57E+03$^{+}$	2.55E+03$^{+}$	**2.50E+03**$^{=}$	2.54E+03$^{+}$	2.56E+03$^{=}$	2.56E+03$^{=}$	2.58E+03$^{+}$	2.55E+03$^{+}$	**2.50E+03**$^{=}$	2.54E+03$^{+}$	**2.50E+03**$^{=}$	2.53E+03
F11	std	1.89E+02	1.11E+02	1.58E+02	2.69E+01	2.06E+02	4.13E+01	9.64E+01	2.75E+02	2.29E+02	9.64E+01	2.26E+02	4.77E+00	1.33E+02
	mean	3.04E+03$^{+}$	3.04E+03$^{+}$	2.82E+03$^{+}$	2.77E+03$^{=}$	2.75E+03$^{+}$	2.90E+03$^{+}$	2.68E+03$^{=}$	2.96E+03$^{+}$	2.90E+03$^{+}$	**2.66E+03**$^{=}$	3.09E+03$^{+}$	2.77E+03$^{=}$	2.74E+03
F12	std	1.86E+01	9.55E+00	4.00E+01	1.12E+01	2.30E+00	3.46E+01	1.39E+00	4.01E+01	1.80E+01	2.22E+00	3.45E+01	1.69E+00	2.45E+00
	mean	2.94E+03$^{+}$	2.87E+03$^{+}$	2.90E+03$^{+}$	2.87E+03$^{+}$	**2.86E+03**$^{=}$	2.89E+03$^{+}$	2.87E+03$^{+}$	2.93E+03$^{+}$	2.91E+03$^{+}$	**2.86E+03**$^{=}$	2.92E+03$^{+}$	**2.86E+03**$^{=}$	**2.86E+03**
+/=/-:		10/2/0	8/3/1	10/2/0	10/2/0	7/3/2	9/3/0	10/2/0	11/1/0	8/3/1	8/4/0	9/3/0	8/4/0	*∼*
Avg. rank:		10.00	6.92	8.00	8.17	4.17	9.50	5.42	11.25	6.25	6.17	7.75	5.25	2.15

**Table 23:** Detailed results of optimizers in 20-D CEC2022.

Func.	Metric	GA	PSO	HHO	DBO	POA	WOA	AO	TGCOA	ZOA	PO	MIPO	CGBPO	STPO
F1	std	1.34E+04	1.32E-08	3.08E+01	4.37E+03	2.21E+03	3.70E+03	6.55E+03	6.81E+03	3.80E+03	6.86E+02	4.17E+03	9.35E+01	4.93E+00
	mean	6.68E+04$^{+}$	**3.00E+02**$^{-}$	3.70E+02$^{+}$	2.08E+04$^{+}$	5.37E+03$^{+}$	6.85E+03$^{+}$	2.61E+04$^{+}$	2.37E+04$^{+}$	6.67E+03$^{+}$	9.29E+02$^{+}$	1.71E+04$^{+}$	6.67E+02$^{+}$	3.02E+02
F2	std	4.26E+01	2.07E+01	1.49E+01	4.71E+01	9.47E+01	3.65E+01	4.03E+01	2.29E+02	4.97E+01	4.96E+01	6.86E+01	1.20E+01	1.35E+01
	mean	5.30E+02$^{+}$	4.56E+02$^{+}$	4.60E+02$^{+}$	7.37E+02$^{+}$	5.84E+02$^{+}$	5.07E+02$^{+}$	4.88E+02$^{+}$	9.02E+02$^{+}$	5.17E+02$^{+}$	5.05E+02$^{+}$	5.74E+02$^{+}$	4.56E+02$^{+}$	**4.48E+02**
F3	std	1.37E+01	9.01E+00	9.13E+00	5.98E+00	1.13E+01	1.43E+01	6.50E+00	6.93E+00	5.10E+00	9.43E+00	7.98E+00	4.71E+00	1.32E-01
	mean	6.79E+02$^{+}$	6.72E+02$^{+}$	6.54E+02$^{+}$	6.42E+02$^{+}$	6.40E+02$^{+}$	6.59E+02$^{+}$	6.35E+02$^{+}$	6.66E+02$^{+}$	6.37E+02$^{+}$	6.38E+02$^{+}$	6.45E+02$^{+}$	6.12E+02$^{+}$	**6.00E+02**
F4	std	1.80E+01	1.81E+01	1.55E+01	1.96E+01	1.13E+01	2.82E+01	1.38E+01	1.90E+01	1.25E+01	2.14E+01	1.80E+01	1.14E+01	1.02E+01
	mean	9.75E+02$^{+}$	9.75E+02$^{+}$	8.85E+02$^{+}$	9.13E+02$^{+}$	8.81E+02$^{+}$	9.14E+02$^{+}$	8.69E+02$^{+}$	9.30E+02$^{+}$	8.52E+02$^{+}$	8.77E+02$^{+}$	8.64E+02$^{+}$	8.67E+02$^{+}$	**8.32E+02**
F5	std	1.61E+01	6.01E+02	2.73E+02	2.81E+02	3.48E+02	1.42E+03	3.78E+02	3.82E+02	1.84E+02	3.24E+02	2.83E+02	1.17E+01	3.35E+01
	mean	9.42E+02$^{+}$	1.53E+03$^{+}$	2.69E+03$^{+}$	2.20E+03$^{+}$	2.06E+03$^{+}$	3.55E+03$^{+}$	1.96E+03$^{+}$	3.10E+03$^{+}$	1.55E+03$^{+}$	2.24E+03$^{+}$	1.80E+03$^{+}$	9.40E+02$^{+}$	**9.23E+02**
F6	std	6.99E+03	3.10E+03	2.90E+04	1.27E+07	4.73E+04	2.07E+04	3.20E+04	3.00E+07	5.98E+06	4.88E+03	6.39E+06	1.52E+06	2.84E+03
	mean	9.53E+03$^{+}$	9.53E+03$^{+}$	4.99E+04$^{+}$	2.55E+07$^{+}$	1.82E+04$^{+}$	1.74E+04$^{+}$	5.91E+04$^{+}$	1.41E+07$^{+}$	2.22E+06$^{+}$	5.78E+03$^{=}$	2.00E+06$^{+}$	4.15E+06$^{+}$	**3.87E+03**
F7	std	3.74E+01	4.46E+01	4.44E+01	1.74E+01	3.06E+01	7.23E+01	3.23E+01	4.35E+01	1.58E+01	2.67E+01	4.84E+01	1.97E+01	1.46E+01
	mean	2.18E+03$^{+}$	2.08E+03$^{+}$	2.13E+03$^{+}$	2.13E+03$^{+}$	2.09E+03$^{+}$	2.21E+03$^{+}$	2.10E+03$^{+}$	2.21E+03$^{+}$	2.09E+03$^{+}$	2.13E+03$^{+}$	2.17E+03$^{+}$	2.09E+03$^{+}$	**2.04E+03**
F8	std	5.74E+01	5.96E+01	3.84E+01	5.63E+01	4.27E+01	4.77E+01	7.32E+00	1.99E+02	3.08E+01	3.00E+01	6.17E+01	3.18E+00	4.20E+01
	mean	2.28E+03$^{+}$	2.28E+03$^{+}$	2.26E+03$^{+}$	2.28E+03$^{+}$	2.25E+03$^{+}$	2.27E+03$^{+}$	**2.24E+03**$^{=}$	2.50E+03$^{+}$	**2.24E+03**$^{=}$	2.25E+03$^{+}$	2.28E+03$^{+}$	**2.24E+03**$^{=}$	**2.24E+03**
F9	std	2.68E+01	1.67E+01	2.76E+00	6.31E+01	1.88E+01	2.37E+01	1.02E+01	6.18E+01	4.00E+01	1.68E+01	3.78E+01	3.50E-01	3.45E-12
	mean	2.58E+03$^{+}$	2.49E+03$^{+}$	**2.48E+03**$^{-}$	2.59E+03$^{+}$	2.52E+03$^{+}$	2.50E+03$^{+}$	2.50E+03$^{+}$	2.68E+03$^{+}$	2.55E+03$^{+}$	2.50E+03$^{+}$	2.58E+03$^{+}$	**2.48E+03**$^{-}$	**2.48E+03**
F10	std	1.22E+03	6.27E+02	5.86E+02	2.20E+01	6.70E+02	9.62E+02	4.01E+02	1.60E+03	2.99E+02	2.66E-01	1.09E+03	9.07E+01	5.94E+02
	mean	4.13E+03$^{+}$	3.84E+03$^{+}$	3.00E+03$^{=}$	2.54E+03$^{=}$	2.79E+03$^{=}$	4.64E+03$^{+}$	2.60E+03$^{=}$	4.60E+03$^{+}$	2.62E+03$^{=}$	**2.50E+03**$^{=}$	3.74E+03$^{+}$	2.55E+03$^{=}$	2.86E+03
F11	std	2.58E+02	5.95E-13	1.26E+02	3.20E+02	7.18E+02	7.34E+01	1.64E+02	1.15E+03	6.62E+02	2.28E+02	1.10E+03	2.13E+02	4.55E-13
	mean	4.23E+03$^{+}$	4.20E+03$^{+}$	2.97E+03$^{+}$	5.67E+03$^{+}$	4.11E+03$^{+}$	3.01E+03$^{+}$	2.98E+03$^{+}$	5.93E+03$^{+}$	3.99E+03$^{+}$	3.37E+03$^{+}$	5.26E+03$^{+}$	4.25E+03$^{+}$	**2.90E+03**
F12	std	6.61E+01	4.41E+01	5.84E+01	6.17E+01	4.62E+01	6.97E+01	2.77E+01	1.59E+02	1.23E+02	1.46E+01	1.72E+02	1.38E+01	3.21E+01
	mean	3.32E+03$^{+}$	2.99E+03$^{=}$	3.04E+03$^{+}$	3.10E+03$^{+}$	3.00E+03$^{+}$	3.05E+03$^{+}$	2.99E+03$^{+}$	3.38E+03$^{+}$	3.33E+03$^{+}$	2.98E+03$^{+}$	3.22E+03$^{+}$	**2.96E+03**$^{=}$	**2.96E+03**
+/=/-:		12/0/0	10/1/1	10/1/1	11/1/0	11/1/0	12/0/0	10/2/0	12/0/0	10/2/0	10/2/0	12/0/0	8/3/1	*∼*
Avg. rank:		9.83	6.25	6.50	9.58	6.75	8.67	5.42	12.25	6.42	5.00	8.67	3.92	1.74

**Table 24:** Running time of each algorithm on 30-D CEC2017.

Func.	GA	PSO	HHO	DBO	POA	WOA	AO	TGCOA	ZOA	PO	MIPO	CGBPO	STPO
F1	6.366E-01	5.943E-01	1.147E+00	7.084E-01	5.569E-01	4.531E-01	1.051E+00	6.700E-01	5.794E-01	2.946E+01	9.425E-01	6.822E+00	7.357E-01
F3	1.645E+00	1.148E+00	3.362E+00	2.330E+00	1.843E+00	1.231E+00	1.566E+00	6.573E-01	5.948E-01	2.481E+01	9.100E-01	2.887E+00	1.965E+00
F4	5.902E-01	4.783E-01	1.047E+00	6.945E-01	5.537E-01	4.348E-01	1.031E+00	6.532E-01	5.796E-01	2.483E+01	9.095E-01	2.884E+00	6.825E-01
F5	7.828E-01	6.777E-01	1.555E+00	8.775E-01	9.490E-01	6.410E-01	1.432E+00	8.509E-01	9.722E-01	4.439E+01	1.291E+00	3.449E+00	9.431E-01
F6	1.272E+00	1.171E+00	2.698E+00	1.367E+00	1.884E+00	1.125E+00	2.397E+00	1.328E+00	1.923E+00	9.204E+01	2.201E+00	5.009E+00	1.383E+00
F7	7.878E-01	6.888E-01	1.582E+00	8.927E-01	9.983E-01	6.624E-01	1.484E+00	8.681E-01	1.007E+00	4.455E+01	1.324E+00	3.484E+00	9.137E-01
F8	7.913E-01	6.964E-01	1.597E+00	8.899E-01	9.688E-01	6.482E-01	1.447E+00	8.553E-01	9.991E-01	4.582E+01	1.309E+00	3.489E+00	9.579E-01
F9	7.965E-01	6.867E-01	1.609E+00	9.032E-01	9.723E-01	6.596E-01	1.488E+00	8.737E-01	1.009E+00	4.598E+01	1.321E+00	3.535E+00	8.919E-01
F10	9.198E-01	7.984E-01	1.916E+00	1.085E+00	1.174E+00	7.716E-01	1.678E+00	9.938E-01	1.243E+00	5.745E+01	1.509E+00	3.972E+00	1.011E+00
F11	6.742E-01	5.749E-01	1.301E+00	7.985E-01	7.288E-01	5.339E-01	1.214E+00	7.475E-01	7.700E-01	3.408E+01	1.083E+00	3.153E+00	7.807E-01
F12	7.777E-01	6.753E-01	1.517E+00	8.913E-01	9.164E-01	6.380E-01	1.411E+00	8.480E-01	9.594E-01	4.342E+01	1.260E+00	3.456E+00	8.694E-01
F13	6.977E-01	5.813E-01	1.319E+00	8.288E-01	7.615E-01	5.402E-01	1.246E+00	7.789E-01	7.918E-01	3.731E+01	1.208E+00	3.516E+00	7.876E-01
F14	1.006E+00	8.614E-01	2.011E+00	1.157E+00	1.288E+00	8.448E-01	1.807E+00	1.047E+00	1.307E+00	6.108E+01	1.679E+00	4.373E+00	1.073E+00
F15	7.241E-01	5.933E-01	1.350E+00	8.336E-01	7.350E-01	5.717E-01	1.288E+00	7.786E-01	7.594E-01	3.364E+01	1.124E+00	3.380E+00	8.003E-01
F16	8.122E-01	6.771E-01	1.542E+00	9.321E-01	9.096E-01	6.646E-01	1.476E+00	8.748E-01	9.468E-01	4.038E+01	1.185E+00	3.334E+00	8.917E-01
F17	1.065E+00	9.756E-01	2.255E+00	1.200E+00	1.533E+00	9.367E-01	2.021E+00	1.157E+00	1.561E+00	7.173E+01	1.777E+00	4.193E+00	1.199E+00
F18	7.159E-01	6.409E-01	1.453E+00	8.632E-01	8.572E-01	5.867E-01	1.327E+00	7.949E-01	8.832E-01	3.968E+01	1.181E+00	3.210E+00	8.086E-01
F19	3.325E+00	3.171E+00	7.660E+00	3.386E+00	5.920E+00	3.134E+00	6.396E+00	3.318E+00	5.962E+00	6.421E+02	1.567E+01	2.985E+01	3.340E+00
F20	3.339E+00	3.062E+00	7.698E+00	3.897E+00	5.402E+00	2.928E+00	5.770E+00	3.457E+00	5.604E+00	2.485E+02	6.426E+00	1.465E+01	3.741E+00
F21	3.247E+00	2.722E+00	7.593E+00	4.164E+00	5.116E+00	3.016E+00	6.721E+00	3.771E+00	5.355E+00	2.430E+02	6.056E+00	1.439E+01	3.701E+00
F22	3.502E+00	3.160E+00	7.877E+00	3.263E+00	2.441E+00	1.403E+00	2.944E+00	1.627E+00	2.506E+00	1.252E+02	2.761E+00	5.825E+00	3.938E+00
F23	1.736E+00	1.592E+00	3.675E+00	1.829E+00	2.717E+00	1.544E+00	3.164E+00	1.724E+00	2.750E+00	1.446E+02	2.943E+00	6.036E+00	1.777E+00
F24	1.905E+00	1.776E+00	4.168E+00	2.315E+00	3.093E+00	1.719E+00	3.489E+00	1.907E+00	3.110E+00	1.718E+02	3.308E+00	6.518E+00	2.053E+00
F25	1.430E+00	1.353E+00	2.987E+00	1.455E+00	2.204E+00	1.273E+00	2.605E+00	1.431E+00	2.243E+00	1.117E+02	2.507E+00	5.253E+00	1.554E+00
F26	1.922E+00	1.799E+00	4.251E+00	2.039E+00	3.241E+00	1.778E+00	3.679E+00	1.970E+00	3.244E+00	1.626E+02	3.470E+00	6.819E+00	2.036E+00
F27	2.137E+00	2.048E+00	4.706E+00	2.227E+00	3.563E+00	1.957E+00	4.054E+00	2.159E+00	3.631E+00	1.809E+02	3.838E+00	7.449E+00	2.193E+00
F28	1.786E+00	1.707E+00	3.815E+00	1.812E+00	2.868E+00	1.627E+00	3.287E+00	1.772E+00	2.927E+00	1.480E+02	3.202E+00	6.304E+00	1.793E+00
F29	1.447E+00	1.365E+00	3.210E+00	1.599E+00	2.347E+00	1.327E+00	2.807E+00	1.534E+00	2.360E+00	1.127E+02	2.587E+00	5.444E+00	1.557E+00
F30	3.740E+00	3.600E+00	8.549E+00	3.799E+00	6.734E+00	3.551E+00	7.241E+00	3.745E+00	6.757E+00	3.358E+02	6.862E+00	1.217E+01	3.755E+00

**Table 25:** Running time of each algorithm on 50-D CEC2017.

Func.	GA	PSO	HHO	DBO	POA	WOA	AO	TGCOA	ZOA	PO	MIPO	CGBPO	STPO
F1	7.848E-01	7.917E-01	1.550E+00	8.782E-01	8.828E-01	8.364E-01	3.610E+00	2.259E+00	2.347E+00	4.549E+01	1.289E+00	3.578E+00	1.012E+00
F3	7.453E-01	7.741E-01	1.569E+00	9.195E-01	8.294E-01	6.207E-01	1.541E+00	8.224E-01	8.818E-01	3.988E+01	1.278E+00	3.585E+00	9.480E-01
F4	7.423E-01	7.922E-01	1.542E+00	8.634E-01	8.481E-01	6.209E-01	1.541E+00	8.236E-01	9.003E-01	4.019E+01	1.315E+00	3.601E+00	9.465E-01
F5	1.065E+00	1.115E+00	2.399E+00	1.193E+00	1.510E+00	9.508E-01	2.209E+00	1.153E+00	1.565E+00	7.302E+01	1.926E+00	4.681E+00	1.325E+00
F6	1.876E+00	1.918E+00	4.338E+00	2.013E+00	3.058E+00	1.752E+00	3.771E+00	1.960E+00	3.111E+00	1.515E+02	3.455E+00	7.136E+00	2.121E+00
F7	1.069E+00	1.106E+00	2.428E+00	1.191E+00	1.541E+00	9.860E-01	2.241E+00	1.169E+00	1.572E+00	7.173E+01	1.930E+00	4.648E+00	1.318E+00
F8	1.081E+00	1.115E+00	2.456E+00	1.214E+00	1.538E+00	9.674E-01	2.227E+00	1.172E+00	1.595E+00	7.432E+01	1.992E+00	4.690E+00	1.321E+00
F9	1.091E+00	1.101E+00	2.488E+00	1.230E+00	1.528E+00	9.684E-01	2.247E+00	1.172E+00	1.587E+00	7.448E+01	1.952E+00	4.716E+00	1.287E+00
F10	1.286E+00	1.290E+00	2.951E+00	1.496E+00	1.884E+00	1.170E+00	2.619E+00	1.383E+00	1.974E+00	1.480E+02	5.534E+00	1.439E+01	1.504E+00
F11	2.312E+00	1.993E+00	4.640E+00	2.911E+00	2.880E+00	1.982E+00	4.414E+00	2.762E+00	3.026E+00	1.237E+02	3.539E+00	1.028E+01	3.014E+00
F12	2.412E+00	2.062E+00	5.219E+00	3.147E+00	3.415E+00	2.076E+00	5.498E+00	3.025E+00	3.566E+00	1.721E+02	4.895E+00	1.279E+01	3.264E+00
F13	2.107E+00	1.936E+00	5.037E+00	3.202E+00	3.064E+00	1.860E+00	4.377E+00	2.950E+00	3.240E+00	1.334E+02	3.978E+00	1.151E+01	2.830E+00
F14	3.148E+00	2.910E+00	6.836E+00	3.438E+00	5.008E+00	2.927E+00	5.980E+00	3.752E+00	5.048E+00	2.167E+02	5.675E+00	1.238E+01	3.647E+00
F15	2.194E+00	1.819E+00	4.450E+00	2.902E+00	2.971E+00	1.960E+00	4.363E+00	2.660E+00	2.795E+00	1.225E+02	3.608E+00	1.120E+01	2.947E+00
F16	2.265E+00	2.115E+00	5.118E+00	3.431E+00	3.405E+00	1.985E+00	4.934E+00	3.225E+00	3.560E+00	1.513E+02	4.587E+00	1.187E+01	2.773E+00
F17	4.352E+00	3.881E+00	9.637E+00	5.030E+00	7.368E+00	4.161E+00	8.428E+00	5.153E+00	7.135E+00	2.336E+02	2.775E+00	6.272E+00	5.308E+00
F18	1.077E+00	1.011E+00	2.148E+00	1.124E+00	1.288E+00	8.358E-01	1.980E+00	1.056E+00	1.334E+00	6.275E+01	1.716E+00	4.249E+00	1.290E+00
F19	5.484E+00	5.364E+00	1.282E+01	5.557E+00	9.904E+00	5.306E+00	1.062E+01	5.340E+00	9.901E+00	9.880E+02	2.206E+01	3.955E+01	5.619E+00
F20	4.400E+00	4.157E+00	9.347E+00	5.123E+00	6.819E+00	4.103E+00	8.690E+00	4.804E+00	6.916E+00	3.400E+02	7.662E+00	1.551E+01	4.859E+00
F21	4.619E+00	4.022E+00	9.770E+00	5.000E+00	7.191E+00	4.391E+00	8.542E+00	4.858E+00	7.437E+00	3.216E+02	7.179E+00	1.515E+01	5.015E+00
F22	4.363E+00	3.840E+00	9.632E+00	4.775E+00	6.998E+00	4.210E+00	8.185E+00	4.579E+00	6.946E+00	3.348E+02	7.627E+00	1.624E+01	4.600E+00
F23	4.666E+00	4.262E+00	1.015E+01	5.122E+00	7.705E+00	4.427E+00	9.004E+00	4.921E+00	7.662E+00	3.654E+02	8.131E+00	1.636E+01	5.024E+00
F24	3.007E+00	3.004E+00	6.924E+00	3.097E+00	5.238E+00	2.815E+00	6.002E+00	3.148E+00	5.330E+00	2.681E+02	5.592E+00	1.041E+01	3.243E+00
F25	2.337E+00	2.337E+00	5.243E+00	2.386E+00	3.919E+00	2.181E+00	4.632E+00	2.359E+00	3.999E+00	2.000E+02	4.314E+00	8.257E+00	2.501E+00
F26	3.302E+00	3.218E+00	7.404E+00	3.341E+00	5.739E+00	3.107E+00	6.483E+00	3.275E+00	5.771E+00	2.901E+02	6.036E+00	1.116E+01	3.411E+00
F27	3.682E+00	3.631E+00	8.417E+00	3.755E+00	6.473E+00	3.465E+00	7.235E+00	3.655E+00	6.554E+00	3.313E+02	6.795E+00	1.235E+01	3.785E+00
F28	3.028E+00	2.931E+00	6.636E+00	3.058E+00	5.229E+00	2.816E+00	5.912E+00	3.013E+00	5.283E+00	2.654E+02	5.633E+00	1.043E+01	3.141E+00
F29	2.339E+00	2.355E+00	5.418E+00	2.512E+00	4.031E+00	2.234E+00	4.729E+00	2.430E+00	4.070E+00	1.986E+02	4.311E+00	8.444E+00	2.572E+00
F30	6.226E+00	6.147E+00	1.453E+01	6.323E+00	1.156E+01	6.044E+00	1.234E+01	6.232E+00	1.164E+01	5.777E+02	1.166E+01	1.992E+01	6.349E+00

## C: Results of the ablation experiments on the CEC test set

Tables 26, 27, 28, 29 summarizes the ablation experiment results for the CEC2017 test set at 30 and 50 dimensions.The best average result among all comparison algorithms is shown in bold.

**Table 26:** Results of ablation experiments on 30-D CEC2017.

Func.	Metric	STPO	PO-APM	PO-EES	PO-DSP	PO
F1	std	3.84E+03	1.66E+04	1.63E+06	2.86E+07	7.23E+08
	mean	**3.31E+03**	1.68E+04	4.59E+05	1.20E+07	6.42E+08
F3	std	4.20E+03	3.05E-01	3.25E+03	4.24E+03	6.33E+03
	mean	1.23E+04	**3.01E+02**	7.70E+03	9.59E+03	1.46E+04
F4	std	3.46E+01	2.25E+01	3.11E+01	2.80E+01	5.60E+01
	mean	**4.82E+02**	4.90E+02	5.33E+02	5.36E+02	5.65E+02
F5	std	2.19E+01	3.78E+01	3.69E+01	3.49E+01	4.32E+01
	mean	**5.69E+02**	7.10E+02	6.85E+02	7.01E+02	7.28E+02
F6	std	3.53E+00	8.68E+00	8.63E+00	6.77E+00	7.87E+00
	mean	**6.02E+02**	6.52E+02	6.46E+02	6.52E+02	6.60E+02
F7	std	1.47E+01	8.59E+01	7.19E+01	6.95E+01	6.10E+01
	mean	**8.03E+02**	1.09E+03	1.03E+03	1.06E+03	1.16E+03
F8	std	1.36E+01	2.67E+01	2.78E+01	3.88E+01	3.95E+01
	mean	**8.66E+02**	9.62E+02	9.29E+02	9.52E+02	9.83E+02
F9	std	3.10E+02	5.30E+02	4.34E+02	8.20E+02	7.44E+02
	mean	**1.25E+03**	4.37E+03	3.38E+03	4.18E+03	5.74E+03
F10	std	9.61E+02	5.92E+02	8.53E+02	6.49E+02	6.34E+02
	mean	**4.96E+03**	5.44E+03	5.28E+03	5.44E+03	6.11E+03
F11	std	4.17E+01	4.64E+01	5.86E+01	1.69E+02	8.89E+01
	mean	**1.19E+03**	1.24E+03	1.26E+03	1.38E+03	1.33E+03
F12	std	1.44E+05	2.82E+05	9.52E+06	1.16E+06	4.20E+07
	mean	**1.38E+05**	3.42E+05	8.57E+06	7.99E+05	4.14E+07
F13	std	1.98E+04	2.86E+04	7.66E+04	8.27E+03	6.63E+04
	mean	2.27E+04	3.41E+04	1.22E+05	**1.40E+04**	1.14E+05
F14	std	1.92E+04	1.67E+03	1.37E+03	4.76E+03	4.16E+04
	mean	1.81E+04	2.96E+03	**2.58E+03**	7.35E+03	5.94E+04
F15	std	1.35E+04	1.19E+04	9.01E+03	1.08E+04	4.15E+04
	mean	1.17E+04	1.12E+04	1.78E+04	**8.18E+03**	6.61E+04
F16	std	2.79E+02	2.69E+02	2.55E+02	3.87E+02	4.82E+02
	mean	**2.47E+03**	3.10E+03	2.90E+03	3.13E+03	3.11E+03

**Table 27:** Results of ablation experiments on 30-D CEC2017. (continued).

Func.	Metric	STPO	PO-APM	PO-EES	PO-DSP	PO
F17	std	1.56E+02	2.91E+02	2.39E+02	3.63E+02	2.68E+02
	mean	**2.06E+03**	2.49E+03	2.50E+03	2.65E+03	2.31E+03
F18	std	6.75E+04	3.41E+04	2.75E+04	1.05E+05	7.33E+05
	mean	9.99E+04	**4.87E+04**	5.76E+04	9.46E+04	9.03E+05
F19	std	7.20E+03	7.61E+03	3.14E+04	7.25E+03	9.08E+05
	mean	1.01E+04	1.09E+04	3.85E+04	**8.66E+03**	1.72E+06
F20	std	1.03E+02	2.07E+02	1.85E+02	2.32E+02	1.77E+02
	mean	**2.33E+03**	2.75E+03	2.58E+03	2.54E+03	2.56E+03
F21	std	1.18E+01	8.04E+02	5.48E+02	7.92E+02	5.59E+01
	mean	**2.35E+03**	2.90E+03	2.52E+03	3.21E+03	2.51E+03
F22	std	1.41E+00	1.63E+03	1.90E+03	1.75E+03	2.15E+03
	mean	**2.30E+03**	1.43E+04	1.43E+04	1.47E+04	3.57E+03
F23	std	3.59E+01	5.92E+02	6.51E+02	6.32E+02	6.00E+01
	mean	**2.72E+03**	5.76E+03	4.92E+03	5.72E+03	2.96E+03
F24	std	3.63E+01	1.17E+03	1.37E+03	1.26E+03	7.92E+01
	mean	**2.91E+03**	4.74E+03	4.52E+03	4.33E+03	3.14E+03
F25	std	1.72E+01	4.01E+00	2.52E+01	4.16E+01	3.30E+01
	mean	2.90E+03	**2.88E+03**	2.94E+03	2.94E+03	2.99E+03
F26	std	9.00E+02	2.17E+03	2.19E+03	1.78E+03	1.13E+03
	mean	**4.18E+03**	6.20E+03	6.06E+03	6.77E+03	6.58E+03
F27	std	2.11E+01	8.54E+01	5.15E+01	1.02E+02	5.94E+01
	mean	**3.24E+03**	3.36E+03	3.32E+03	3.35E+03	3.33E+03
F28	std	4.75E+01	5.63E+01	3.79E+01	3.07E+01	4.82E+01
	mean	**3.18E+03**	**3.18E+03**	3.30E+03	3.30E+03	3.35E+03
F29	std	1.80E+02	2.81E+02	3.53E+02	4.39E+02	3.24E+02
	mean	**3.73E+03**	4.65E+03	4.57E+03	4.38E+03	4.78E+03
F30	std	4.24E+03	2.15E+04	4.04E+05	4.16E+03	6.86E+06
	mean	**9.67E+03**	3.48E+04	4.22E+05	1.20E+04	9.96E+06
Avg. rank:		**1.67**	2.98	3.02	3.33	4.00

**Table 28:** Results of ablation experiments on 50-D CEC2017.

Func.	Metric	STPO	PO-APM	PO-EES	PO-DSP	PO
F1	std	3.17E+03	1.58E+05	1.31E+09	3.55E+09	1.95E+09
	mean	**1.86E+03**	2.82E+05	2.03E+09	3.59E+09	5.16E+09
F3	std	1.74E+04	2.71E+03	1.13E+04	2.52E+04	1.22E+04
	mean	7.60E+04	**7.45E+03**	4.56E+04	6.95E+04	5.38E+04
F4	std	4.42E+01	5.84E+01	1.63E+02	1.79E+02	3.62E+02
	mean	**5.17E+02**	5.24E+02	9.17E+02	9.17E+02	1.23E+03
F5	std	1.83E+01	5.45E+01	3.66E+01	4.74E+01	4.64E+01
	mean	**6.58E+02**	8.49E+02	7.97E+02	8.39E+02	9.31E+02
F6	std	4.17E+00	4.06E+00	4.36E+00	5.96E+00	4.49E+00
	mean	**6.09E+02**	6.62E+02	6.55E+02	6.61E+02	6.68E+02
F7	std	5.81E+01	9.80E+01	9.71E+01	1.26E+02	9.83E+01
	mean	**9.44E+02**	1.57E+03	1.43E+03	1.53E+03	1.71E+03
F8	std	3.13E+01	4.62E+01	5.46E+01	5.20E+01	5.55E+01
	mean	**9.56E+02**	1.17E+03	1.12E+03	1.16E+03	1.21E+03
F9	std	1.45E+03	1.51E+03	1.68E+03	2.19E+03	2.67E+03
	mean	**3.45E+03**	1.21E+04	1.06E+04	1.19E+04	1.67E+04
F10	std	1.27E+03	9.92E+02	8.80E+02	1.16E+03	1.23E+03
	mean	**7.44E+03**	8.45E+03	8.37E+03	9.36E+03	1.01E+04
F11	std	4.58E+01	7.10E+01	2.87E+02	1.00E+03	2.15E+02
	mean	**1.27E+03**	1.35E+03	1.89E+03	3.06E+03	2.01E+03
F12	std	1.45E+06	3.53E+06	1.17E+08	4.94E+07	3.91E+08
	mean	**1.83E+06**	5.75E+06	1.09E+08	4.66E+07	4.29E+08
F13	std	1.04E+04	2.14E+04	6.25E+04	3.50E+05	2.55E+07
	mean	**1.07E+04**	3.69E+04	1.01E+05	1.16E+05	1.16E+07
F14	std	5.53E+04	6.60E+03	1.80E+04	5.78E+04	3.40E+05
	mean	**1.15E+05**	1.25E+04	2.91E+04	6.08E+04	5.71E+05
F15	std	3.98E+03	1.79E+04	3.99E+04	8.04E+03	1.35E+06
	mean	**8.29E+03**	1.77E+04	5.65E+04	1.22E+04	6.50E+05
F16	std	4.12E+02	5.00E+02	4.31E+02	6.88E+02	4.72E+02
	mean	**3.08E+03**	3.74E+03	3.85E+03	3.94E+03	4.93E+03

**Table 29:** Results of ablation experiments on 50-D CEC2017. (continued).

Func.	Metric	STPO	PO-APM	PO-EES	PO-DSP	PO
F17	std	2.22E+02	3.01E+02	3.13E+02	5.18E+02	6.40E+02
	mean	**2.78E+03**	3.67E+03	3.55E+03	3.54E+03	3.99E+03
F18	std	5.85E+05	5.62E+04	3.26E+05	6.64E+05	1.84E+06
	mean	5.11E+05	**1.24E+05**	3.55E+05	5.15E+05	2.68E+06
F19	std	7.10E+03	1.36E+04	4.97E+05	1.03E+04	2.16E+06
	mean	**1.23E+04**	2.53E+04	3.30E+05	2.12E+04	2.76E+06
F20	std	2.54E+02	3.44E+02	3.54E+02	3.59E+02	2.61E+02
	mean	**2.97E+03**	3.44E+03	3.19E+03	3.19E+03	3.43E+03
F21	std	3.34E+01	9.36E+02	1.42E+03	1.16E+03	8.77E+01
	mean	**2.43E+03**	5.37E+03	4.71E+03	5.49E+03	2.75E+03
F22	std	3.29E+03	2.38E+03	2.27E+03	3.10E+03	1.08E+03
	mean	**9.04E+03**	2.49E+04	2.44E+04	2.56E+04	1.17E+04
F23	std	6.58E+01	1.55E+03	1.33E+03	1.13E+03	1.29E+02
	mean	**2.91E+03**	9.02E+03	8.23E+03	8.66E+03	3.39E+03
F24	std	5.53E+01	4.08E+02	3.38E+02	4.63E+02	1.73E+02
	mean	**3.10E+03**	7.67E+03	7.67E+03	7.81E+03	3.57E+03
F25	std	2.58E+01	4.15E+01	8.48E+01	2.75E+02	1.89E+02
	mean	3.07E+03	**3.05E+03**	3.41E+03	3.43E+03	3.56E+03
F26	std	1.49E+03	2.53E+03	1.15E+03	2.43E+03	1.61E+03
	mean	**5.20E+03**	1.09E+04	1.13E+04	1.11E+04	1.21E+04
F27	std	8.21E+01	2.46E+02	2.62E+02	2.83E+02	2.96E+02
	mean	**3.46E+03**	4.14E+03	4.08E+03	4.16E+03	4.01E+03
F28	std	3.59E+01	2.57E+01	2.82E+02	3.26E+02	2.95E+02
	mean	3.33E+03	**3.32E+03**	4.10E+03	3.95E+03	4.18E+03
F29	std	4.61E+02	7.40E+02	7.81E+02	6.91E+02	6.95E+02
	mean	**4.24E+03**	5.60E+03	6.23E+03	6.01E+03	6.70E+03
F30	std	3.75E+05	7.06E+05	3.30E+07	5.80E+05	3.41E+07
	mean	**1.12E+06**	1.90E+06	7.14E+07	1.77E+06	1.41E+08
Avg. rank:		**1.51**	2.74	2.98	3.68	4.09

## D: Basic information regarding the selected engineering design issues

### D.1: Cantilever beam design problem (CBDP)

The formulation of the cantilever beam design problem is given in Equation (22).

22 $$\begin{aligned} \begin{aligned} \min \ f(\textbf{X})&= 0.0624(x_1+x_2+x_3+x_4+x_5),\\ \text {s.t.}\quad g(\textbf{X})&= \frac{61}{x_1^3}+\frac{37}{x_2^3}+\frac{19}{x_3^3}+\frac{7}{x_4^3}+\frac{1}{x_5^3}-1 \le 0,\\ 0.01 \le x_i&\le 100,\quad i=1,\ldots ,5 . \end{aligned} \end{aligned}$$

The model for cantilever beam design problems is shown below (Fig. 34):

### D.2: Three-bar truss design problems (TBTDP)

The formula for the three-bar truss design problem is shown in Equation (23).

23 $$\begin{aligned} \begin{aligned} \vec {x}&= [x_1\ \ x_2]=[A_1\ \ A_2],\\ \min \ f(\vec {x})&= (2\sqrt{2}\,x_1+x_2)\,l,\\ \text {s.t.}\quad g_1(\vec {x})&= \frac{\sqrt{2x_1+x_2}}{\sqrt{2x_1^2+2x_1x_2}}\,P-\sigma \le 0 . \end{aligned} \end{aligned}$$

The model for the three-bar truss design problem is shown below (Fig. 35):

### D.3: Tubular Column Design Problems (TCDP)

The formula for the tubular column design problem (TCDP) is shown in Equation (24).

24 $$\begin{aligned} \begin{aligned} \min \ f(X)&= 9.8x_1x_2 + 2x_1,\\ \text {s.t.}\quad g_1(X)&= \frac{P}{\pi x_1 x_2 \sigma _y} - 1 \le 0,\\ g_2(X)&= \frac{8PL^2}{\pi ^3 E x_1 x_2 (x_1^2 + x_2^2)} - 1 \le 0,\\ g_3(X)&= \frac{2.0}{x_1} - 1 \le 0,\\ g_4(X)&= \frac{x_1}{14} - 1 \le 0,\\ g_5(X)&= \frac{0.2}{x_2} - 1 \le 0,\\ g_6(X)&= \frac{x_2}{8} - 1 \le 0,\\ 2 \le x_1&\le 14,\quad 0.2 \le x_2 \le 0.8. \end{aligned} \end{aligned}$$

The model for the tubular column design problem is shown below (Fig. 36):

### D.4: Piston link design problems (PLDP)

The formula for piston Link design issues is shown in Equation (25).

25 $$\begin{aligned} \begin{aligned} \min \ f(X)&= \frac{1}{4}\pi x_3^{2}(L_2-L_1),\\ \text {s.t.}\quad g_1(X)&= QL\cos \theta - R\,F \le 0,\\ g_2(X)&= Q(L-x_4)-M_{\max } \le 0,\\ g_3(X)&= 1.2(L_2-L_1)-L_1 \le 0,\\ g_4(X)&= \frac{x_3}{2}-x_2 \le 0,\\ R&= \frac{\left| -x_4(x_4\sin \theta +x_1)+x_1(x_2-x_4\cos \theta )\right| }{\sqrt{(x_4-x_2)^2+x_1^2}},\\ F&= \frac{\pi P x_3^{2}}{4},\\ L_2&= \sqrt{(x_4\sin \theta +x_1)^2+(x_2-x_4\cos \theta )^2},\\ \theta&= 45^\circ ,\\ Q&= 10{,}000~\textrm{lbs},\quad L=240~\textrm{in},\quad M_{\max }=1.8\times 10^{6}~\textrm{lbs}\cdot \textrm{in},\quad P=1500~\textrm{psi},\\ 0.05 \le x_1,x_2,x_4&\le 500,\qquad 0.05 \le x_3 \le 120. \end{aligned} \end{aligned}$$

The model for the piston Link design issue is shown below (Fig. 37):

### D.5: Weight minimization design problem for speed reducers(WMSR)

The formula for the weight minimization design problem of the reducer is shown in Equation (26).

26 $$\begin{aligned} \begin{aligned} \min \ f(X)&= 0.7854x_1x_2^2\left( 14.9334x_3-43.0934+3.3333x_3^2\right) +0.7854\left( x_4x_6^2+x_5x_7^2\right) \\&\quad -1.508x_1\left( x_6^2+x_7^2\right) +7.477\left( x_6^3+x_7^3\right) ,\\ \text {s.t.}\quad g_1(X)&= -x_1x_2^2x_3 + 27 \le 0,\\ g_2(X)&= -x_1x_2^2x_3^2 + 397.5 \le 0,\\ g_3(X)&= -x_2x_4^2x_3 + 1.93 \le 0,\\ g_4(X)&= -x_2x_5^2x_3 + 1.93 \le 0,\\ g_5(X)&= 10x_6^{-3}\sqrt{16.91\times 10^6+\left( 745x_4x_2^{-1}x_3^{-1}\right) ^2}-1100 \le 0,\\ g_6(X)&= 10x_7^{-3}\sqrt{157.5\times 10^6+\left( 745x_5x_2^{-1}x_3^{-1}\right) ^2}-850 \le 0,\\ g_7(X)&= x_2x_3 - 40 \le 0,\\ g_8(X)&= -x_1x_2^{-1} + 5 \le 0,\\ g_9(X)&= x_1x_2^{-1} - 12 \le 0,\\ g_{10}(X)&= 1.5x_6 - x_4 + 1.9 \le 0,\\ g_{11}(X)&= 1.1x_7 - x_5 + 1.9 \le 0,\\ 0.7 \le x_2&\le 0.8,\qquad 17 \le x_3 \le 28,\qquad 2.6 \le x_1 \le 3.6,\\ 5 \le x_4&\le 5.5,\qquad 7.3 \le x_5 \le 8.3,\qquad 2.9 \le x_6 \le 3.9. \end{aligned} \end{aligned}$$

The model for the gear reducer weight minimization design problem is shown below (Fig. 38):
